# Strain-engineered fabrication of high-density aligned graphene-based nanoscrolls via UV-tunable adhesive tapes

**DOI:** 10.1038/s41467-026-78264-8

**Published:** 2026-10-21

**Authors:** Maki Nakatani, Akari Sudo, Pablo Solís-Fernández, Haiming Sun, Satoru Fukamachi, Tomoki Maezawa, Tatsuya Ikuta, Takako Matsunaga, Aika Uchida, Masatoshi Mitsuhara, Yung-Chang Lin, Koji Takahashi, Rika Matsumoto, Kazu Suenaga, Takahiro Yamamoto, Hiroki Ago

**Affiliations:** 1 Faculty of Engineering Sciences, Kyushu University, Kasuga, Fukuoka 816-8580 Japan; 2 Department of Physics, Tokyo University of Science, Kagurazaka, Shinjuku, Tokyo, 162-8602 Japan; 3 SANKEN (The Institute of Scientific and Industrial Research), The University of Osaka, Osaka, 567-0047 Japan; 4 Interdisciplinary Graduate School of Engineering Sciences, Kyushu University, Kasuga, Fukuoka 816-8580 Japan; 5 Department of Aeronautics and Astronautics, Kyushu University, Fukuoka, 819-0395 Japan; 6 Nanomaterials Research Institute, National Institute of Advanced Industrial Science and Technology (AIST), Tsukuba, 305-8565 Japan; 7 Faculty of Engineering, Tokyo Polytechnic University, Atsugi, Kanagawa 243-0297 Japan; 8 Center for Semiconductor and Device Ecosystem (CSeDE), Kyushu University, Kasuga, Fukuoka 816-8580 Japan

**Keywords:** Mechanical and structural properties and devices, Synthesis and processing

## Abstract

Graphene nanoscrolls (GNSs), one-dimensional nanostructures formed by rolling up a single sheet of graphene, exhibit electronic and mechanical properties distinct from the original graphene sheet. Although several methods have been proposed to synthesize GNSs, achieving high-density GNSs with controlled scroll chirality and direction remains challenging. Here, we demonstrate the strain-engineered fabrication of large-scale GNSs using an ultraviolet (UV) light-tunable adhesive tape. The UV tape is stretched to apply tensile strain to graphene stacked on it, and releasing the applied strain by detaching the graphene from the tape results in the formation of GNSs. This strain-assisted method gives isolated GNSs of increased density ( ≈ 380 nanoscrolls per mm) and length (90 μm). This method also allows control of the scroll angle, i.e., chiral angle, by controlling the stretch direction of the tape/graphene stack. The nanoscroll structure allows the intercalation of metal chloride molecules, resulting in an increase of the electrical conductivity. Furthermore, heteronanoscrolls of graphene and MoS_2_ are realized by stretching MoS_2_/graphene heterostacks, exhibiting clear optical response. Our controlled synthesis of GNSs and heteronanoscrolls provides an opportunity to manipulate nanomaterials through the strain engineering with the functional UV tape.

## Abstract (web summary)

Graphene nanoscrolls exhibit appealing electronic and mechanical properties; however, high-density fabrication with controlled scroll chirality and orientation remains challenging. Here, the authors demonstrate a strain-engineered fabrication method utilizing an ultraviolet light-tunable adhesive tape.

## Introduction

Graphene is a two-dimensional (2D) material that exhibits exotic physical properties and possesses many potential applications in various fields^1,2^. Modifying graphene through morphological deformations, such as buckling^3^, folding^4,5^, introduction of *sp*^3^ bonds^6,7^, or through dimensional reduction into nanoribbons^8,9^ allows to tune the electronic, optical, and mechanical properties of the original flat graphene sheet. Confining electrons in one-dimensional (1D) graphene structures significantly changes the electronic band structure due to quantum confinement effects, which are dependent on the width, edges, and symmetry^10,11^.

Graphene nanoscrolls (GNSs) are a distinctive type of 1D graphene, made by rolling up of a graphene sheet^12,13^. As they are formed from a single continuous graphene sheet, the whole GNS can contribute to carrier transport unlike multiwalled carbon nanotubes (MWCNTs), in which only the most outer layer contributes to the current flow. Thus, a GNS can attain a high current density^14^. Theoretical studies predict exceptional electronic and optical properties, which are dependent on the topology of a GNS, such as chirality and diameter^15–18^. Each GNS has a single chirality through outer to inner layers, determined by the rolling angle. This differs from MWCNTs, because each layer of MWCNT has different chiral angle and electronic band structure^19^. Moreover, due to their high mechanical flexibility with expandable interlayer space, GNSs are promising for energy storage^20–24^. Theoretical simulations predict that GNS can store hydrogen molecules both between graphene layers and in the inner core^23,24^. GNSs made from graphene oxide (GO) have been applied to supercapacitors, obtaining both high capacity and stability^20–22^. In contrast, it is difficult to intercalate molecules or ions in the interlayer nanospace of MWCNTs, because of their rigid structure. Therefore, GNSs offer a 1D graphene platform with intriguing physical properties and many applications.

There are two main approaches to synthesize nanoscrolls. The first method involves sonication or stirring graphite intercalation compounds (GICs), GO, or graphene flake dispersions in solvents, eventually forming GNSs due to van der Waals interaction between adjacent turns of the rolled sheet^20,21,25–27^. The second method is to intercalate solvents, such as ethanol, water, or their mixture, between graphene and an underlying SiO_2_ substrate^14,18,28^. The solvent induces the detachment of an edge of the graphene flake, leading to the formation of the nanoscroll structure. However, the density of GNSs formed by these methods is low, and the scroll direction is generally random.

In this work, we demonstrate large-area synthesis of dense GNSs with controlled scroll direction by utilizing a functional tape^29^ whose adhesive strength can be tuned with ultraviolet (UV) light. The UV tape is stretched to apply tensile strain to graphene supported on it. Scrolling occurs when the graphene is detached from the tape surface, resulting in the formation of GNSs aligned perpendicular to the direction of the applied strain. The atomically thin layered structure of graphene makes its direct manipulation challenging. However, our UV tape enables the controlled manipulation of a graphene to form nanoscroll structures over large areas. The nanoscroll structure allows intercalation of metal chloride molecules and alkali metal, significantly increasing the electrical conductivity. This method was further extended to the synthesis of heterostructures of GNS and MoS_2_, providing a route to make 1D heterostructures.

## Results

### Strain-induced formation of GNSs

**Fig. 1:**
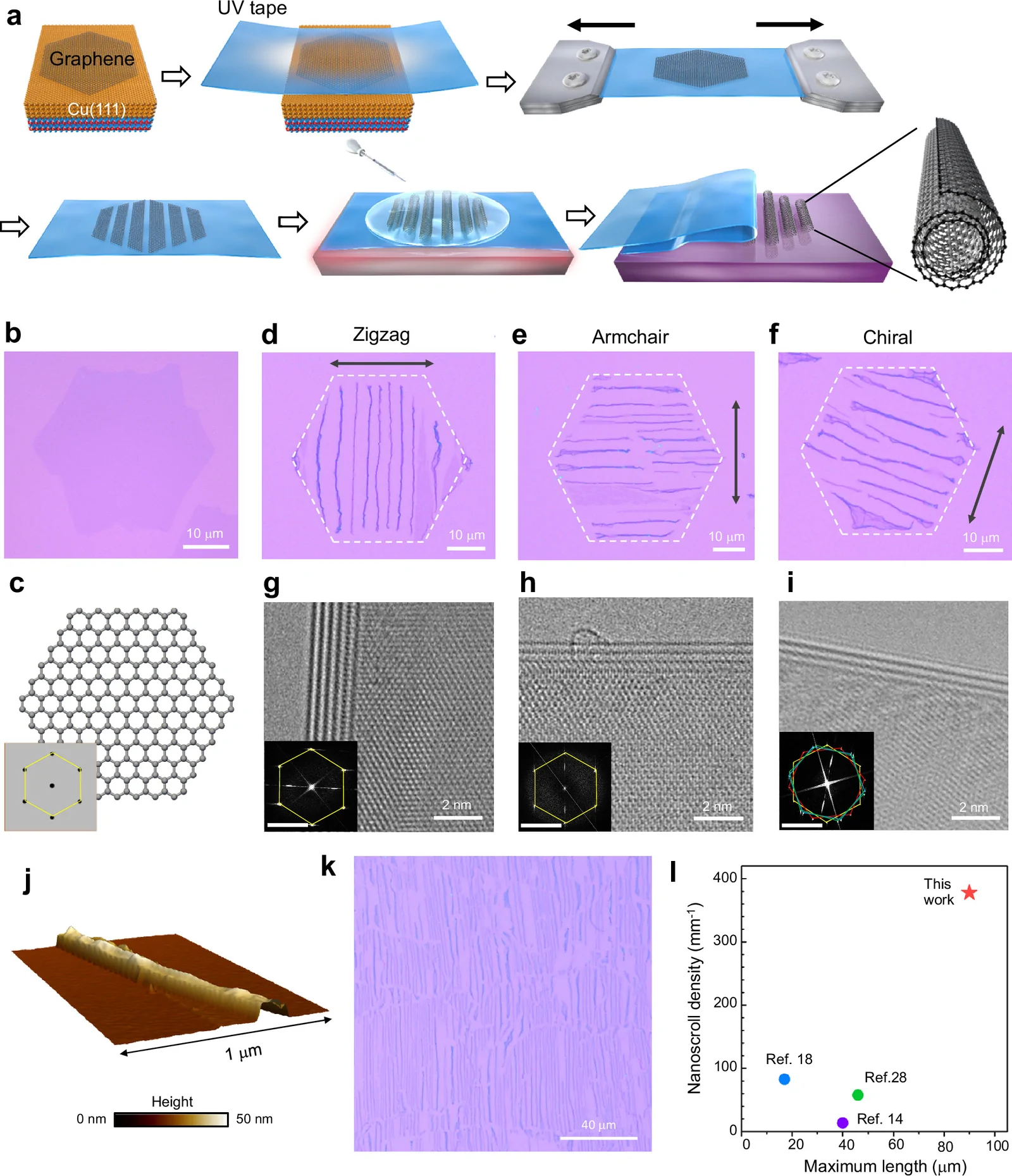
Controlled synthesis of a high-density, aligned GNS array. **a** Schematic of the synthesis process of GNSs using the UV tape. **b** Hexagonal grain of monolayer graphene transferred on a SiO_2_ substrate without applying strain. **c** Schematic atomic structure and corresponding simulated diffraction pattern (inset) of the hexagonal grain shown in (**b**)**. d–f** GNSs made by stretching the UV tape/graphene stack along the directions indicated by the arrows. (**d**) zigzag GNSs, (**e**) armchair GNSs, and (**f**) chiral GNSs. **g–i** Top-view high-resolution TEM images of the corresponding GNSs. Insets are the FFT patterns, with the scale bars representing 5 nm^-1^. **j** AFM height image of a GNS. **k** Large array of aligned GNSs made from a continuous graphene sheet. **l** Comparison of the density and height of isolated GNSs. Purple, blue, and green labels indicate the data from refs. ^14,18,28^, respectively. Source data are provided as a Source Data file.

Fig. 1a illustrates the synthesis procedure of aligned GNSs on a SiO_2_ substrate assisted by UV tape. This tape was optimized for the facile and clean transfer of graphene onto various substrates^29^. First, monolayer graphene was transferred onto the UV tape, as described in the Methods section. The ends of the UV tape were then fixed with metal clamps and mechanically extended up to 15% to apply tensile strain (see Supplementary Fig. 1 for the experimental setup). The strong interfacial adhesion with the tape ensures efficient strain transfer to the graphene, forming periodic linear cracks in the graphene, oriented perpendicular to the strain direction (Supplementary Fig. 2). Upon release of the tensile strain from the UV tape, the stored strain energy in the graphene promoted partial detachment of cracked edged form the tape surface, initiating the rolling of graphene into GNS. This scrolling process can be triggered and aided by different stimuli, such as heating on a hotplate, microwave heating, by dropping solvent, or by the transfer to the SiO_2_ itself. We found that a combination of dropping organic solvent while heating the tape is the most effective to obtain high-density GNSs (Supplementary Fig. 3).

Fig. 1b shows an optical image of a hexagonal graphene grain transferred onto a SiO_2_ substrate without applying strain. As reported previously, the UV tape transfer results in a graphene surface free from adhesive residue and cracks^29^. In a separate preparation, tensile strain was applied to the hexagonal graphene grains along their edges while they were supported on UV tape. After transfer onto a SiO_2_ substrate, we observed highly aligned GNSs, with the hexagonal outline of the original grain still discernible (Fig. 1d). As shown in Fig. 1c, the hexagonal graphene grains are known to be terminated with zigzag edges^30^, thus the resulting nanoscrolls of Fig. 1d are aligned perpendicular to the zigzag direction. As the cracks in graphene form perpendicular to the applied strain, the orientation of the rolled structures can be controlled by adjusting the stretching direction of the graphene/tape stack, allowing to synthesize GNSs with zigzag, armchair, and chiral structures (Fig. 1d–f).

To confirm the chirality-selective formation of nanoscrolls, the as-prepared GNS samples were further characterized using high-resolution electron microscopes. Fig. 1g–i displays the top-view transmission electron microscopy (TEM) images of the nanoscrolls, together with their corresponding FFT patterns shown in the insets (the corresponding high-resolution scanning transmission electron microscopy (STEM) images are displayed in Supplementary Fig 4). In Fig. 1g–i, we observe a layered structure reflecting the rolling structure. The FFT patterns shown in the insets confirmed the formation of zigzag and armchair nanoscrolls, which are solely determined by the stretch direction. It is worth noting that it is difficult to grow chirality-controlled carbon nanotubes, while our tensile-based method allows for making zigzag and armchair scrolls in a highly controlled manner. In addition, due to the scrolling structure, all the rolls have the same chirality, differing from MWCNTs in which each layer has a different chirality^19^. Chiral GNS exhibited complex FFT patterns (inset of Fig. 1i), with the number of the set of spots almost corresponding to the number of rolls. Although the origin of the multiple patterns is not clear, it may reflect a cone-like chiral nanoscroll structure. The possibility of the chirality control, including zigzag and armchair, is an advantage of our strain-assisted nanoscroll formation, which offers opportunities to explore chiral-related phenomena.

The atomic force microscope (AFM) image of an isolated GNS is displayed in Fig. 1j, while Supplementary Fig. 5 provides wider-area AFM images of aligned nanoscrolls made from a hexagonal graphene grain and a height profile. The height of the nanoscroll is typically within the 10-40 nm range, reflecting the formation of a three-dimensional (3D) scrolling structure.

Beyond a single hexagonal grain, most of the hexagonal grains covering an area of ≈1×1 cm^2^ can be converted to aligned GNS arrays (Supplementary Fig. 6). This indicates that the strain is uniformly applied to graphene across the tape surface, allowing the formation of GNSs from large-area graphene films in addition to isolated grains. As shown in Fig. 1k, a large array of GNSs was obtained from a uniform sheet of monolayer graphene grown on Cu(111). The GNS density reaches a value of ≈380 scrolls per mm with scrolls as long as 90 μm (Supplementary Fig. 7). As shown in Fig. 1l, both the density and length of the present GNSs far exceed those reported before. Previous reports typically show optical images covering only limited areas^28^, or show partially scrolled nanoscrolls that are not isolated from the original graphene flakes (i.e., scroll occurred only at the flake edges)^18^. On the other hand, our strain-assisted formation of GNSs produces almost complete scroll structures without un-scrolled areas. The above results indicate the effectiveness of our strain-engineered GNS synthesis in large areas. In addition, unlike the randomly oriented and bundled GNSs produced by solvent-assisted synthesis^14,28^, our method offers high controllability of scrolling angle and orientation.

### Mechanism of the nanoscroll formation

Controlling the adhesion force between the UV tape and graphene is essential for the realization of a high-density GNS array. If the adhesion force is too strong, graphene cannot be detached despite the residual tensile strain. On the other hand, a weak adhesion force cannot effectively apply tensile strain, as graphene would slip on the tape surface. In our case, the tape was illuminated by UV light after it was adhered on as-grown graphene, followed by delaminating graphene from the Cu by the electrochemical bubbling^29^. Thus, the adhesion of the tape is sufficiently weak in the delaminated tape/graphene stack, but strong enough to transfer the strain efficiently to the monolayer graphene. The strained graphene can be detached from the UV tape by further reduction of tape-graphene coupling by additional stimulus to form GNSs, as mentioned later.

**Fig. 2:**
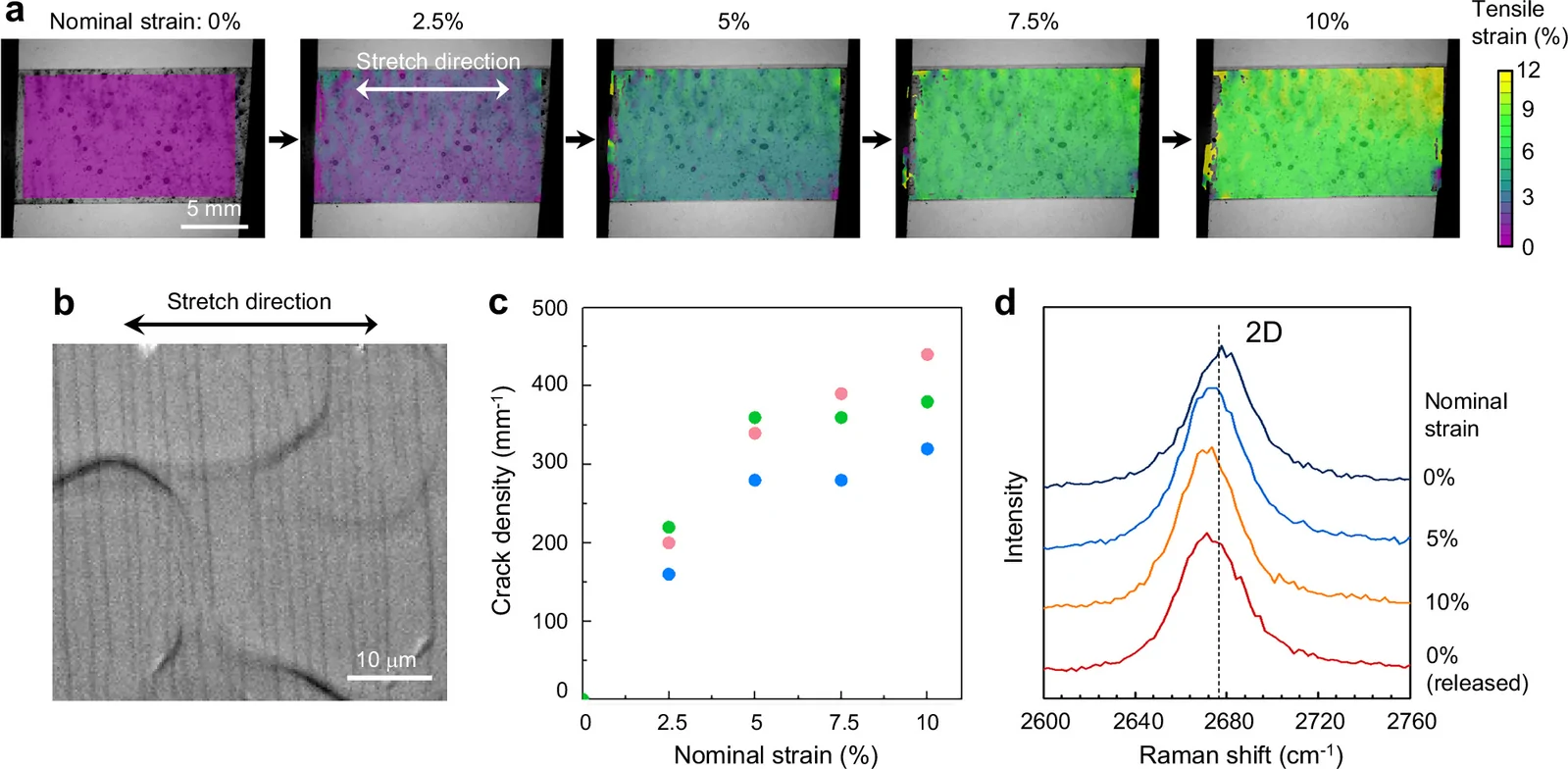
In-situ measurement of tape deformation, crack formation, and Raman spectra. **a** DIC analysis of the actual tensile strain applied to the graphene/tape stack. The nominal strain is calculated from the change of the tape length, and the tensile strain was determined from the changes of each position of the stack. **b** Laser confocal microscope image of the graphene on the UV tape, captured while applying a nominal strain of 10%. The strain-induced crack formation in graphene was confirmed by independent experiments for more than 10 times. **c** Evolution of crack density of graphene with nominal tensile strain, measured at three different positions. **d** in-situ Raman spectra measured while applying the strain, and after releasing the nominal strain applied to the stack. Source data are provided as a Source Data file.

To fully understand the mechanism of the nanoscroll formation, we first studied the actual tensile strain that is applied to the UV tape during the mechanical extension of the graphene/tape stack. Fig. 2a, Supplementary Fig. 8, and Supplementary Movie 1 show the strain distribution in the UV tape determined by a digital image correlation (DIC) method (the details of the measurement are described in Supplementary Fig. 8). As seen in Fig. 2a, the DIC result confirmed that the measured tensile strain is consistent with the strain calculated from the extended length of the stack. However, when the tensile was increased to ≈10%, some inhomogeneity was observed due to the deformation of the UV tape.

Next, we determined the crack formation in the graphene sheet using a laser confocal microscope while applying strain. As shown in Fig. 2b, Supplementary Figs. 9 and 10, extending the stack induces cracks that are oriented normal to the applied strain. The crack density increased with the strain up to ≈10% and then almost saturates up to 15% (Fig. 2c and Supplementary Fig. 9). The highest crack density reached 440 mm^-1^, corresponding to an average ribbon width of 2.3 μm, with each of these graphene ribbons producing a single nanoscroll after detaching from the tape surface.

It is also important to measure the actual strain transferred to the graphene sheet from the UV tape, because the crack formation in graphene and possible slippage can reduce the actual strain. Therefore, we measured in-situ Raman spectra of the graphene while extending the graphene/tape stack. Fig. 2d shows the evolution of the Raman 2D band with respect to the nominal strain (more detailed data can be found in Supplementary Figs. 11, 12). The strained graphene exhibited downshift of the 2D band when the stack was extended. Part of this shift is retained even after releasing the strain of the stack. This result indicates that the graphene experiences tensile strain even after relaxing the UV tape. This residual strain is supposed to trigger the nanoscroll formation.

To investigate the role of the additional stimuli employed to produce the GNSs, we measured the adhesion force of the UV tape after different treatments, including UV exposure, solvent treatment, and heating (Supplementary Fig. 13). The pristine UV tape showed a high adhesion force (0.12 N mm^-1^), which decreased to 0.015 N mm^-1^ after exposure to UV light. Post-UV heating and solvent treatment further decreased the adhesion force to 0.009 N mm^-1^, with toluene being the most effective among the studied solvents (Supplementary Fig. 13c).

Therefore, after applying and releasing the tensile strain, the graphene/tape stack was treated with toluene while being heated on a hot plate. The reduced adhesion eases the local detachment of graphene from the tape surface, enabling the graphene to roll up into nanoscrolls. We think that the contact with the solvent not only weakens the adhesion force of the UV tape but also promotes the scrolling through intercalation at the strained graphene and tape interface, a mechanism that has been widely used to make graphene scrolls^14,18,28^. Because the crack orientation in graphene is determined by the applied tensile strain, scrolling preferentially initiates at these predefined edges. Consequently, the scrolling direction and chirality are deterministically controlled, allowing for large arrays of aligned GNSs.

### Characterization of GNSs

**Fig. 3:**
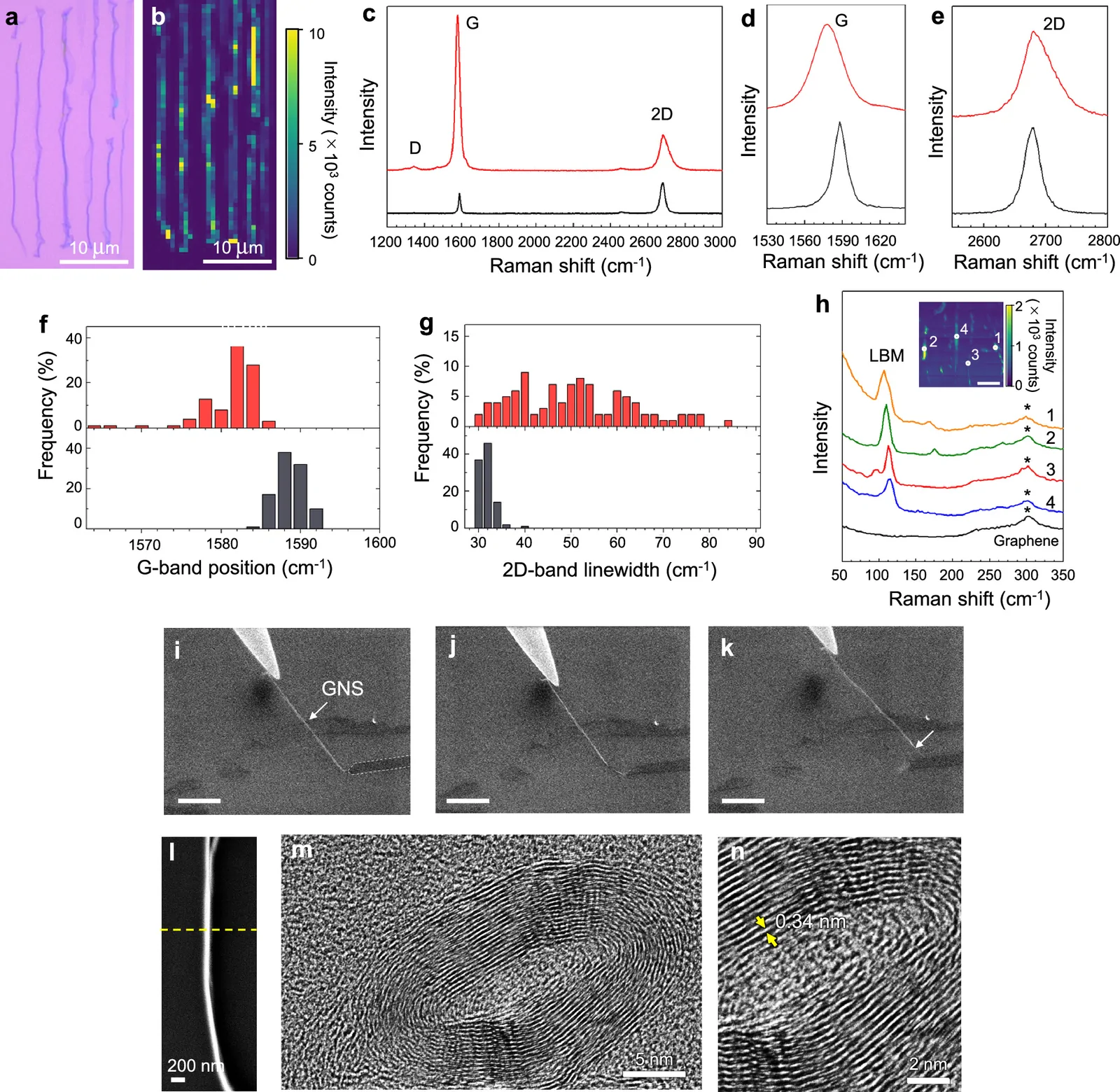
Characterization of aligned GNSs. **a**, **b** Optical image (**a**) and the corresponding Raman G band intensity mapping (**b**) of aligned GNSs on a SiO_2_ substrate. **c** Comparison of the Raman spectra of a GNS (red) and monolayer graphene (black). Normalized G (**d**) and 2D bands (**e**). Distributions of G band position (**f**) and 2D band linewidth (**g**). **h** Low-frequency Raman spectra of four different GNRs. The Raman spectrum of a monolayer graphene sheet is also shown for comparison (black). The inset shows the intensity mapping of the LBM peak (scale bar: 5 μm). **i–k** in-situ SEM observation of the structural change of a transferred graphene fragment while being detached from the SiO_2_ surface. Scale bars: 5 μm. **i** GNS was lifted out from the SiO_2_ surface with a probe tip. **j** After the graphene flake gradually detaches from the SiO_2_ surface, it immediately starts to scroll, owing to the residual tensile strain in the graphene flake. Three independent experiments were performed and Supplementary Movie 2 shows representative results. **k** Further pulling breaks the graphene, detaching the GNS from it. The arrow in **k** indicates one end of the GNS. **l** SEM image of the GNS used for the cross-sectional TEM imaging. **m, n** Cross-sectional TEM images of a GNS. Source data are provided as a Source Data file.

We transferred as-grown GNSs from the UV tape to a SiO_2_ substrate for further characterizations. As shown in Fig. 3a, b, the Raman mapping image of the G-band intensity confirmed that the nanoscrolls are made from graphene. Fig. 3c and Supplementary Fig. 14 compare the typical Raman spectra of the GNS and monolayer graphene, both transferred using the UV tape. The GNS exhibits an intense G band, a low intensity ratio of 2D to G bands (*I*_2D_/*I*_G_), and a broadened 2D band (full-width half-maxima (FWHM) ≈ 58 cm^-1^). These features are consistent with the GNS having a scrolled structure composed of several turns and with adjacent windings of the sheet being strongly coupled.

In contrast, GNSs formed by solvent intercalation between graphene and a SiO_2_ substrate showed an intense 2D band with the *I*_2D_/*I*_G_ ratio over 1^14,18,31^. For example, Li et al. reported that GNSs made from CVD-grown graphene by the solvent-assisted method lack the *π*-stacking, evidencing no changes in the Raman spectrum upon scrolling and a large interlayer distance of ≈0.39 nm between each turn of the scroll^31^. Compared with these previous results, the coupling between adjacent graphene rolls in UV tape-based GNSs is stronger, resulting in densely packed nanoscrolls owing to effective strain release and a clean interface between adjacent windings. In the previous literature, strain is not intentionally applied to graphene, so that the produced scrolls may have a less tightly wound structure.

We also found that the G band of the GNS exhibits a red shift compared to the original monolayer graphene (see Fig. 3d, f). Similar red shifts have been observed before for GNSs^14,31^, and can be explained by the strain induced by the curvature and/or residual tensile strain in the GNS, as tensile strain in monolayer graphene is known to shift the G band to lower wavenumbers^32^. Although the formation of the scroll structure via detaching from the tape surface releases the graphene’s tensile strain, we speculate that some residual strain exists in some of the GNSs. Monolayer graphene transferred on a SiO_2_ substrate generally experiences p-type doping from SiO_2_ surface having functional groups^33,34^, such as silanol groups, resulting in the upshift of the G band to relatively high wavenumbers (1587-1591 cm^-1^)^35^. On the other hand, a GNS is weakly affected by SiO_2_ surface, because the ratio of carbon atoms close to the SiO_2_ surface is very limited. Therefore, a combination of residual strain, curvature, and limited contact with the SiO_2_ surface can be attributed to the observed G-band red shift of the transferred GNRs.

As mentioned previously, the 2D band of the GNS showed significant broadening (Fig. 3e,g), reflecting the presence of strong coupling between adjacent windings. Supplementary Fig. 15 indicates that the 2D band shape of the GNS resembles that of graphite but with some differences. This likely arises from differences in stacking order, as the scrolled geometry of the GNSs inherently leads to incommensurate stacking compared with the Bernal stacking of graphite^36^. The weak D band of the GNS (Fig. 3c) indicates minimal damages from the applied strain and scrolling process. Notably, the presence of D band in GNSs has been reported previously and can originate from edge contributions or from lattice symmetry breaking induced by rolling^14,28^.

Interestingly, the Raman spectra of GNSs show sharp peaks at low wavenumbers ( ≈ 120 cm^-1^), as seen in Fig. 3h. These peaks were not observed in a monolayer graphene sheet and likely originate from the layer breathing mode (LBM), as previously reported in graphene nanoscrolls^28^. This LBM originates in the lattice vibration of graphene sheets in the out-of-plane direction, being also characteristic of multilayer graphene^36,37^. Therefore, the observation of the LBM peaks in the GNSs indicates a tightly wound structure, consistent with the observed low *I*_2D_/*I*_G_ ratio and broad 2D band (Fig. 3c, g). As inner diameters of GNSs are relatively large (Fig. 3m, n), the observed low frequency Raman peaks do not seem to originate in the radial breathing modes (RBMs) which are well known for carbon nanotubes with diameters usually smaller than 2 nm^38,39^.

We investigated the scroll formation process using a sharp probe inside a scanning electron microscope (SEM) chamber to manipulate a graphene ribbon that was not scrolled during the transfer onto SiO_2_. Fig. 3i–k displays snapshots of the in-situ manipulation of the graphene ribbon (the whole process is documented in Supplementary Movie 2). The probe was attached to one end of the ribbon and slowly lifted from the SiO_2_ surface while continuously monitoring the process by SEM. As the graphene fragment is gradually detached from the SiO_2_ surface, it starts to roll up and form a GNS. This observation indicates that the GNS can form spontaneously, suggesting that residual strain is partly preserved in transferred graphene fragments.

The cross section of a GNS was studied by a TEM to better understand its structure. The TEM image shown in Fig. 3m indicates that the GNS has a highly ordered and densely packed layered structure consisting of 15-20 graphene rolls with an inner diameter of ≈10 nm. The distance between adjacent rolls is 0.34 nm (Fig. 3n), indicating strong coupling due to the strain-induced effective scrolling and clean interface consistent with the Raman observations (see Fig. 3f–h). Although we used a solvent, such as toluene, to assist the nanoscroll formation, it is unlikely that the solvent is intercalated between rolls because the scrolling is accelerated by strong intra-layer van der Waals interaction of a graphene sheet. The inter-roll distance of ≈0.34 nm infers the absence of molecules between intra-layer space (Fig. 1g–i, 3m, n).

For a large-diameter GNS (roughly 200 nm), cross-sectional TEM images show that the scroll has collapsed (Supplementary Fig. 16a). Based on the curved multilayer edge structure seen at both sides of the collapsed GNS (Supplementary Fig. 16b), we speculate that the collapse occurred after forming a large-diameter circular scroll. The scroll cannot maintain its cylindrical structure at such large diameters, resulting in a flattened, collapsed structure. This behavior is similar to that of large-diameter carbon nanotubes, in which the collapsed structure is more stable than the cylindrical structure for diameters exceeding a critical value (≈5 nm for single-walled carbon nanotubes)^40,41^.

### Theoretical study of GNS formation

**Fig. 4:**
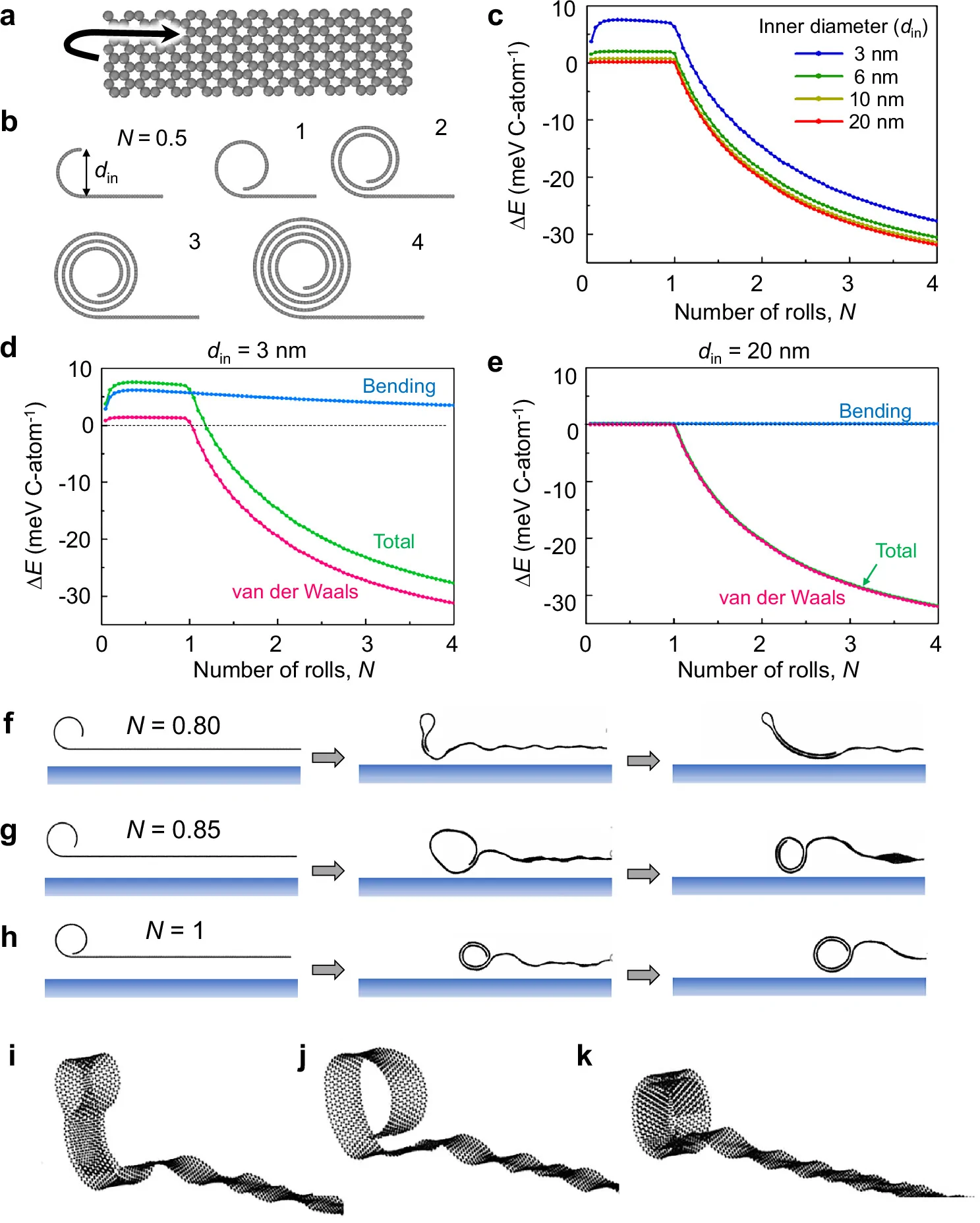
Theoretical calculations of GNS formation. **a** Structural model of a graphene sheet used for the MM calculation. **b** Structure of GNS with a different number of rolls (*N*). Here, the inner diameter (*d*_in_) was fixed to 3, 6, 10, and 20 nm. **c** Energy profiles of GNSs plotted as a function of the number of rolls for different *d*_in_. Blue, green, yellow, and red data correspond to *d*_in_ of 3, 6, 10, 20 nm, respectively. **d** Energy profiles of a small-diameter GNS (*d*_in_ = 3 nm). The energies corresponding to van der Waals interaction and the bending are shown with light blue and red curves, respectively. Total energy is shown with a green curve. **e** Energy profiles calculated for a large-diameter GNS (*d*_in_ = 20 nm). **f–h** Snapshots of MD calculation for three different initial structures. Left and right images show the initial geometry and the geometry after 100 ps, respectively. **i–k** Snapshots of the 3D structures corresponding to *N* = 0.80 (**i**), 0.85 (**j**), and 1.0 (**k**). Source data are provided as a Source Data file.

To understand the rolling mechanism from an energetical viewpoint, molecular mechanics (MM) calculations were performed on a graphene sheet (Fig. 4a). The sheet was sequentially rolled in 18° increments (i.e., 1/20 of a full turn) following Archimedean-type spiral (Fig. 4b)^42^, and the energy was calculated for every step. Fig. 4c displays the energy profiles calculated for four GNS with inner diameters (*d*_in_) ranging from 3 nm to 20 nm. The energy difference, Δ*E*, in Fig. 4c represents the difference of energy per rolled C atom with respect to the flat sheet. To simulate the experimental results, the inner diameters were fixed, so that the outer diameters increase with the number of turns (*N*) as the roll proceeds. For the small-diameter GNS with *d*_in_ = 3 nm, the total energy is positive for *N* < 1.2 but becomes negative for *N* ≥ 1.2 with monotonical decrease with increasing *N* (Fig. 4c). This means that the system is unstable at the initial stage of nanoscroll formation but progressively stabilizes when it makes a rolled structure.

To clarify the origin of the energy change, the contributions of deformation (bending) and van der Waals energies of the narrow GNS (*d*_in_ = 3 nm) are separately plotted in Fig. 4d. The plot shows that the initial instability originates in the deformation energy but van der Waals interaction between graphene turns overcomes this deformation energy at *N* ≥ 1.2. Thus, once the graphene sheet completes a single roll, it spontaneously forms multiple rolls, as the system continues gaining more van der Waals energy.

For GNSs with inner diameters larger than >6 nm, the contribution of deformation energy becomes very small. As shown in Fig. 4e, the bending energy of a GNS with *d*_in_ = 20 nm is almost negligible compared with the van der Waals energy. Therefore, GNSs tend to have relatively large inner diameters, in accordance with our experimental observations (see Fig. 3m, n and Supplementary Fig. 16).

To further understand the mechanism behind the phenomenon of rolling, we performed molecular dynamics (MD) calculations to simulate the nanoscroll formation, with different initial conditions (see Methods). Evolution of the graphene sheets with three different initial rolled structures, *N* ranging from 0.8 to 1, are displayed in Fig. 4f–h with the corresponding 3D structures in Fig. 4i–k (see Supplementary Movies 3, 4). Our calculations revealed that the formation of GNS structure is strongly dependent on the initial structure. For *N* = 0.80, folding of the graphene sheet occurs instead of making a scroll, with the top layer sliding over the bottom layer (Fig. 4f, i). For the graphene with a larger scrolled arc (*N* = 0.85), the bent ending of the graphene initially fluctuates and gradually forms a large and loosely rolled structure, finally producing a compact GNS (Fig. 4g, j). When the initial structure has a rolled-up geometry (*N* = 1.0), it smoothly forms a tightly packed, multilayer nanoscroll structure (Fig. 4h, k). In multilayer graphene, friction can drop dramatically when adjacent layers are in an incommensurate registry, as the lateral atomic forces largely cancel out, giving rise to superlubricity^43,44^. This phenomenon has been widely reported in experiments and simulations, where shear forces become negligible until the system approaches a commensurate configuration^43–45^. In our MD simulations, the innermost layers of the forming nanoscroll remain strongly incommensurate due to their high curvature and strain, allowing them to slide relative to each other with minimal resistance. This superlubric sliding manifests as a continuous reduction of the inner radius during the early stages of scrolling, helping to obtain tighter scrolls (see Supplementary Movies 3, 4).

The nanoscroll formation was also simulated at the temperature used in our actual experiments (363 K). The results are compared in Supplementary Movie 5 with those at 0 K. We found that the high temperature of 363 K induces thermal fluctuation of graphene ribbons and speeds up the scrolling as compared with that calculated at 0 K. However, there was no essential difference in the nanoscroll formation. Overall, our MD calculations suggest that it is possible to form a nanoscroll structure when an edge of the graphene sheet has a sufficient curvature and initial velocity, which was experimentally realized by our UV tape-assisted, strain engineering method.

### Heteronanoscrolls

**Fig. 5:**
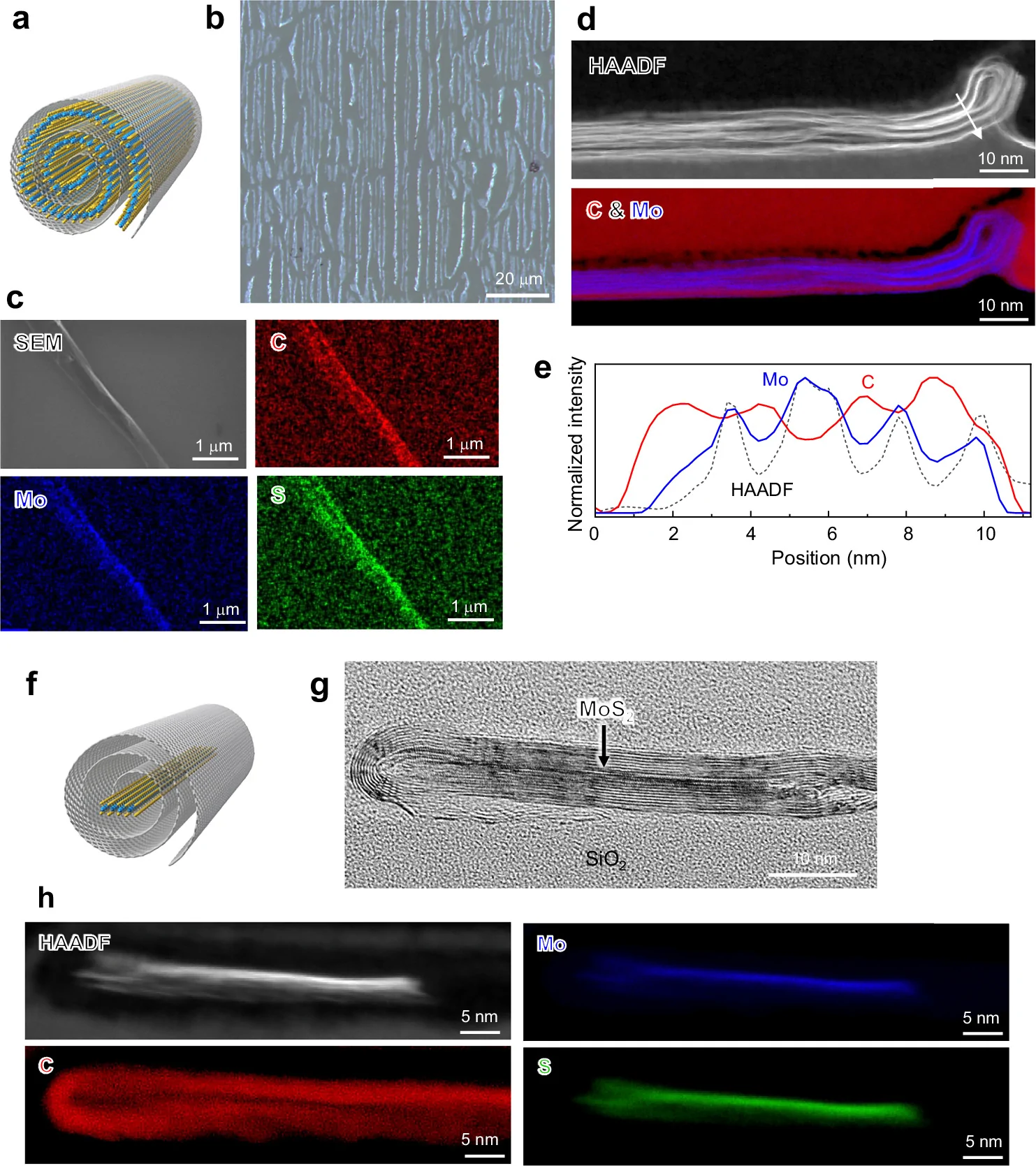
Formation of graphene-based heteronanoscrolls. **a** Structural model of MoS_2_-graphene co-nanoscroll. **b** Confocal laser microscope image of the aligned co-nanoscrolls. **c** SEM and EDX images of a co-nanoscroll. **d** Cross-sectional HAADF image and EELS elemental mapping (mixture of C and Mo) of the co-nanoscroll. **e** Line profile showing the corresponding HAADF, C, and Mo signal intensities plotted along the white arrow in the HAADF image (**d**). **f** Structural model of a MoS_2_@GNS core-shell nanoscroll. **g** Cross-sectional TEM image of the core-shell nanoscroll, in which the dark contrast corresponds to the MoS_2_ nanoribbon. **h** Cross-sectional HAADF image and EELS elemental (C, Mo, and S) mappings of the core-shell nanoscroll. The centered brighter contrast in the HAADF image corresponds to Mo and S atoms. Source data are provided as a Source Data file.

Our tape-assisted strain engineering approach can be extended to synthesize heteronanoscrolls of graphene and 2D semiconductors by leveraging the rolling of graphene to simultaneously scroll 2D materials stacked on it. To achieve this, monolayer MoS_2_ flakes grown by CVD were transferred onto a graphene/UV tape stack, followed by applying tensile strain. After releasing the strain by detaching from the UV tape, we could obtain co-scrolls of MoS_2_, and graphene as illustrated in Fig. 5a.

Fig. 5b displays the confocal laser microscope image of a dense array of MoS_2_-graphene co-scrolls. As shown in Supplementary Fig. 17, bright contrast originates in MoS_2_. Therefore, Fig. 5b indicates aligned co-nanoscrolls with a high density of 200-300 co-nanoscrolls per mm. The SEM image and the corresponding elemental mappings measured by energy dispersive X-ray spectroscopy (EDX) of a co-scroll are shown in Fig. 5c. These images confirm that both graphene and MoS_2_ are present along the longitudinal axis of the nanoscroll. The cross-sectional high-angle annular dark-field (HAADF) image and the corresponding electron energy loss spectroscopy (EELS) elemental mapping (Fig. 5d, Supplementary Fig. 18) along with the line profiles for the HAADF, C and Mo in Fig. 5e, further prove that both graphene and MoS_2_ rolled up together, realizing a heteronanoscroll with alternating graphene and MoS_2_ layers. Although the MoS_2_ monolayer is thicker than monolayer graphene, the tensile strain applied to graphene via the UV tape and the liquid intercalation between graphene and tape surface can effectively assist the formation of a nanoscroll structure. Due to the large diameters of the GNSs, this core-shell scroll showed a collapsed structure, but this method can be used to produce high-order van der Waals superlattices consisting of alternating 2D materials^46^.

We could also produce core-shell heteronanoscrolls by transferring MoS_2_ nanoribbons on graphene, followed by the UV tape-assisted nanoscroll formation. The MoS_2_ nanoribbons were grown on a-plane sapphire^47^, and transferred onto a graphene/UV tape stack for the successive strain application. Fig. 5f, g shows an atomic model and the cross-sectional TEM image of the MoS_2_@GNS core-shell heterostructure, respectively. The HAADF image and corresponding EELS elemental mapping for C, Mo, and S atoms (Fig. 5h) clearly show the presence of a MoS_2_ nanoribbon inside the scroll core.

A core-shell structure was also realized by using strained graphene and molecules. A vapor of metal chloride molecules (AlCl_3_ and CuCl_2_) was introduced while heating the strained (but not scrolled) graphene on a SiO_2_ substrate. Due to the remaining strain in graphene, the molecular deposition at high temperature initiated the formation of a GNS with the molecules inside its core. The TEM image and the corresponding elemental mappings revealed the presence of a metal chloride cluster surrounded by graphene layers (Supplementary Fig. 19), indicating the formation of a metal chloride@GNS core-shell heterostructure. The stored metal chloride molecules can be released in aqueous solution so that this might be used to store and/or deliver specific molecules.

### Electrical characterization and device applications

**Fig. 6:**
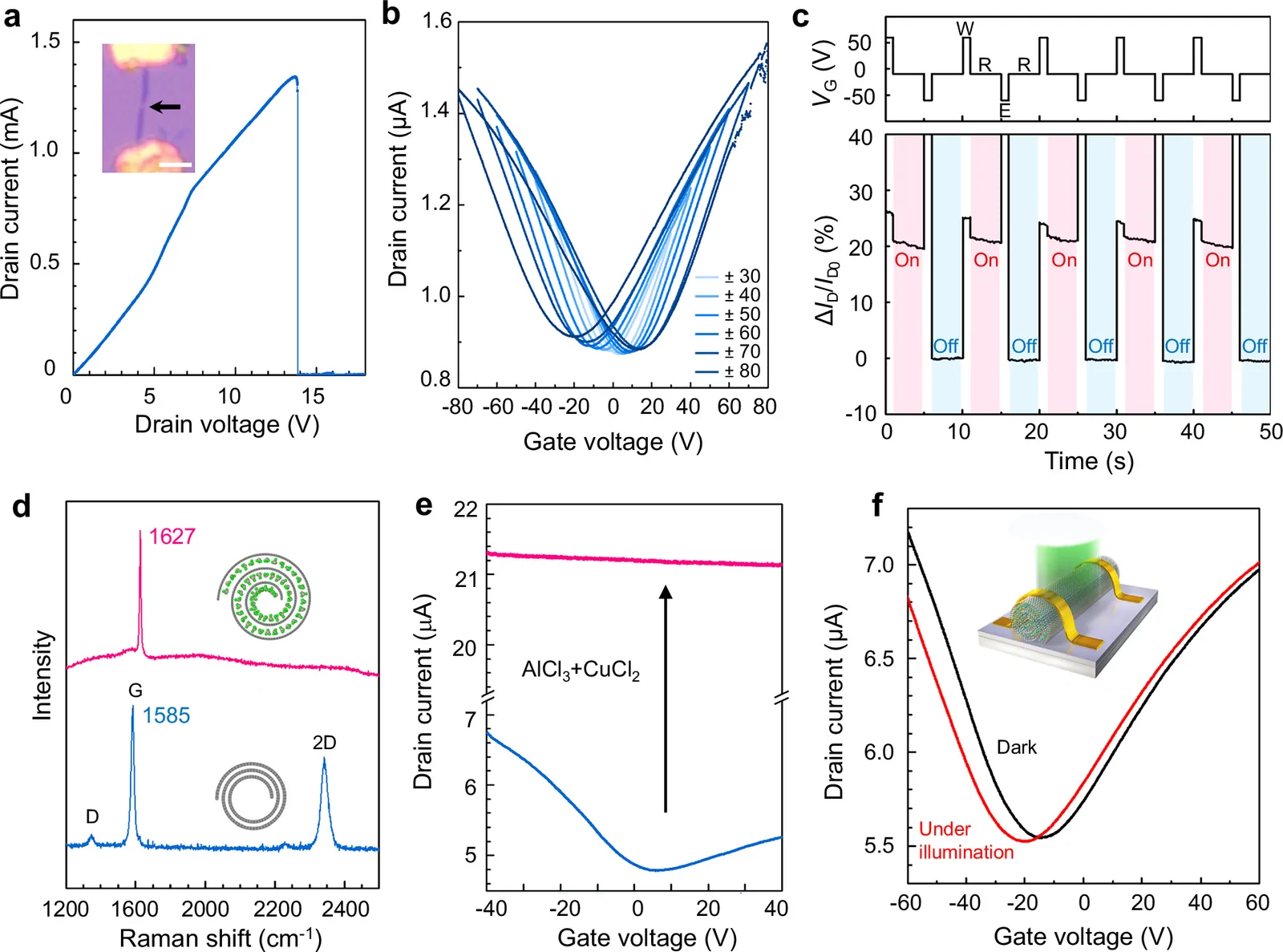
Electrical transport properties and applications of GNSs, intercalated GNS, and heteronanoscrolls. **a** Breakdown behavior of a GNS channel. The inset shows the optical image after the breakdown (scale bar: 2 μm). The arrow indicates the position where the breakdown occurred. **b** Transfer curves of a GNS-FET measured at six different sweep gate voltages (from ±80 to ±30 V with a 10 V step) in vacuum. **c** Memory effect of a GNS-FET. The upper panel shows the profile of the applied gate voltage, and the lower panel shows the change in drain current, revealing the memory effect due to the large hysteresis. **d** Raman spectra of a pristine GNS (blue) and an AlCl_3_-CuCl_2_ intercalated GNS (pink). Insets show the schematic models. **e** Transfer curves of a GNS measured before (blue) and after the intercalation (pink). **f** Light response of the co-nanoscroll of MoS_2_ and graphene, showing a light-induced shift of the transfer characteristics toward negative gate voltages. Inset is a schematic of a co-nanoscroll device under illumination. Source data are provided as a Source Data file.

The electrical current sustained by a GNS can exceed that of a planar graphene sheet for the same channel geometries, because the whole rolled graphene sheet can contribute to carrying the current. Fig. 6a displays the breakdown behavior of a GNS-FET, showing an almost linear increase in the current that evidence that the current flows through the whole scrolled graphene^14–18^. The device stands a high drain voltage up to ≈14 V and combined with the AFM measurement (Supplementary Fig. 20), the maximum current density was estimated to be 2.3×10^8 ^A cm^-2^ for the GNS with a diameter of 27 nm. This value is higher than the previously reported values for a GNS (5 × 10^7 ^A cm^-2^)^14^ and two orders of magnitude higher than that of Cu metal (10^6 ^A cm^-2^). In the case of MWCNTs, the layer-by-layer breakdown from outer walls induces the step-like oxidation against the drain voltage^19^. However, the abrupt decrease in the current in our GNS device suggests that the whole graphene layer contributes to the current flow.

We further investigated the carrier transport properties of GNSs by fabricating bottom-gated field-effect transistors (FETs). Fig. 6b shows transfer curves of the GNS-FET device measured in vacuum for different sweep ranges of the gate voltage. The GNS-FET exhibited a clear hysteresis that increases with the sweep gate voltage. The hysteresis was almost independent of the applied drain voltage, as shown in Supplementary Fig. 21, which is consistent with gate-controlled charge trapping processes. Together with the negligible hysteresis in the non-scrolled graphene FET (Supplementary Fig. 22), this suggests that the dominant charge trapping is associated with the structure of the GNSs, likely due to water and/or oxygen molecules trapped within the spaces between turns of the scroll. This result is consistent with the previous study reporting large hysteresis in MoS_2_ nanoscroll devices, implying that the nanoscroll structure is suitable to store charges owing to the open and flexible structure^48^.

The substantial hysteresis observed in the GNS devices can be exploited for nanoscale memory applications. Fig. 6c shows the change in drain current measured while the gate voltage was varied sequentially. Here, the write (W) and erase (E) pulses were set 60 and -60 V, respectively, while read voltage (R) was -10 V. The high-resistance state (HRS) and low-resistance state (LRS) can be stably maintained by input gate voltage. Therefore, the large and reproducible hysteresis in the GNS device enabled a repeatable memory effect. Further optimization of the trapped species within the GNS may provide a route to enhancing the memory performance of these devices.

By utilizing the expandable nanoscroll structure, we intercalated metal chloride molecules (AlCl_3_ and CuCl_2_) to GNSs, which act as p-type intercalant for graphite and bilayer graphene^49,50^. As shown in Fig. 6d, Raman measurements demonstrated that intercalation successfully occurred for GNSs, evidenced by the large blueshift of the graphene Raman G band. In addition, the 2D band was strongly suppressed, a feature characteristic of stage-1 GICs and few-layer graphene intercalated with metal chloride (see Supplementary Fig. 23). In the case of bulk graphite, the intercalation of metal chlorides increases the interlayer distance from 3.35 Å to 9–10 Å^49^, leading to the decoupling of adjacent graphene layers. This can also occur to the intercalated GNSs, partly decoupling the van der Waals interaction between neighboring rolls. At the same time, due to hole doping from the metal chlorides to the GNS, its electrical conductivity dramatically increased, as shown in Fig. 6e. Notably, this conductivity enhancement persists even after exposure of the intercalated sample to air, indicating that metal chlorides remain confined between the scroll rolls and/or within the core. The intercalation in GNS is not limited to p-type metal chloride molecules. We observed n-type doping by the intercalation of K ions in GNSs, as displayed in Supplementary Fig. 24.

The MoS_2_-graphene heteronanoscrolls can be potentially used as a photosensor, combining the high optical absorbance of MoS_2_ and the high carrier mobility of graphene, with the scroll geometry also expected to enhance light absorption^51,52^. Thus, we fabricated FETs using co-scrolls of graphene and MoS_2_, as illustrated in Fig. 6f inset. The co-scroll FET exhibited clear photo-switching behavior, with a substantial shift of the transfer characteristics toward negative gate voltage upon visible light illumination (532 nm wavelength), corresponding to a decrease in the drain current in the hole-doped regime (Fig. 6f). On the other hand, the graphene-FET did not exhibit such light-induced current modulation (Supplementary Fig. 25). The observed light-induced current decrease in the GNS-FET can be explained by the photo-induced electron transfer from MoS_2_ to graphene, as reported previously for a vertically stacked MoS_2_/graphene heterostructure^35^. The modulation of the drain current ( ≈ 5%) is larger than the previously reported heterostructure ( ≈ 2%)^35^, owing to the scroll structure having a larger cross-section against the illuminated light. Stable optical response was observed over repeated measurement cycles (Supplementary Fig. 26). Overall, the nanoscroll structure of graphene and its heterostructures, including co-scroll and core-shell scrolls, offer a platform to derive electronic and optoelectronic applications.

## Discussion

Large-scale strain-engineered synthesis of GNSs was demonstrated using UV tape specially designed for the transfer of graphene. Tensile strain in graphene induced by the extended UV tape was used to produce highly aligned GNSs, via releasing the strain with external stimuli. Compared with previously reported methods^14,18,28^, this approach yields a more than four-fold increase in nanoscroll density, and more importantly allows the control of the scroll orientation and chirality, by changing the direction of extension. Atomistic calculations revealed that the formation of the nanoscroll is governed by the initial curvature of the graphene edge and that, once initiated, scrolling becomes energetically favorable as the van der Waals interaction energy overcomes the deformation energy, thereby driving further scrolling. The GNS transistors exhibited clear hysteresis, which was applied to a memory device. Owing to their open and flexible structure, GNS allowed intercalation of metal chlorides and alkaline metals, leading to a substantial increase in the electrical conductivity. We also demonstrate the controlled synthesis of MoS_2_-graphene heteronanoscrolls, including co-scrolls and core-shell structures, offering a viable route toward engineered 1D superlattice structures. The heteronanoscrolls exhibited clear optical response due to a combined effect of the high optical absorbance of MoS_2_ and high mobility of graphene. Our UV tape-based approach provides a practical route for the large-scale mechanical assembly and processing of atomically thin 2D materials.

## Methods

### Syntheses of 2D materials

Monolayer graphene was grown on Cu(111)/c-plane sapphire (α-Al_2_O_3_ (0001), Kyocera Co.) by ambient-pressure CVD. The Cu(111) thin film (1 μm-thick) was deposited by radio-frequency sputtering (Shibaura Mechatronics CFS-4EP), and the graphene was grown by flowing CH_4_, H_2_ and Ar at 1075-1080 °C^30,53^. MoS_2_ grains and nanoribbons were synthesized using MoO_3_ and S powder on *c*-plane and *a*-plane sapphire, respectively. MoS_2_ grains were grown at 900-950 °C^54^, while nanoribbons were grown at a higher temperature (1100 °C)^47^.

### GNS synthesis

We used the UV tape specially designed for monolayer graphene transfer. The chemical composition and preparation method of the UV tape are reported in ref. ^29^. First, we adhered the UV tape onto as-grown graphene and illuminated with a UV-light emitting diode (LED) lamp (Quark Technology Co. Ltd., Japan) with 365 nm wavelength to harden the adhesive layer, which reduced the adhesion force to graphene. The UV-LED illumination condition was ≈93 mW cm^-2^ for 11 s (the total energy is ≈1000 mJ cm^-2^). Upon this illumination, the adhesion force of the UV tape decreased from 1.2 to 0.15 N for a 10 mm tape width against Si wafers^29^. The electrochemical delamination of the tape/graphene stack from the Cu surface was performed in NaOH aqueous solution, followed by washing with deionized water. Then, the tape/graphene was fixed to mechanical clamps, and extended up to 15% and kept for 60 min (the experimental setup is shown in Supplementary Fig. 1).

After releasing the strain, excess stimulus was applied to the graphene/tape stack to detach graphene from the tape surface, which results in the GNS formation. This was done by either heating on a hotplate at 90 °C, heating with microwave (700 W, 8 min), dropping organic solvent (toluene, acetone, and chloroform), or pressing onto a SiO_2_ substrate. We found that the combination of the hotplate heating and the drop of toluene is the most effective to make high-density GNSs. Here, we dropped only a small amount of solvent onto the adhesion layer to avoid swelling of the underlying support layer (polyolefin). Although the microwave treatment can also produce high-density GNSs, the tape partly melted, making it difficult to transfer to the final SiO_2_ substrate. Finally, the GNSs/tape was attached to a target SiO_2_/Si substrate and slowly removed the tape for further characterizations. This significant reduction of the adhesion force by the UV light allowed to leave GNSs on a SiO_2_/Si substrate.

### Heteronanoscroll synthesis

The graphene/tape stack was attached to as-grown MoS_2_ on sapphire (either a MoS_2_ film^54^ or a MoS_2_ nanoribbon array^47^), followed by delaminating MoS_2_/graphene/tape stack from the sapphire with the aid of deionized water or aqueous KOH solution. Subsequently, this stack was mounted on the tensile jig, and heteronanoscrolls were fabricated by following the same procedure used for GNS synthesis.

### Intercalation

GNSs on a SiO_2_/Si substrate and AlCl_3_ and CuCl_2_ powders were sealed together in vacuum using a Pyrex tube. For the intercalation the Pyrex tube was heated at 160 °C and kept at the same temperature. The intercalation of K ions was performed at 200 °C for 60 h. We also enclosed PGS graphite sheet (EYGS, Panasonic Co.) to monitor the progress of the intercalation. A photograph of the tubes enclosing GNSs, intercalant, and PGS is presented in Supplementary Fig. 27. Raman spectra were measured for a sample inside the tube without air exposure to air to avoid degradation.

### Characterizations

Optical and confocal laser microscope images were measured with Keyence VHX-7000 and Lasertech OPTELIX, respectively. SEM and EDX were measured with HITACHI S-4800 equipped with BRUKER FlatQUAD, and AFM images were collected using Bruker Nanoscope V in ScanAsyst mode. In-situ SEM was measured with FEI Versa 3D DualBeam using a W probe^55^_._ Raman and PL spectra were collected with Tokyo Instruments Nanofinder 30 using a 532 nm laser excitation with a spot size of ≈0.6 μm. No processing of the Raman and PL was applied. In figures with stacked plots, the spectra are displaced vertically by a constant intensity offset for clarity. The cross-section of GNS and heteronanoscrolls were fabricated by a focused ion beam (FIB) with Hitachi High-Tech NX-2000. Cross-sectional TEM and STEM-HAADF images were collected using JEOL ARM200F equipped with a CEOS probe-corrector, while EELS was measured using the Gatan Continuum K3 system operated at 200 kV. For the top-view measurements, the as-prepared GNS samples were first transferred onto TEM grids (Quantifoil). Then the high resolution (HR)-TEM and HR-STEM observations were carried out using Triple-C#2 and Triple-C#3 (JEOL, ARM200F-based microscopes equipped with delta corrector, operating at 60 kV), respectively. For the fabrication of FETs with a GNS or a heteronanoscroll channel, we used photolithography to make the pattern for metal electrode deposition. Doped silicon substrates with 300 nm top SiO_2_ layer were used. We deposited two types of metal electrodes (Au (30 nm)/Ni (10 nm) and Au (30 nm) only) by electron beam evaporation, followed by lift-off with acetone. The FETs were measured using a semiconductor analyzer (Keysight B1500A) in vacuum ( < 5×10^-4 ^Pa) at room temperature. Devices were annealed in a vacuum at 200 °C prior to the measurement. Photoresponse was measured by illuminating a device with a 532 nm laser, and the power densities were controlled by using optical filters. The typical power of the illuminating laser was 1.3 W m^-2^. The details of this optical measurement are reported in ref. ^35^.

### Computational methods

MD simulations of the GNS were performed using the LAMMPS package^56^. As illustrated in Supplementary Fig. 28, the initial GNS configuration was constructed by rolling one end of a finite graphene ribbon with zigzag edges (length *L* = 204.6 nm; width *W* = 1.99 nm, containing 16,629 carbon atoms, without a periodic condition) into an Archimedean spiral on a substrate (0.34 nm below the graphene sheet), defined by Eq. (1):

1 $$r(\phi )={r}_{{{\rm{in}}}}+(\frac{\delta }{2{{\rm{\pi }}}})\phi,$$

where *r* and *ϕ* denote the radial distance from the scroll axis and the azimuthal angle, respectively, and parameters *r*_in_ = 1.5 nm and *δ* ≈ 0.34 nm correspond to the inner radius and the interlayer spacing, respectively. To simulate the scrolling dynamics, tangential velocities corresponding to an angular velocity *ω*_0_ = 0.6 rad s^-1^ were assigned to the carbon atoms in the scrolled region (indicated by the red arrows in Supplementary Fig. 28). The dimensions of the simulation cell were chosen to be sufficiently larger than the graphene ribbon to avoid spurious interactions due to periodic boundaries. Interatomic forces were modeled using an adaptive intermolecular reactive empirical bond order (AIREBO) potential^57^ for C–C interactions and a continuum Lennard–Jones (12–6) wall potential^58^ for interactions between the graphene and the substrate. This AIREBO potential is widely used for calculations of carbon materials, including graphene. For the wall potential, the parameters were set to *ε* = 0.003 eV and *σ* = 0.34 nm, and a cutoff distance of *r*_c_ = 0.85 nm. MD simulations were performed for different environment temperatures, 0 K and 363 K (Supplementary Movie 5 compares the calculation results). The equations of motion were integrated in the microcanonical (*NVE*) ensemble using the velocity-Verlet algorithm with a time step of 0.2 fs. To model anchoring to the substrate, the carbon atoms at the end opposite to the scrolling side (marked by a red dot in Supplementary Fig. 28) were constrained such that their motion parallel to the substrate (*x-y* plane) is fixed, while motion perpendicular to the substrate (*z* direction) is allowed.

To evaluate the intrinsic energetics of GNS structures, we performed MM calculations using the same atomic models described above. The MM calculations were designed to study the static potential energy landscape of GNSs and, therefore, did not involve time evolution or structural relaxation. The stability of a GNS is governed by the competition between the bending energy and the van der Waals interaction energy in the total potential energy, primarily represented in the AIREBO framework by the covalent interaction term and the Lennard-Jones term, respectively. The total potential energy of the GNS was calculated as a function of the azimuthal angle *ϕ* for different values of *r*_in_. The energetic stability of a GNS was assessed by comparing its potential energy with that of the corresponding flat graphene nanoribbon.

### Reporting summary

Further information on research design is available in the Nature Portfolio Reporting Summary linked to this article.

## Supplementary information

supplementary Information file

Description of Additional Supplementary Files

Supplementary Movie 1

Supplementary Movie 2

Supplementary Movie 3

Supplementary Movie 4

Supplementary Movie 5

Reporting Summary

Transparent Peer Review file

## Source data

Source Data new

## Data Availability

The data that support the findings of this study are available from the corresponding author upon request. Source data are provided as a Source Data file. Source data are provided with this paper.
