# Supplementary material for: Strain-engineered fabrication of high-density aligned graphene-based nanoscrolls via UV-tunable adhesive tapes
Source: Nat Commun. 2026 Oct 21;17:10647. doi: 10.1038/s41467-026-78264-8 (PMC13687067; doi:10.1038/s41467-026-78264-8)
Supplement: Supplementary file 1 [file 41467_2026_78264_MOESM1_ESM.pdf]

Supplementary Information

UV tape-assisted synthesis of high-density, aligned graphene nanoscrolls and heteronanoscrolls

Maki Nakatani[1], Akari Sudo,[2] Pablo Solís-Fernández,[1] Haiming Sun,[3] Satoru Fukamachi,[1] Tomoki Maezawa,[4] Tatsuya Ikuta,[5] Takako Matsunaga,[1] Aika Uchida,[1] Masatoshi Mitsuhara,[1,4] Yung-Chang Lin,[3,6] Koji Takahashi,[5] Rika Matsumoto,[7] Kazu Suenaga,[3] Takahiro Yamamoto[2] & Hiroki Ago[1,4,8*]

[1] Faculty of Engineering Sciences, Kyushu University, Kasuga, Fukuoka 816-8580, Japan

[2] Department of Physics, Tokyo University of Science, Kagurazaka, Shinjuku, Tokyo 162-8602, Japan

[3] SANKEN (The Institute of Scientific and Industrial Research), The University of Osaka, Osaka 567-0047, Japan

[4] Interdisciplinary Graduate School of Engineering Sciences, Kyushu University, Kasuga, Fukuoka 816-8580, Japan

[5] Department of Aeronautics and Astronautics, Kyushu University, Fukuoka 819-0395, Japan

[6] Nanomaterials Research Institute, National Institute of Advanced Industrial Science and Technology (AIST), Tsukuba 305-8565, Japan

[7] Faculty of Engineering, Tokyo Polytechnic University, Atsugi, Kanagawa 243-0297, Japan

[8] Center for Semiconductor and Device Ecosystem (CSeDE), Kyushu University, Kasuga, Fukuoka 816-8580, Japan

[*]Corresponding author: Hiroki Ago (ago.hiroki.974@m.kyushu-u.ac.jp)

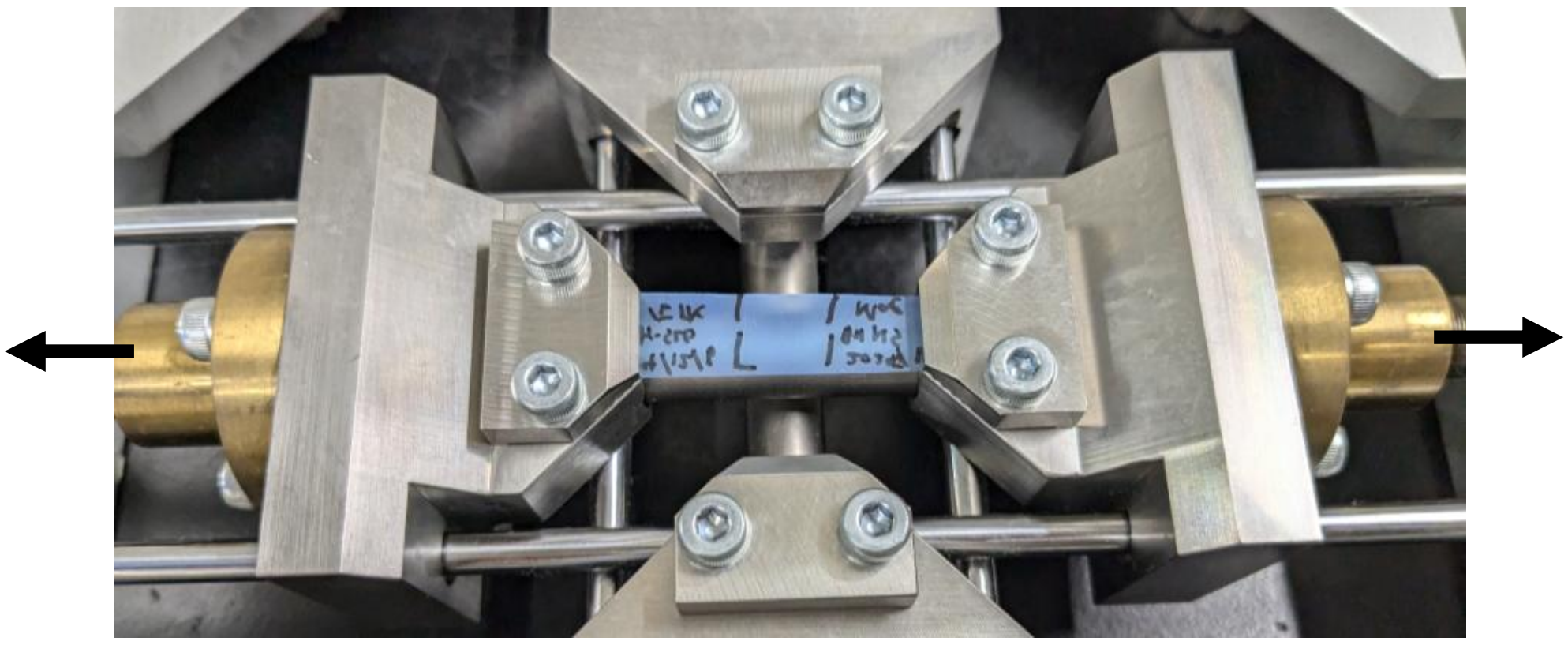

Supplementary Fig. 1. Experimental setup for applying tensile strain to graphene/UV tape stack. Photograph of extending the graphene/tape stack with mechanical jig. The stack was fixed with two metal clamps and extended to the directions indicated by the arrows. The marked central square area indicates the position of a transferred graphene sheet (typically 1 × 1 cm^2).

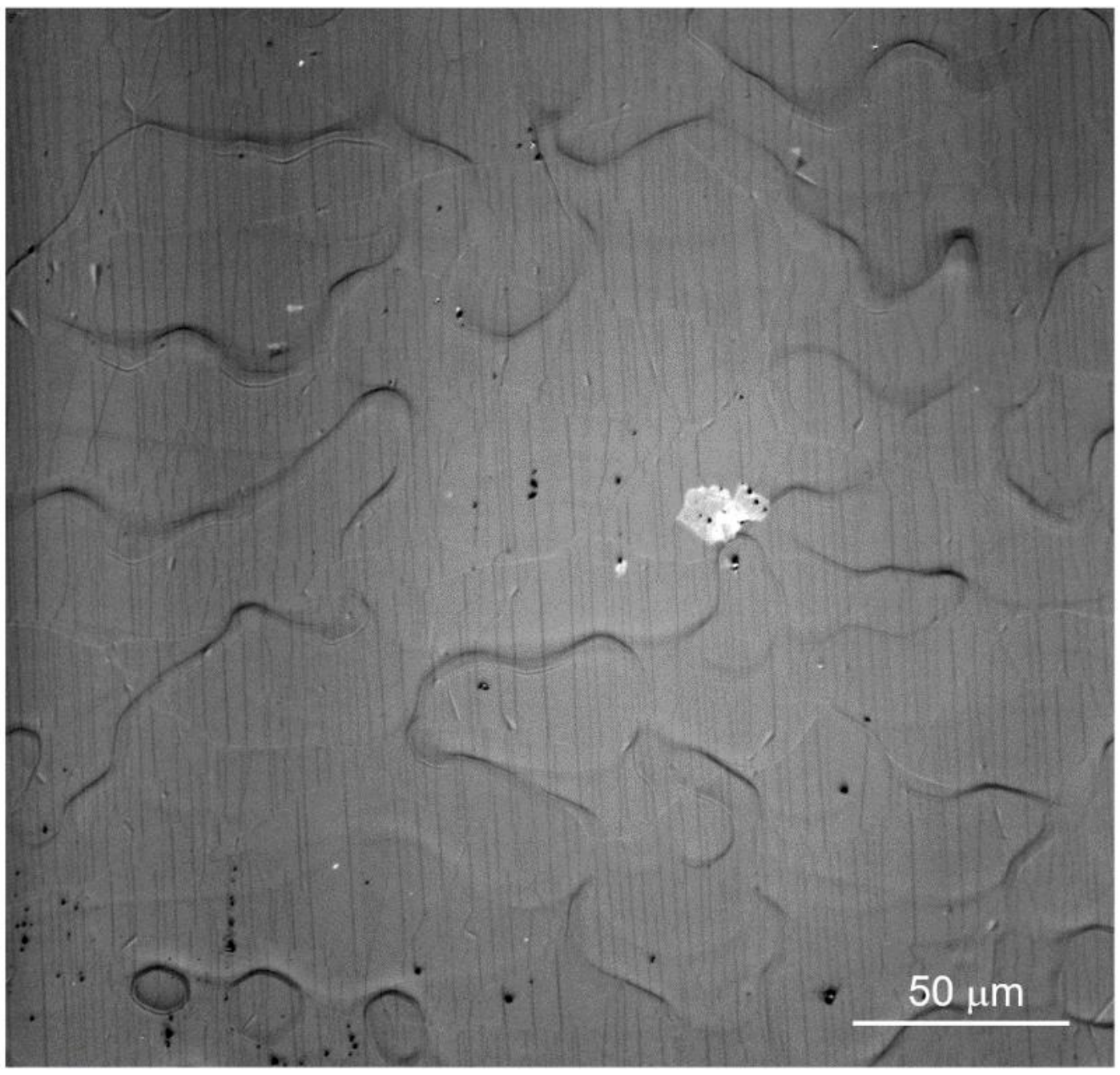

Supplementary Fig. 2. Cracks in graphene induced by extending the graphene/tape stack. Confocal laser microscope image of monolayer graphene measured during applying 7.5% tensile strain. The curved morphology is originated in the adhesive layer.

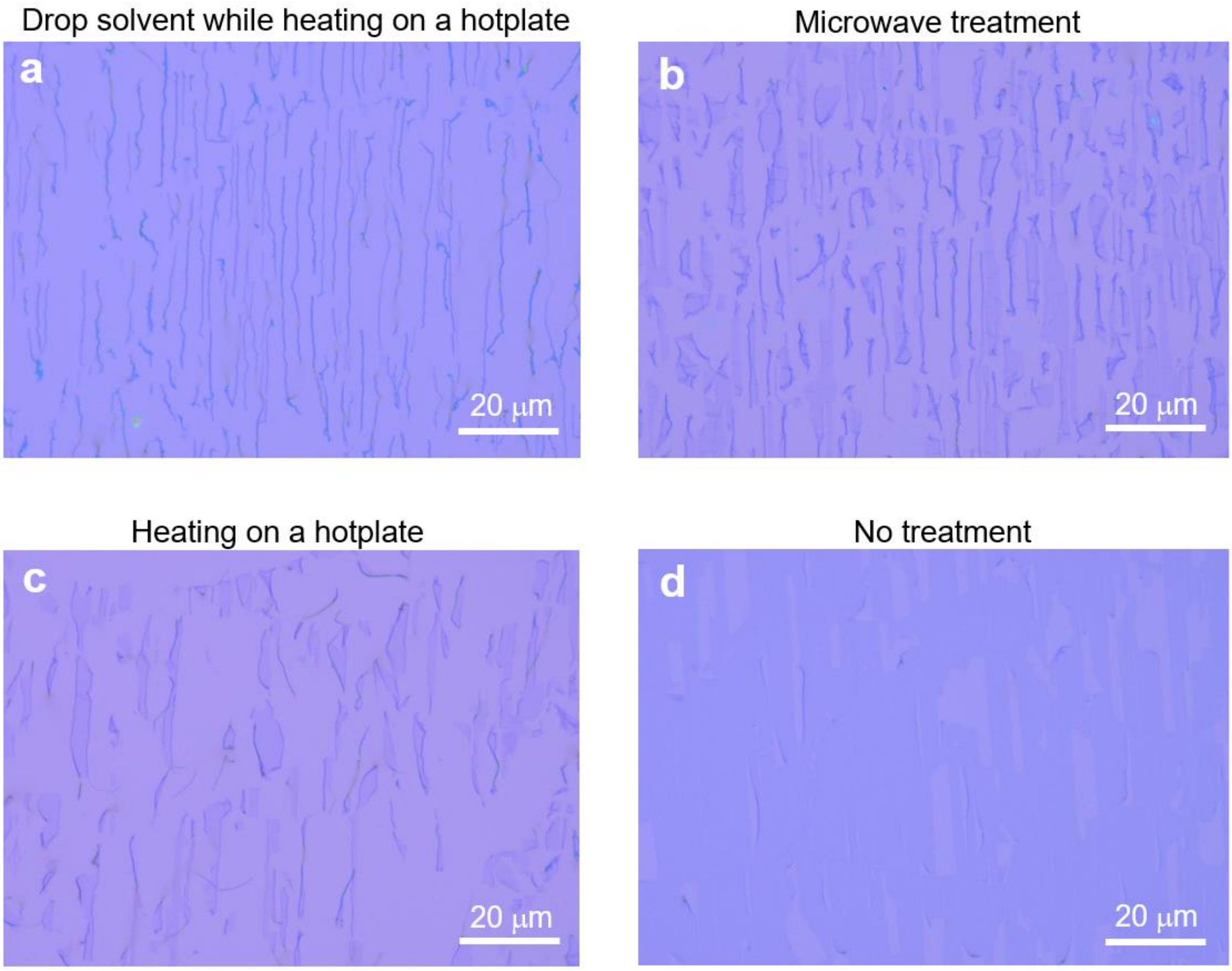

Supplementary Fig. 3. Effect of stimulus on the detachment of graphene from the stretched UV tape. Optical images of transferred GNSs on SiO_2 substrates. **a,** To synthesize GNSs, organic solvent (toluene) was dropped on the graphene/UV tape stack, while heating on a hotplate. **b,** The stack was subjected to microwave-assisted heat treatment. **c,** The stack was subjected to heat treatment on a hotplate. **d,** The stretched graphene was transferred on SiO_2 without any treatment.

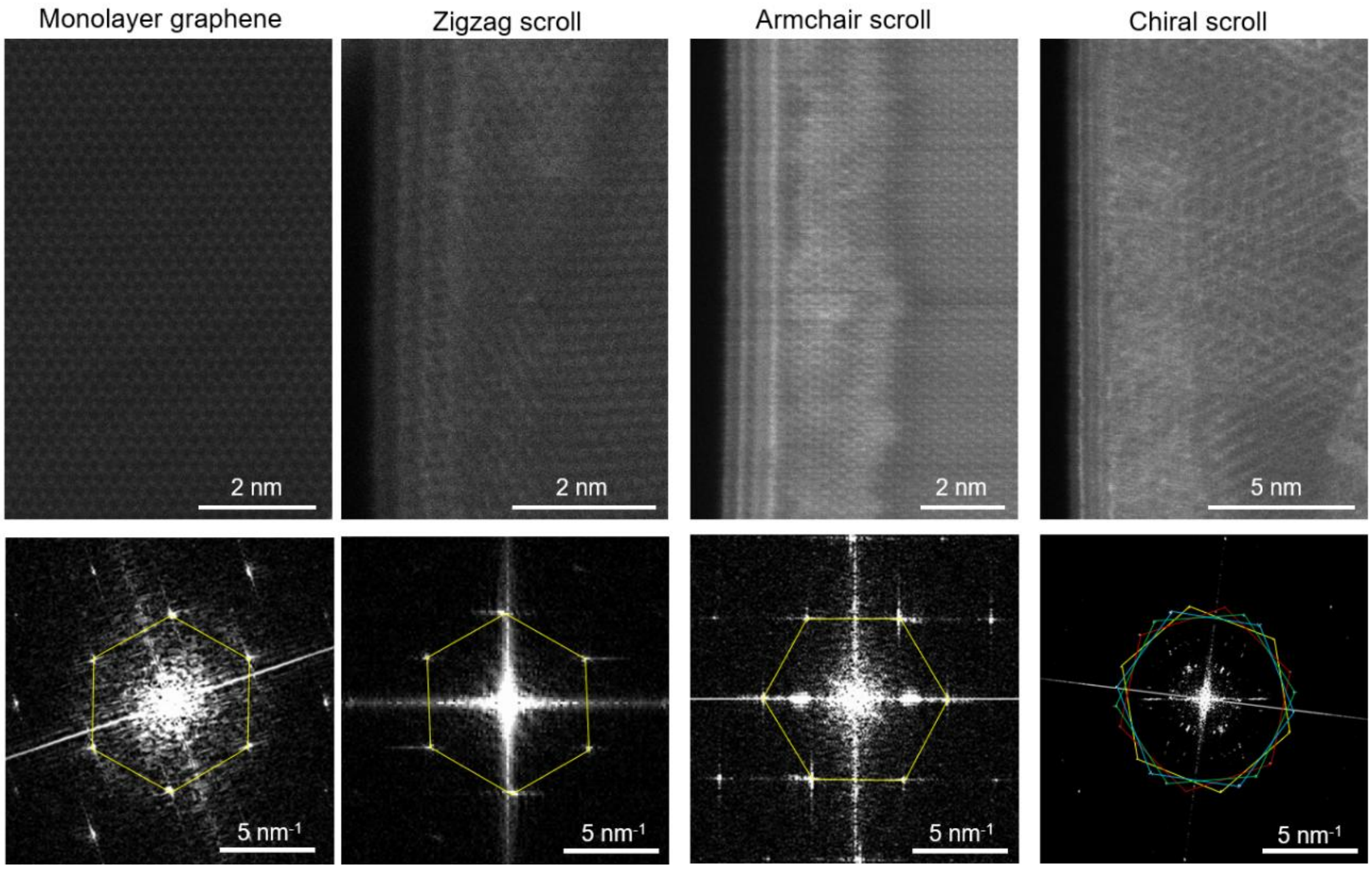

Supplementary Fig. 4. High-resolution STEM images of monolayer graphene and GNSs rolled with controlled chirality. Upper panels show the STEM images, and lower panels show the corresponding FFT patterns.

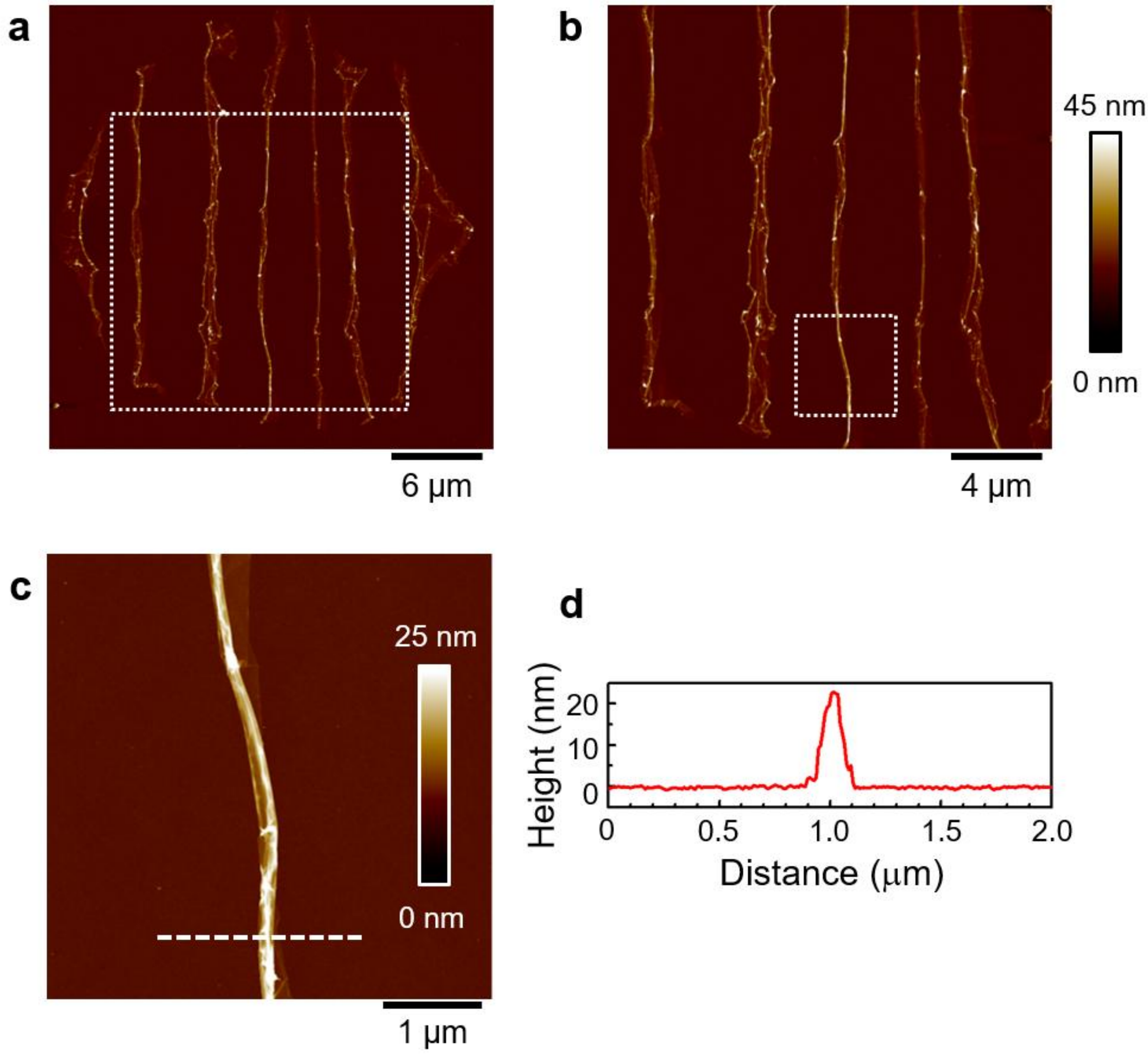

Supplementary Fig. 5. AFM characterization of aligned GNSs formed from a hexagonal graphene grain. **a-c**, AFM images of aligned GNSs formed from a hexagonal graphene with different magnifications. **d**, Height profile of the GNS marked in **c**.

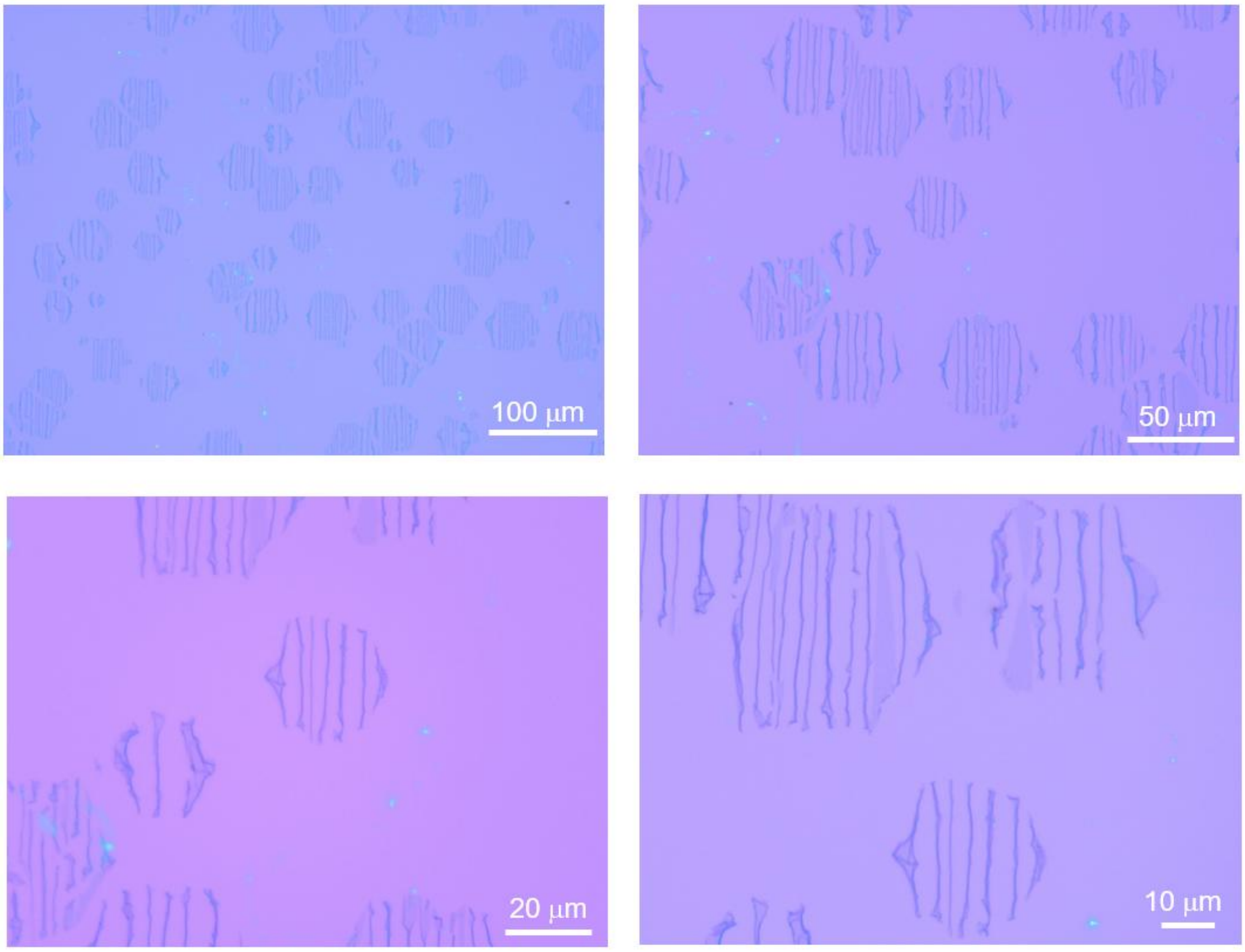

Supplementary Fig. 6. Optical images of large-area, aligned zigzag GNSs. These optical images show an identical sample with different magnifications. Tape residues (appeared as light blue) are partly observed together with the aligned GNSs. This is because higher temperature (90 °C) was required to detach the graphene sheet from the tape surface to make nanoscroll structure, as compared with the standard UV tape transfer process used for a graphene sheet (typically 80 °C). The adhesive residue appeared as blue contrast can be removed by soaking in acetone and the successive H_2 annealing.

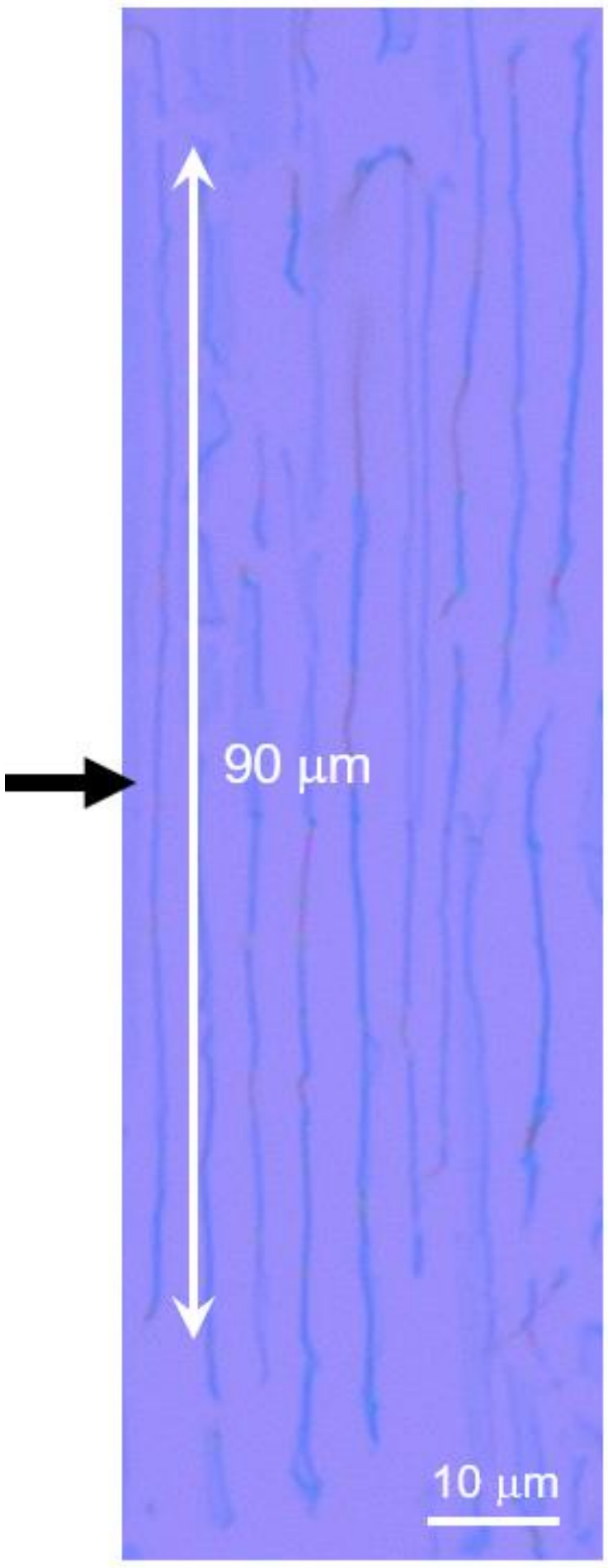

Supplementary Fig. 7. Long GNS achieved with the UV tape. Optical image of a 90 μm-long GNS (indicated by the black arrow).

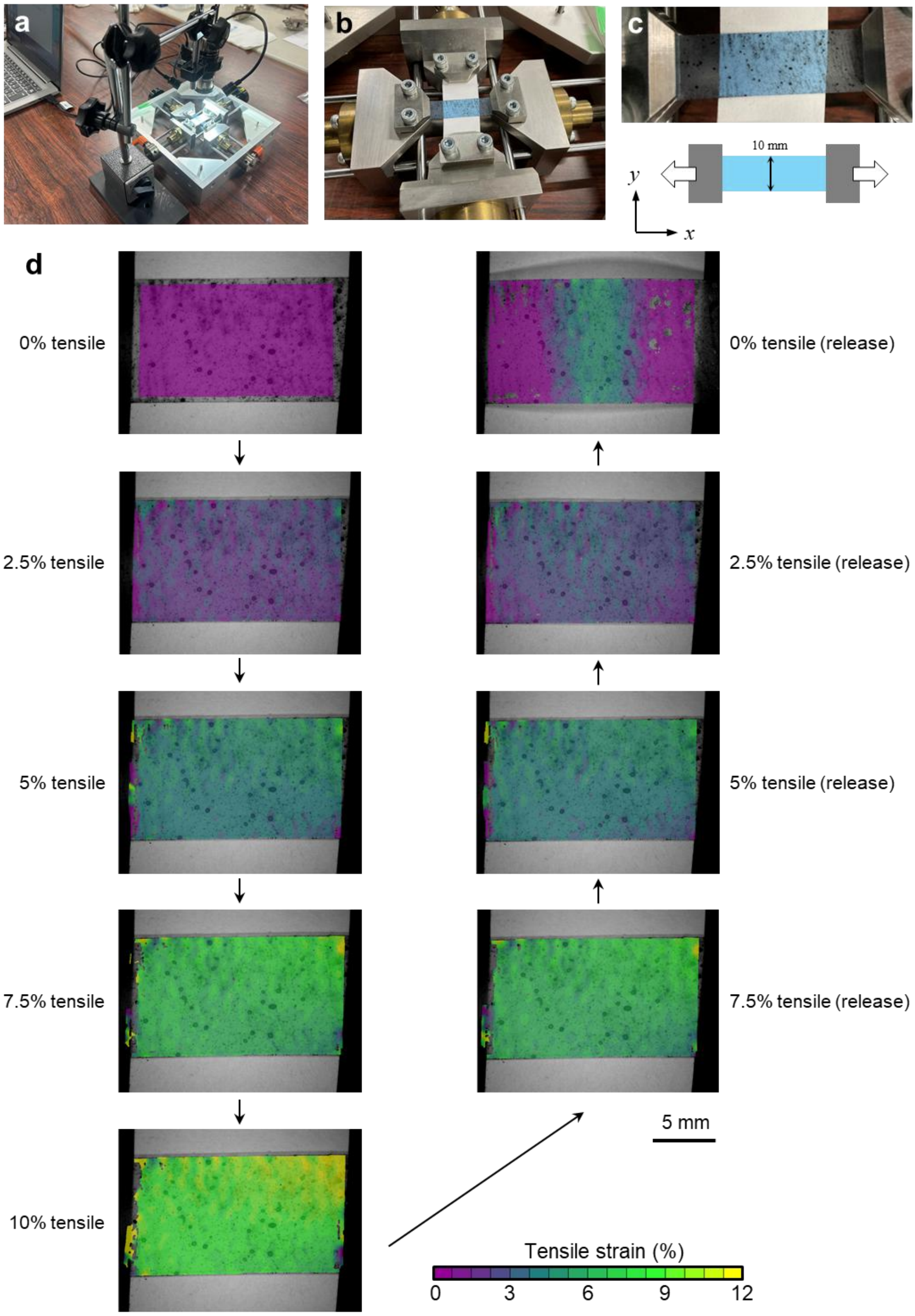

Supplementary Fig. 8. Experimental measurement of tensile strain applied in the UV tape measured by a digital image correlation method. a,b, Experimental setup used to measure the spatial distribution of applied strain. **c**, On the graphene/UV tape surface, a number of small black particles were widely deposited, which were used to monitor the change of each particle

position with respect to the applied strain using the mechanical jig. The stack was stretched along *x*-direction in the lower panel, while following the positions of particles. **d,** Evolution of tensile strain in the UV tape. We stretched the UV tape up to 10%, and then returned to the original position (0%). These images confirm that the applied tensile strain closely follows the calculated value from the extended length of the UV tape. However, when the tensile was increased to ~10%, inhomogeneities were observed due to the deformation of the UV tape. Note that applying strain up to 10% induces plastic deformation in the tape, resulting in the buckling after fully releasing the strain. Thus, the strain distribution shown in "0% tensile (release)" does not represent the actual remaining strain. The continuous images of the strain distribution can be found in Supplementary Movie 1.

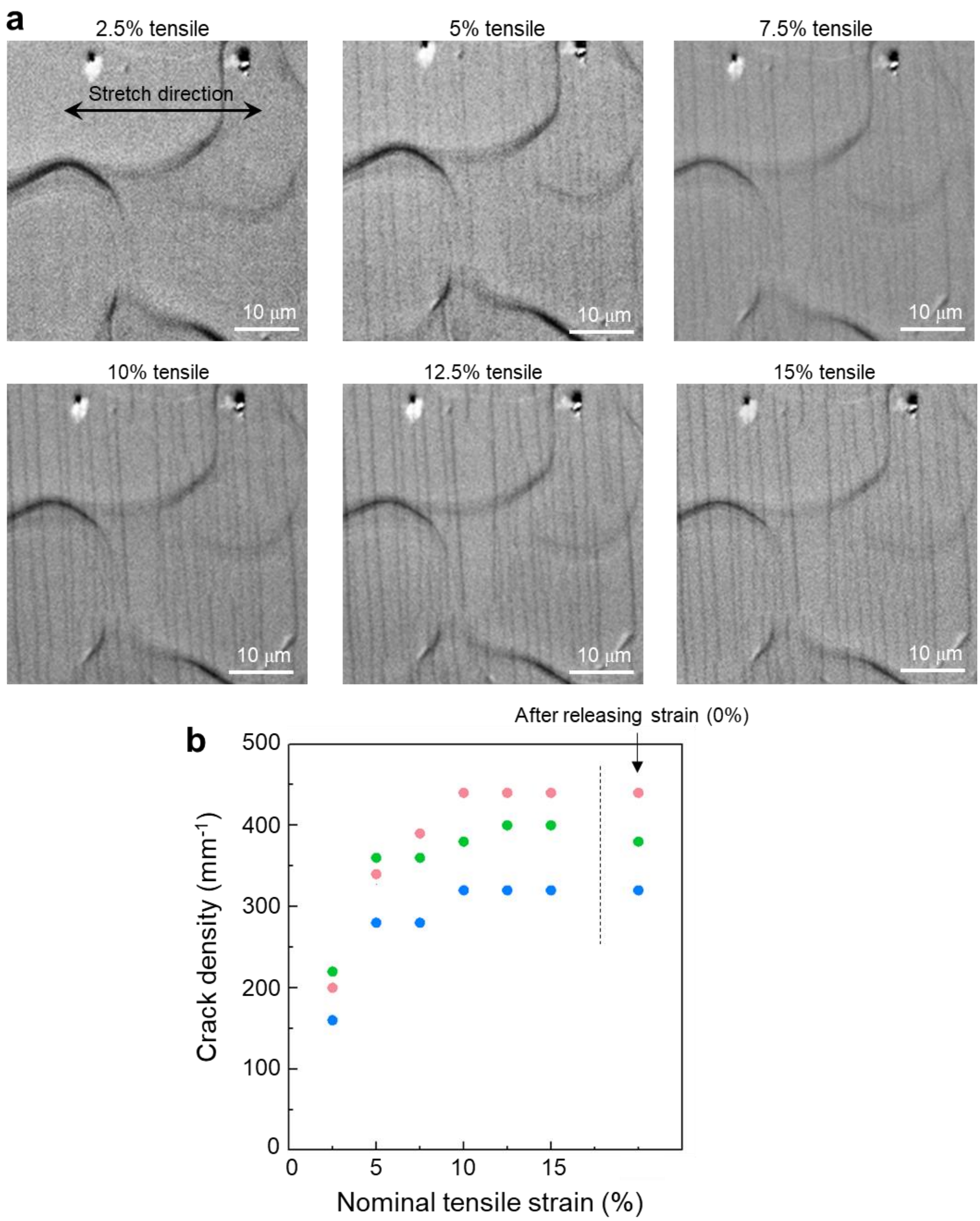

Supplementary Fig. 9. Crack formation while applying strain to the UV tape. a, Confocal laser microscope images of monolayer graphene on the UV tape captured under different applied strains (the strain values are the nominal values calculated from the change of the tape length). To monitor the crack formation, we captured images at identical positions. It is clearly seen that cracks are formed by extending the UV tape, with the number of cracks increasing with strain. **b**, Crack density plotted against the applied tensile strain. The plot shows three different positions of the stack, indicating a similar trend regardless of the area. The crack density practically saturates at ~10% strain.

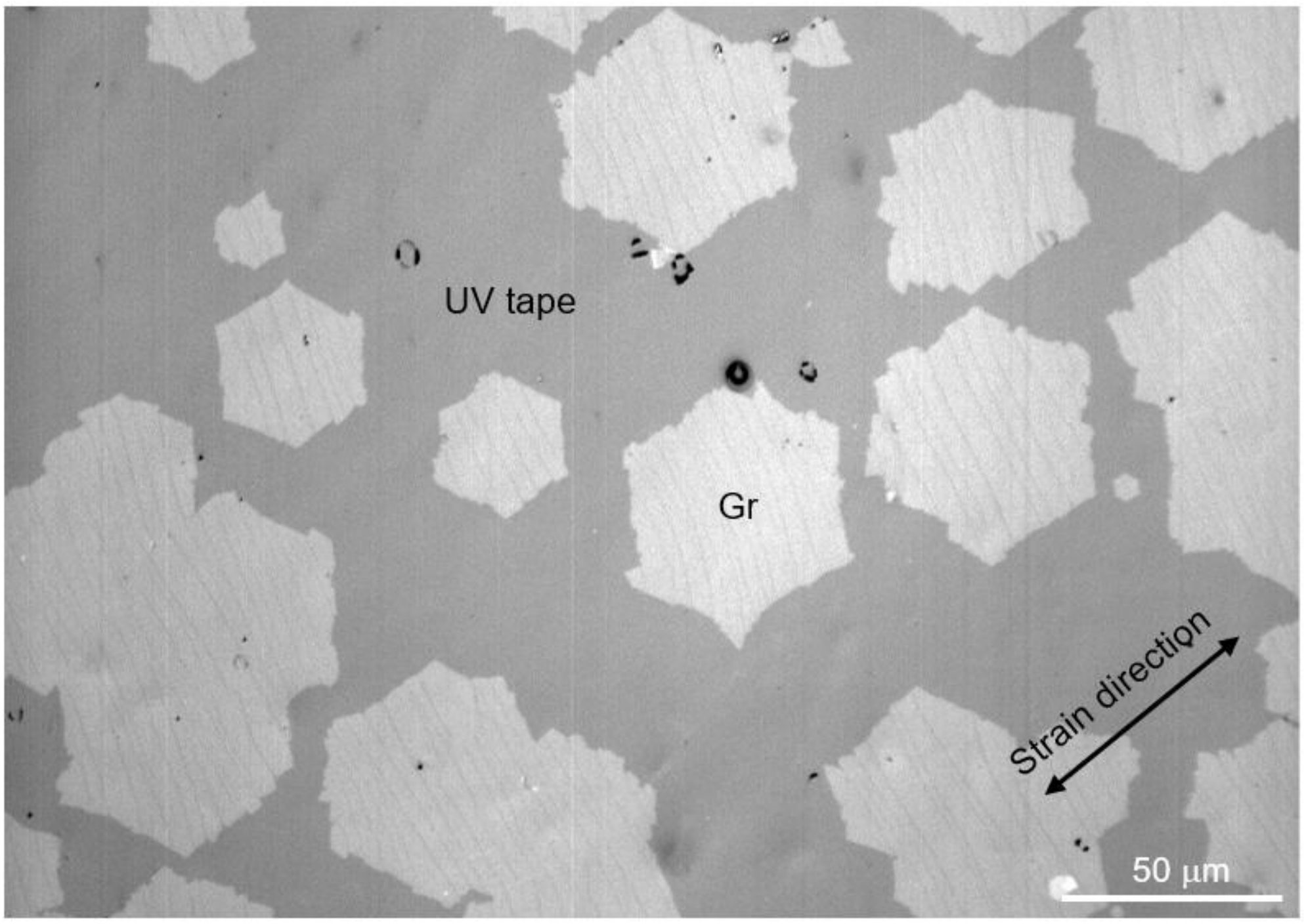

Supplementary Fig. 10 Aligned cracks formed in hexagonal graphene grains. Laser microscope image of hexagonal graphene grains on the UV tape, taken after applying tensile strain to the graphene/UV tape stack. A number of parallel cracks can be seen in the graphene grains, indicating the strain-induced crack formation is not limited to a uniform graphene sheet.

As shown in Supplementary Figs. 1 and 8, we applied tensile strain to the UV tape supporting graphene. When the graphene/UV tape stack was stretched from 0% to 15%, a number of cracks were introduced in the top graphene sheet, running perpendicular to the stretch direction. The adhesion force of the UV tape is weak but still strong enough to induce cracks in the graphene. After reaching the strain around 10%, the crack density did not increase, probably due to slippage of the graphene on the tape surface as well as widening of crack widths. These cracks contribute to form GNSs after adding external stimulus, such as dropping solvent while heating the stack. Distance between of neighboring cracks (i.e., the width of graphene ribbon) basically determined the diameter of the created GNS.

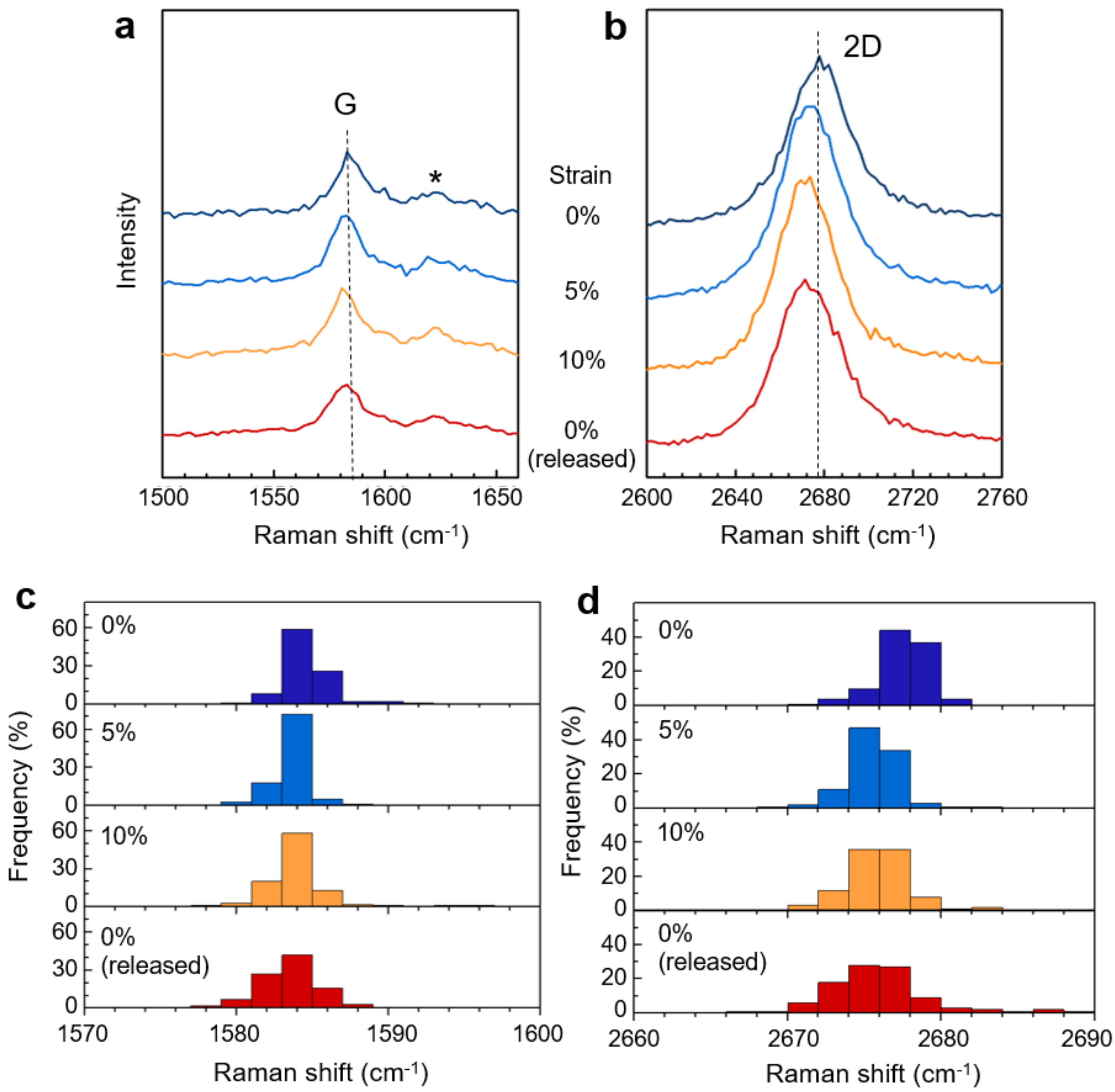

Supplementary Fig. 11. Raman spectra of graphene on the strained UV tape. a,b, Raman spectra of monolayer graphene measured while applying tensile strain to the graphene/tape stack: G band (**a**) and 2D band (**b**). The strain values are nominal values calculated from changes of the tape length. The bottom spectra (red) were taken after releasing the strain of the tape. Asterisk in **a** represents a peak from the adhesive layer of the tape. **c,d,** Distributions of peak positions of G band (**c**) and 2D band (**d**).

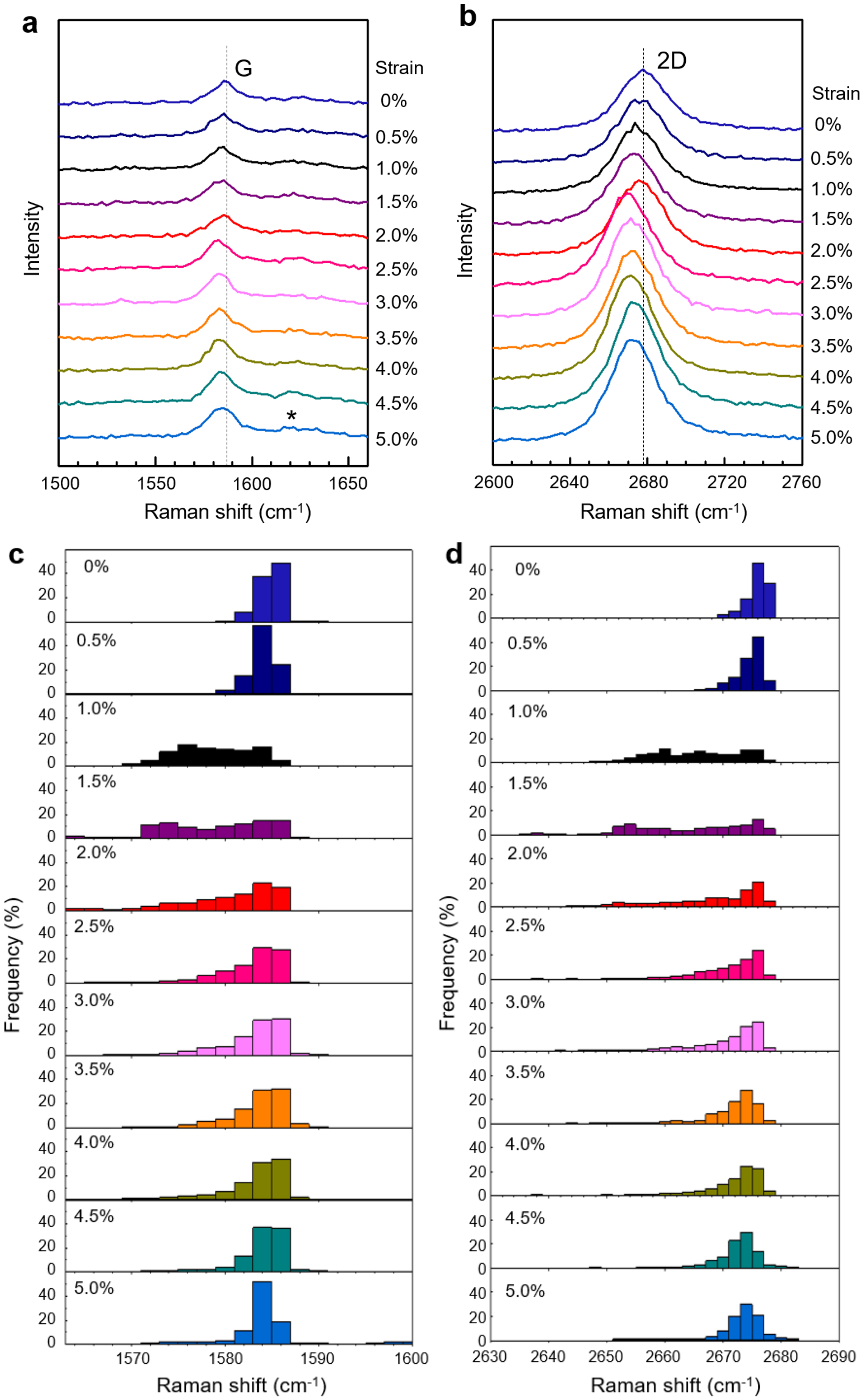

Supplementary Fig. 12. Raman spectra of graphene on the UV tape under low strain loads. a,b, Raman spectra of monolayer graphene measured while applying tensile strain (up to 5%)

to the UV tape: G band (**a**) and 2D band (**b**). The strain values are the nominal values applied to the UV tape. Asterisk in (**a**) represents a peak from the tape. **c,d,** Distributions of the positions of G band (**c**) and 2D band (**d**).

Significant downshifts were observed of both G and 2D bands in the range of 1.0-1.5% strain (Supplementary Fig. 12c,d), indicating the introduction of substantial tensile strain in the graphene. However, when the graphene/UV tape stack was extended to 2.0%, the fraction of spectra exhibiting downshifted G and 2D bands decreased. As the applied strain was increased to 5%, both the 2D and G bands gradually shifted back to higher wavenumbers, as evident in the decrease of the low-wavenumber tails of the histograms. This behavior suggests the onset of crack formation at ≈2.0% strain, followed by the progressive formation of additional cracks with increasing applied strain, which gradually relieves the tensile strain stored in the graphene (but the overall peak distributions are slightly shifted to lower wavenumbers as compared with those before applying strain). In addition, an increase of the 2D band intensity was also observed with increasing the strain, suggesting the slight decoupling from the tape surface.

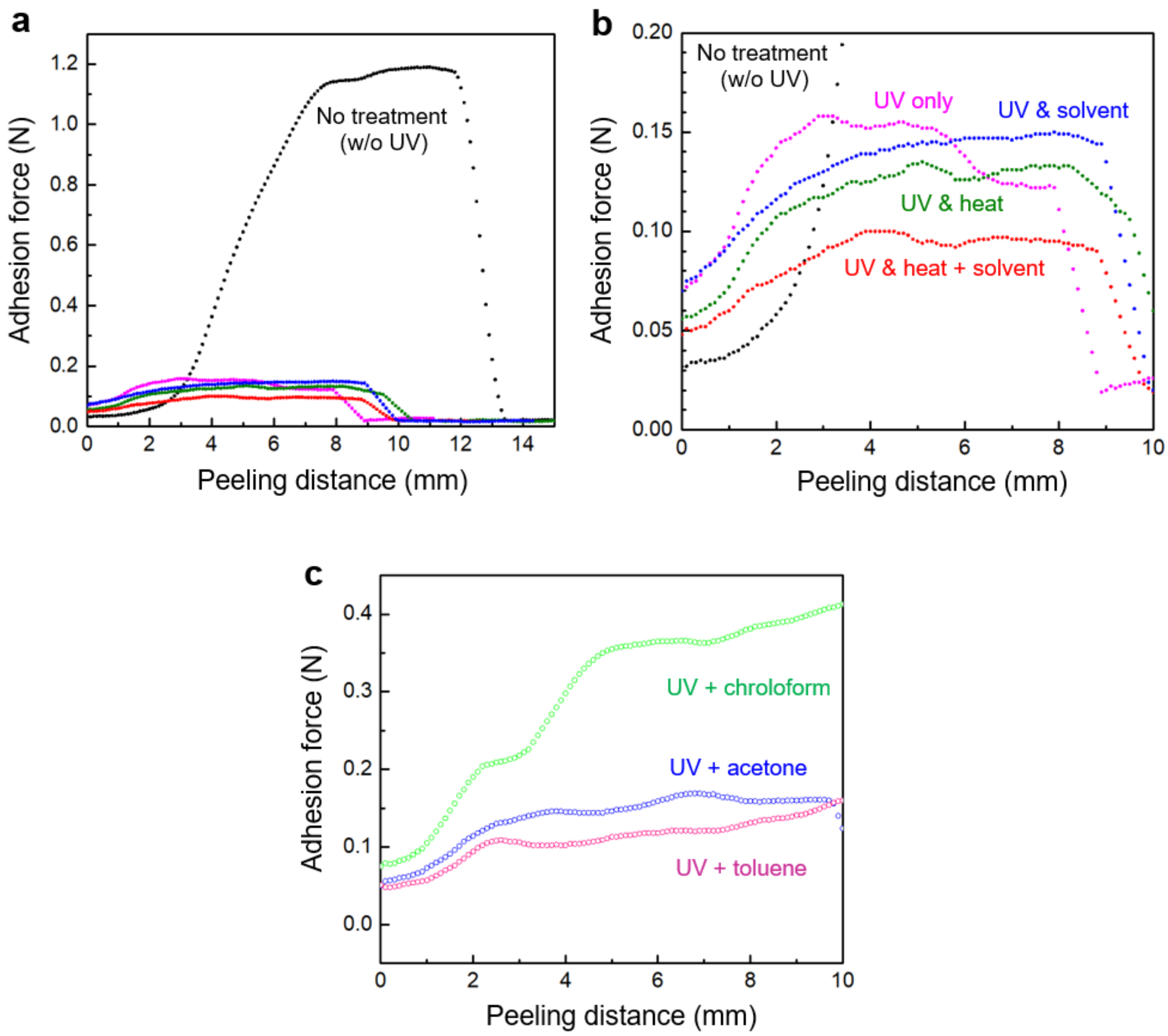

Supplementary Fig. 13. Influence of the post-UV treatment on the adhesion force of the UV tape a, Comparison of adhesion force of UV tapes measured after different treatments. The adhesion force was measured by removing the treated UV tape from a bare SiO_2/Si substrate (10 mm width). No UV illumination (black) maintained strong adhesion force. **b,** After UV illumination the adhesion significantly decreased (pink). The drop of solvent (acetone) slightly decreased the adhesion force (blue), but the heat treatment (80 ^{o}C) was more effective to decrease the adhesion force (green). Interestingly, combining the heat treatment and solvent showed the lowest adhesion force (red). **c**, Solvent dependence of the adhesion force (without heat treatment). To synthesize GNSs from the strained graphene, we used toluene and acetone, which are more effective than chloroform.

As the combination of heat and solvent reduces the adhesion force, the solvent can also contribute to effective detachment of graphene from the tape surface. In addition, intercalation of solvent to the graphene-tape interface can assist the formation of GNSs.

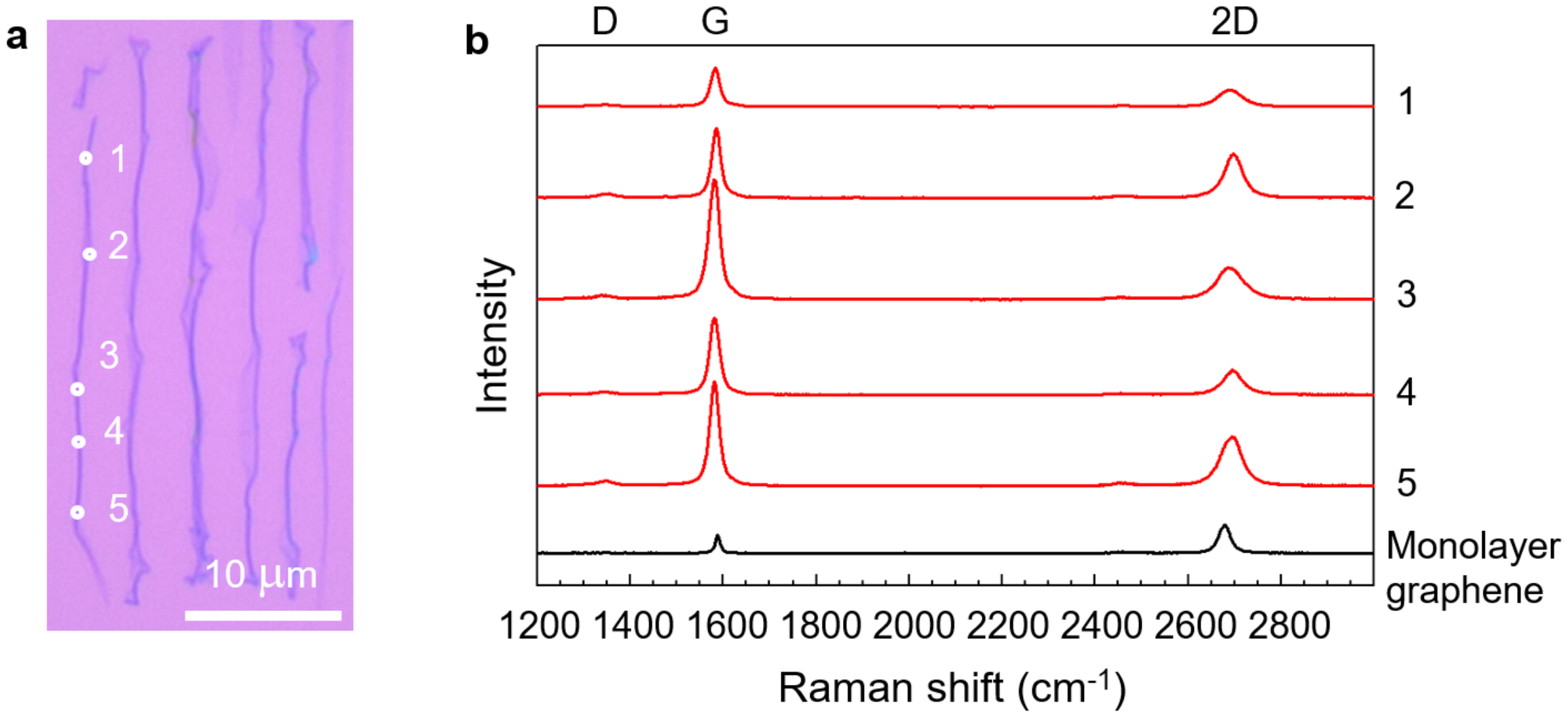

Supplementary Fig. 14. Raman spectra of a GNS array. a, Optical image of aligned GNSs. **b**, Raman spectra of one of the GNSs. The numbers indicate the measured points in **a**. For comparison, a Raman spectrum of monolayer graphene is also displayed.

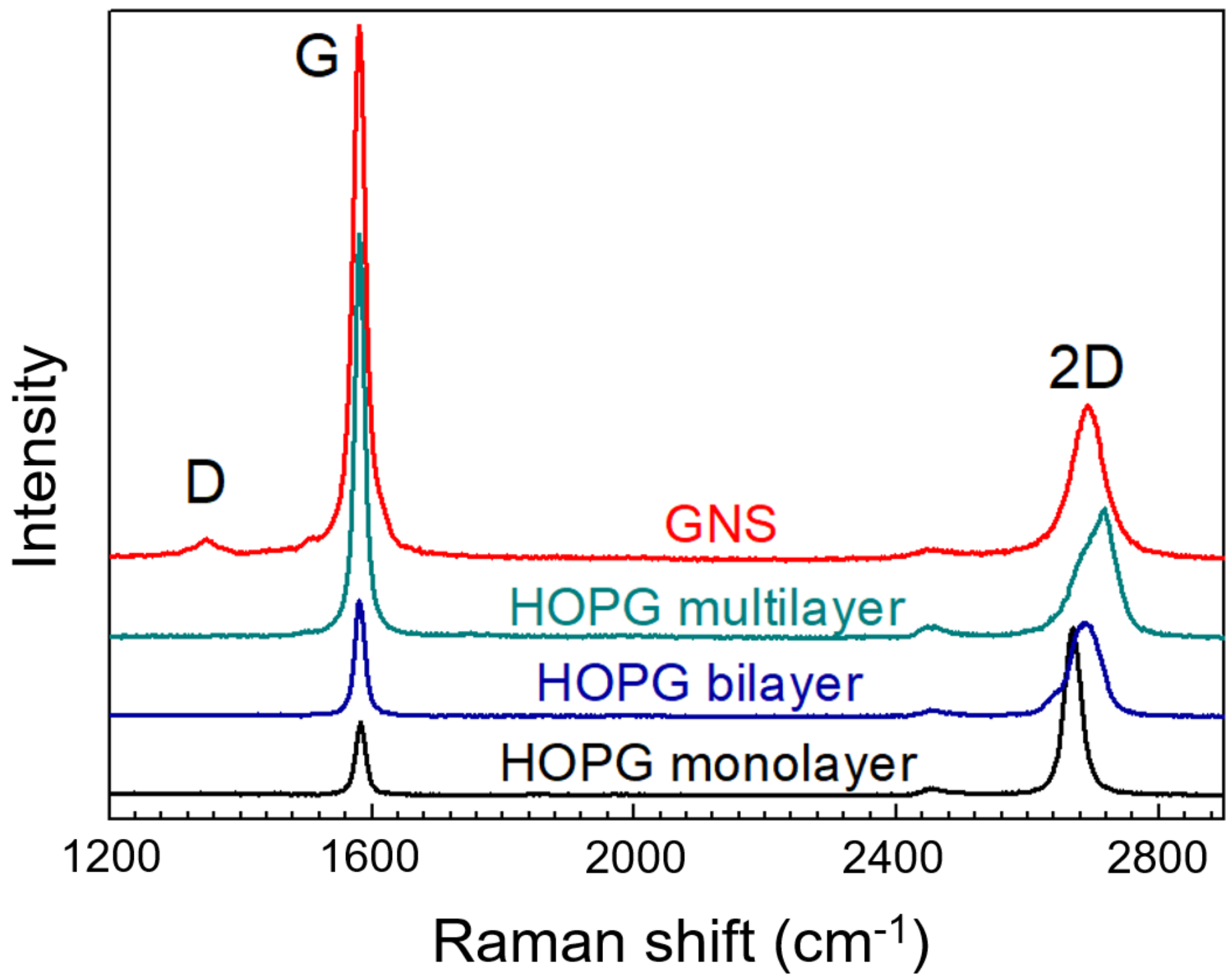

Supplementary Fig. 15. Comparison of Raman spectra of GNS with monolayer, bilayer graphene, and graphite. Monolayer graphene, bilayer graphene, and graphite used for this measurement were exfoliated from highly oriented pyrolytic graphite (HOPG).

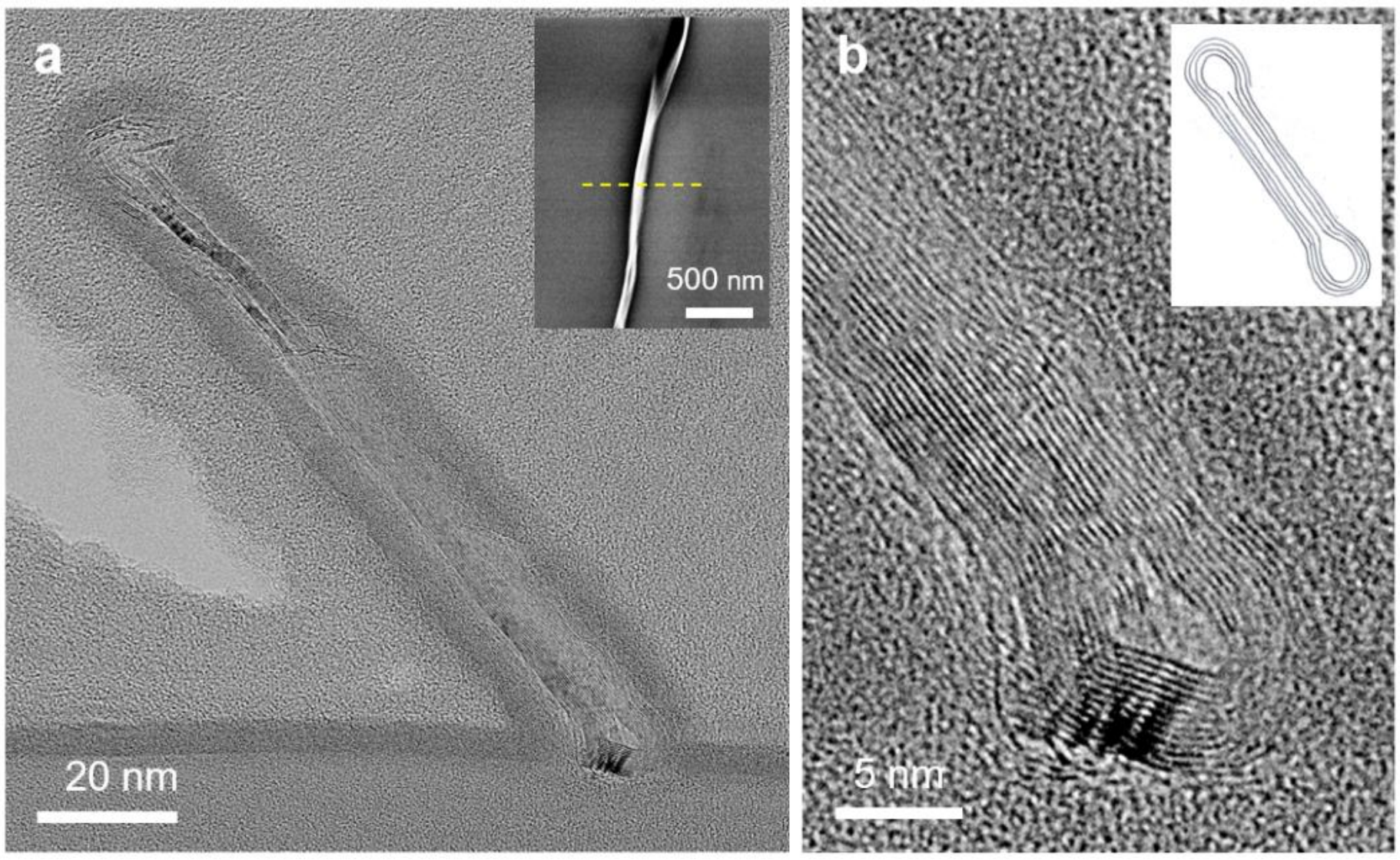

Supplementary Fig. 16. Cross section TEM images of a large-diameter GNS. a, Low magnification TEM image. The dark contrast surrounding the GNS is a protective layer used for the FIB process. Inset shows a SEM image of this GNS, and the TEM specimen was cut from the broken yellow line. **b,** High magnification TEM image. This GNS collapsed due to the large diameter, but two sides exhibit the curved structure, representing the nanoscroll is collapsed to make intra-layer van der Waals interaction. This GNS is tilted with respect to the SiO_2 surface, indicating that the GNS is mechanically stabilized with many scrolled graphene layers. The inset is a schematic of the collapsed nanoscroll.

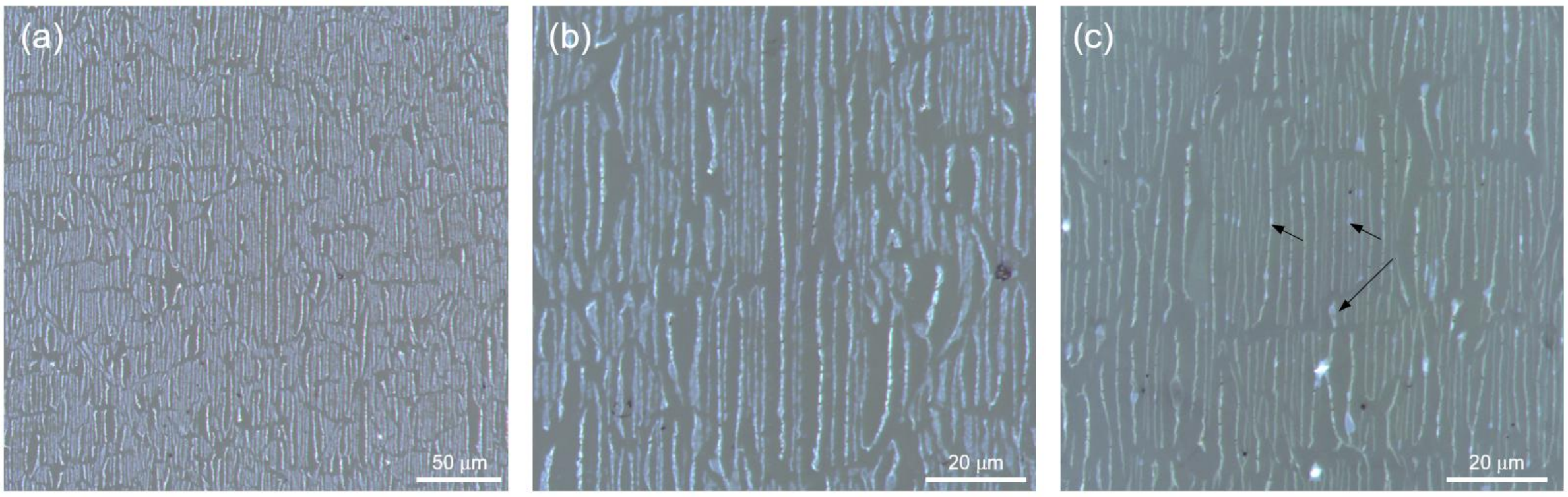

Supplementary Fig. 17. Optical images of MoS_2-graphene co-nanoscrolls. a,b, Most of nanoscrolls consist of MoS_2 and graphene. **c,** MoS_2-graphene heteronanoscrolls using MoS_2 with low coverage. Bright contrast indicated by the arrows show the presence of MoS_2.

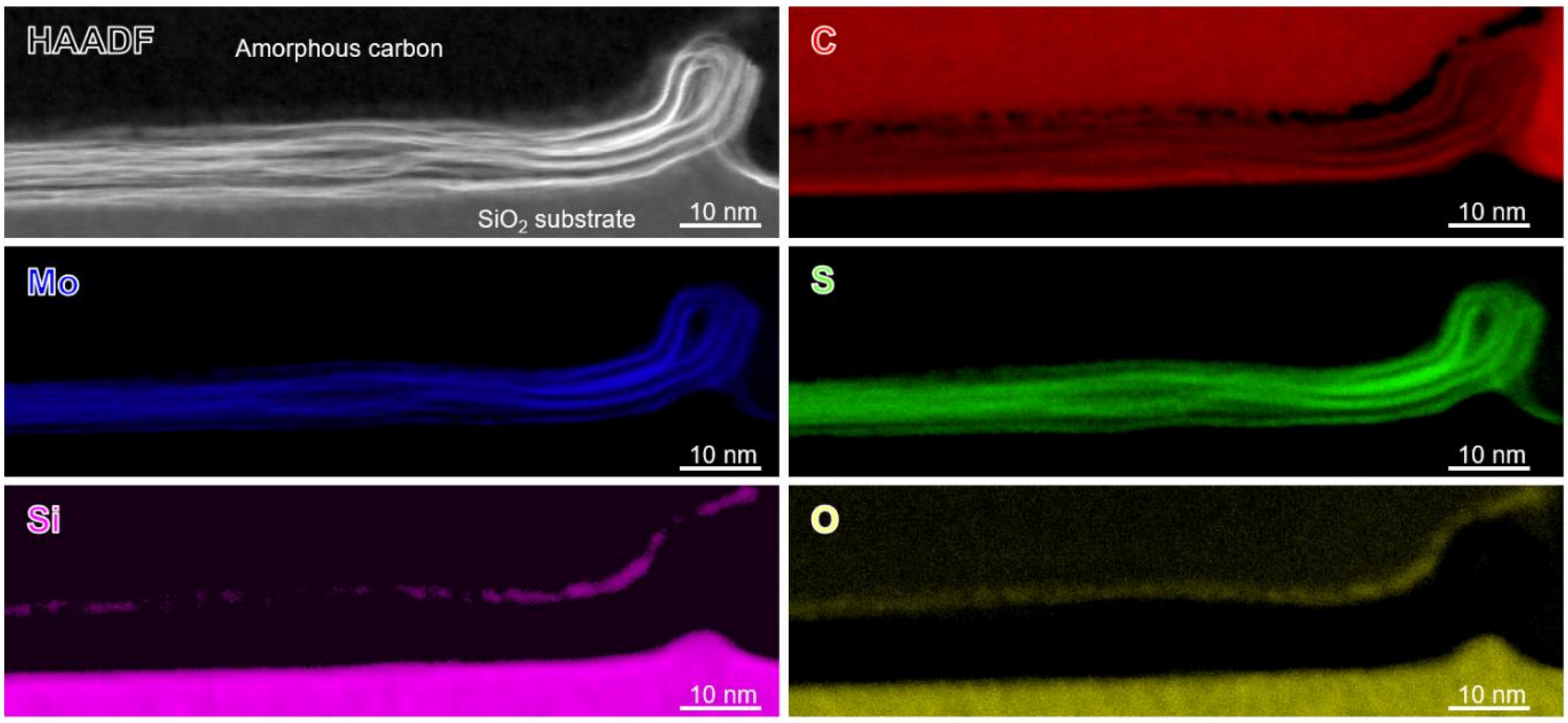

Supplementary Fig. 18. Cross-sectional HAADF image and EELS elemental mapping of the co-nanoscroll. Deposited amorphous carbon on the top were used to protect the sample from damaging during the FIB process.

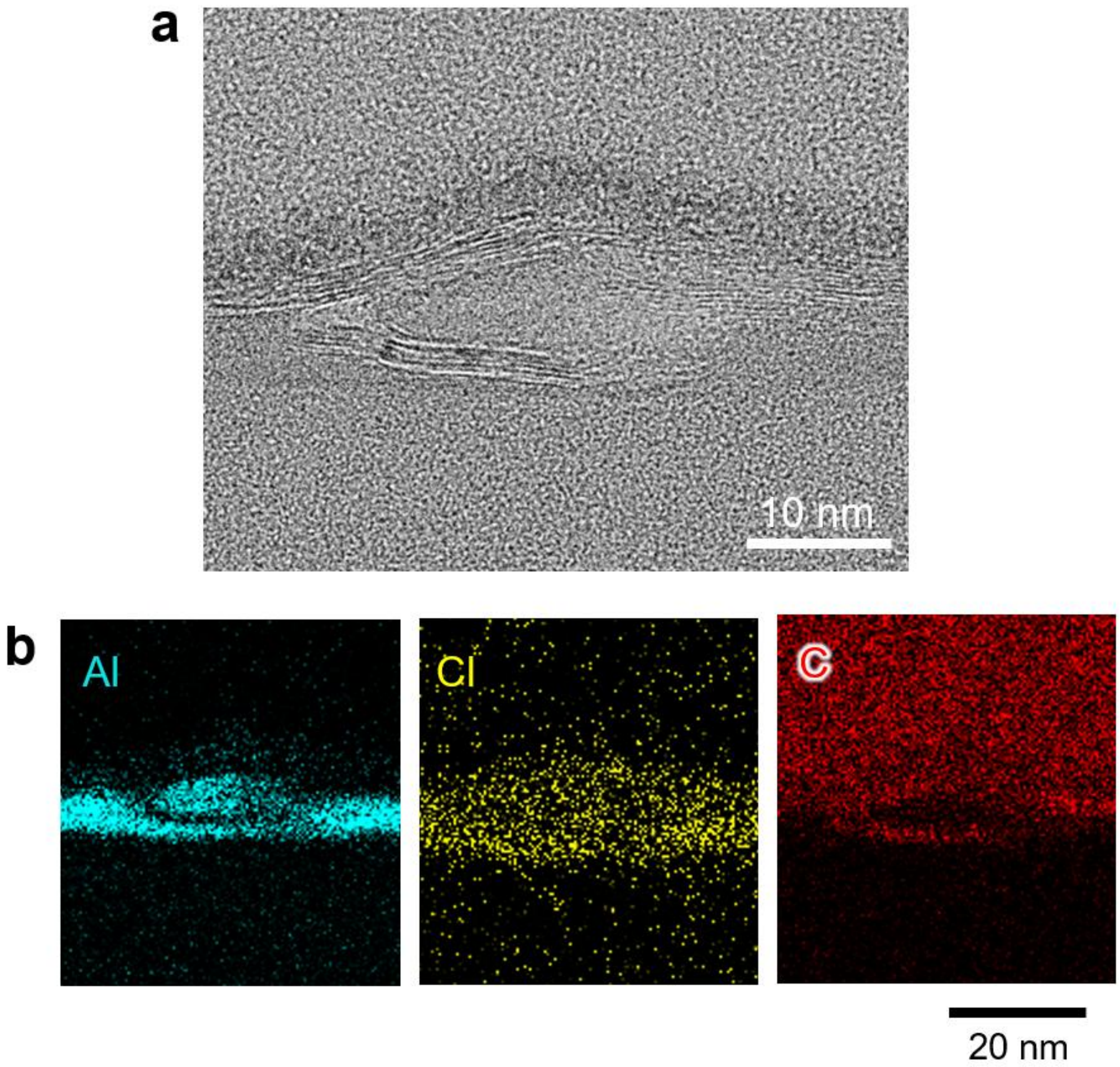

Supplementary Fig. 19. $AlCl_3$-$CuCl_2$@GNS core-shell nanoscroll. Cross-sectional TEM image of $AlCl_3$-$CuCl_2$@GNS core-shell nanoscroll (**a**) and their elemental mappings (**b**). This sample was made by exposing un-scrolled graphene fragment on a SiO_2 substrate to the vapor of $AlCl_3$ and $CuCl_2$. The heat and molecular adsorption induced the scrolling, resulting in the core-shell structure. It is also seen that metal chloride intercalates between the GNS and the SiO_2 surface.

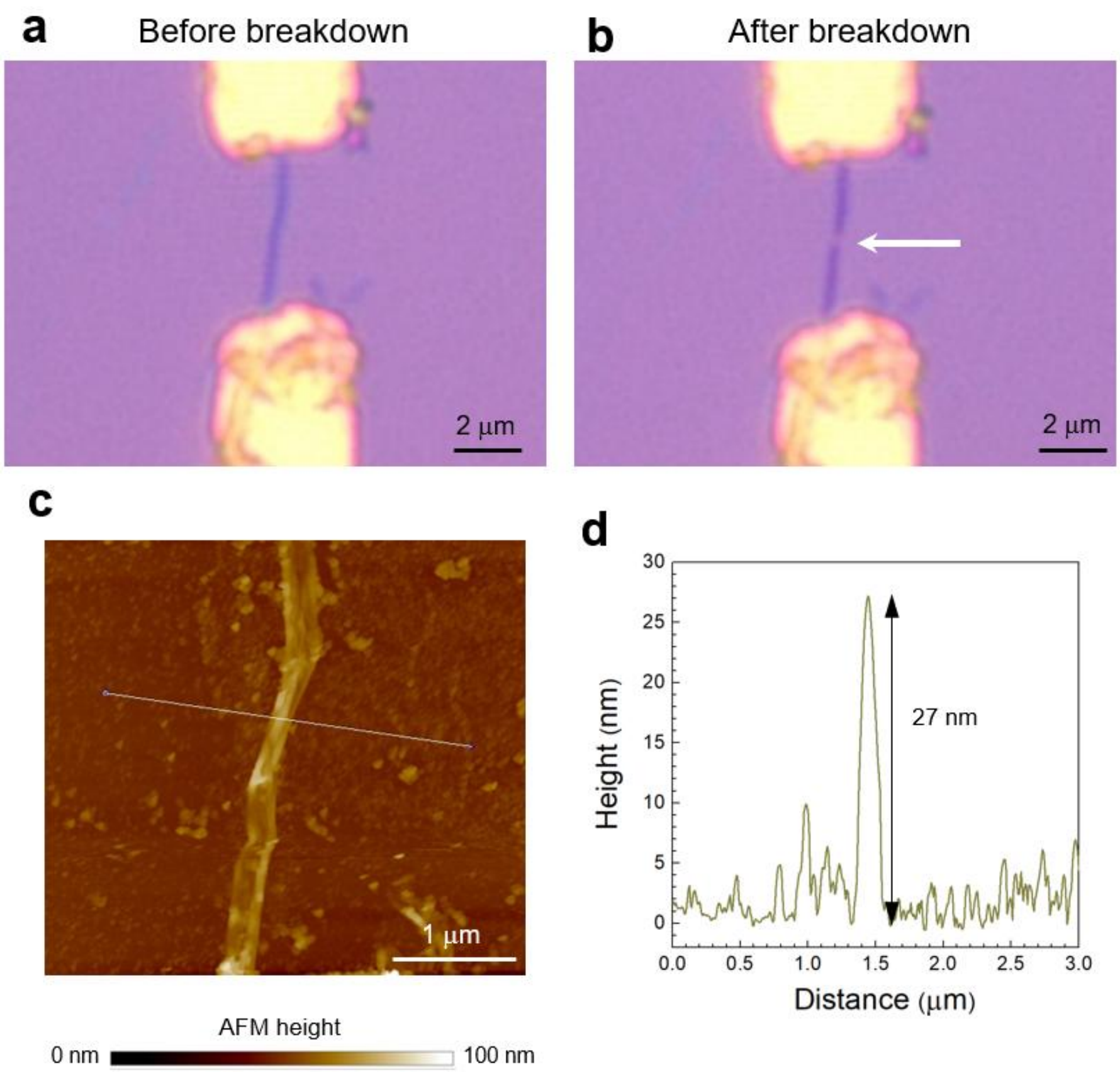

Supplementary Fig. 20. Breakdown measurement of a GNS. Optical image of a GNS measured before (**a**) and after (**b**) breakdown for the device shown in Fig. 6a. The breakdown occurs at the point marked by the white arrow. **c**, AFM image of the GNS measured before the breakdown. **d**, Cross-section of the GNS measured along the line shown in **c**. Note that the actual maximum current density can be higher than the calculated value, because the GNS has a hollow structure.

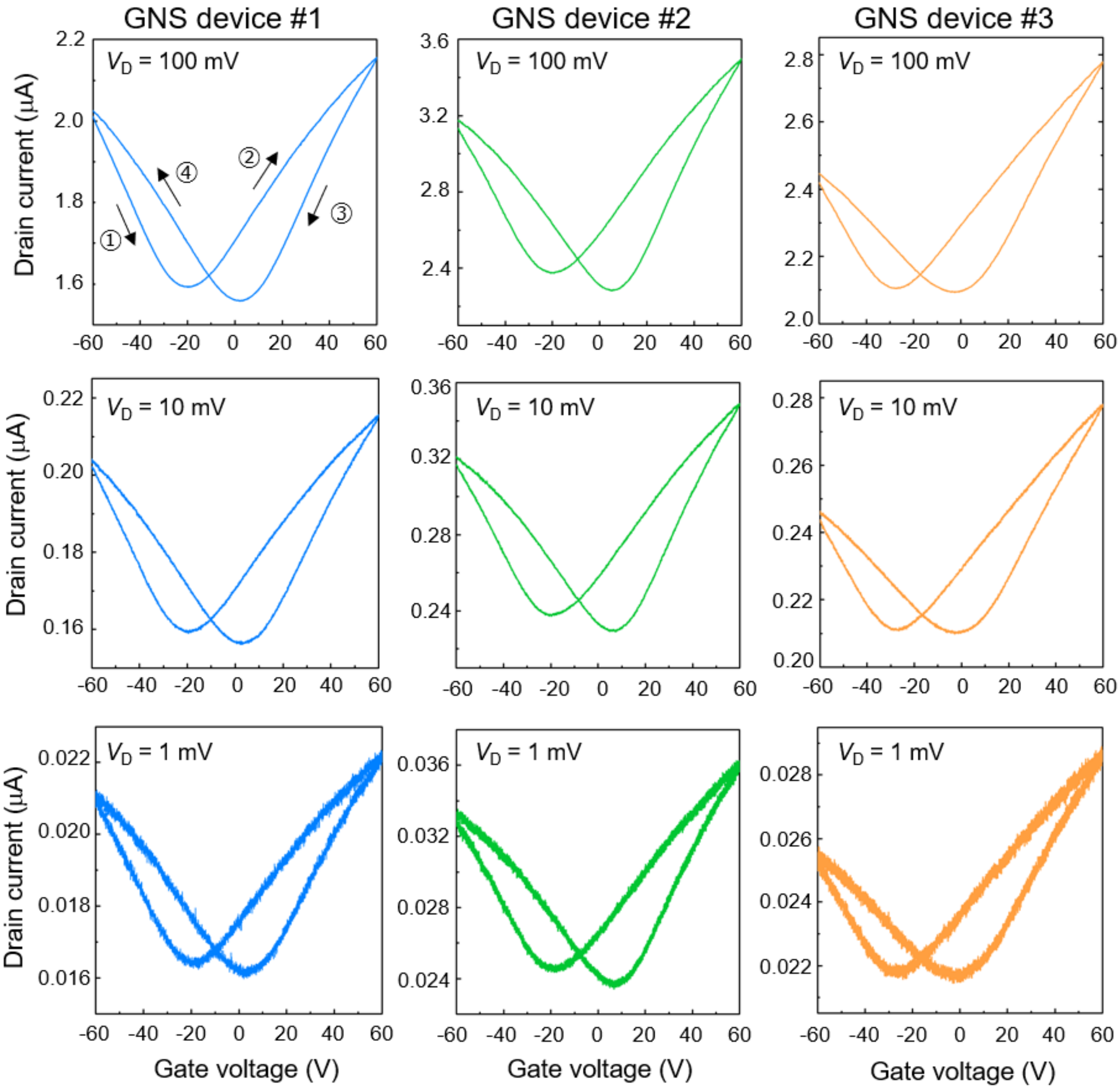

Supplementary Fig. 21. Drain voltage dependence of GNS-FETs. Transfer curves of three GNS-FETs measured at three different drain voltages (100, 10, and 1 mV). Hysteresis was observed regardless of the applied drain voltage.

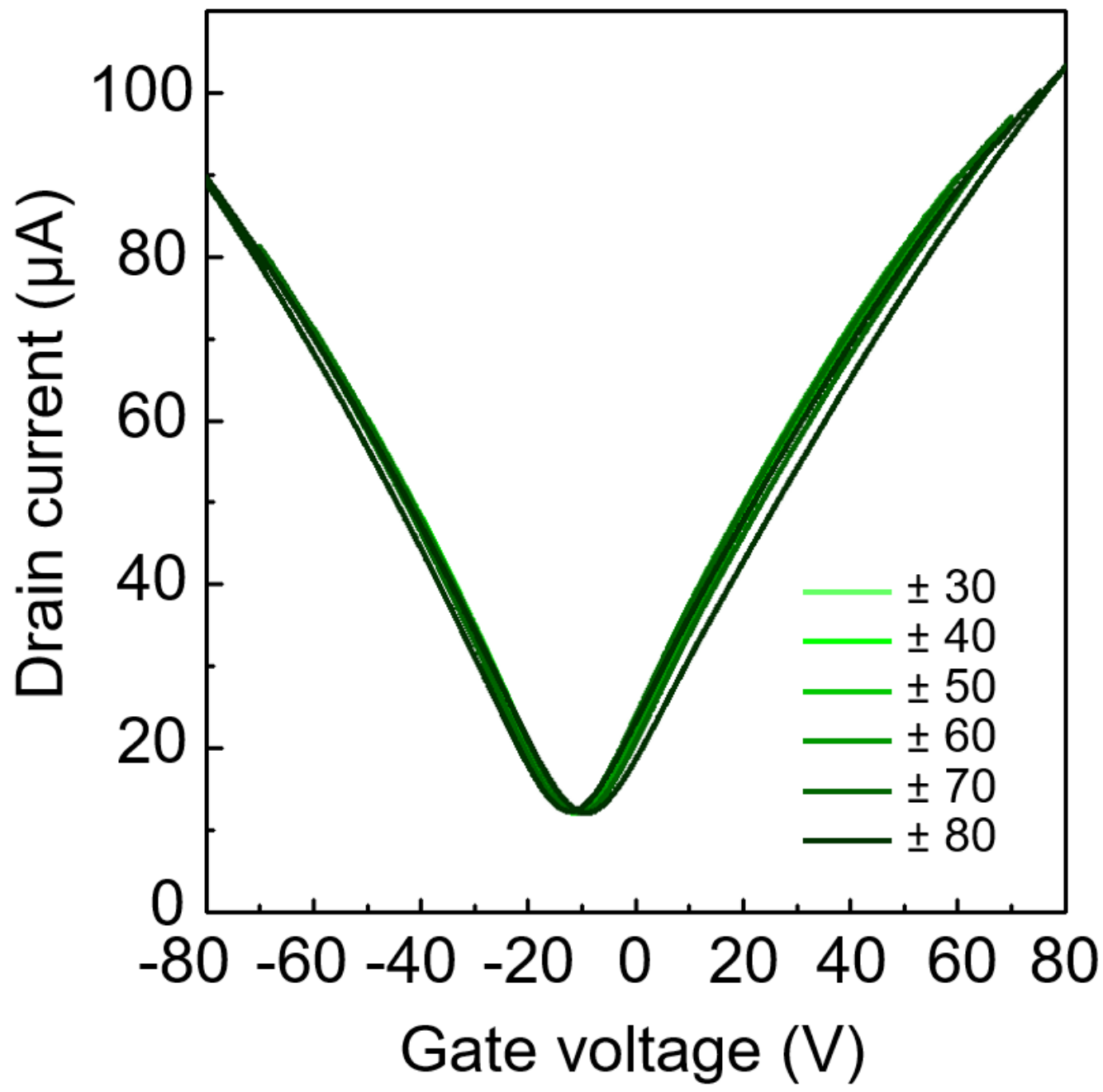

Supplementary Fig. 22. Negligible hysteresis in a graphene-FET. Transfer curves of a graphene-FET measured at six different sweep gate voltage ranges (from ±80 to ±30 V with a 10 V step). In contrast to the GNS-FET (shown in Fig. 6b), a graphene sheet did not show apparent hysteresis in the transfer curves.

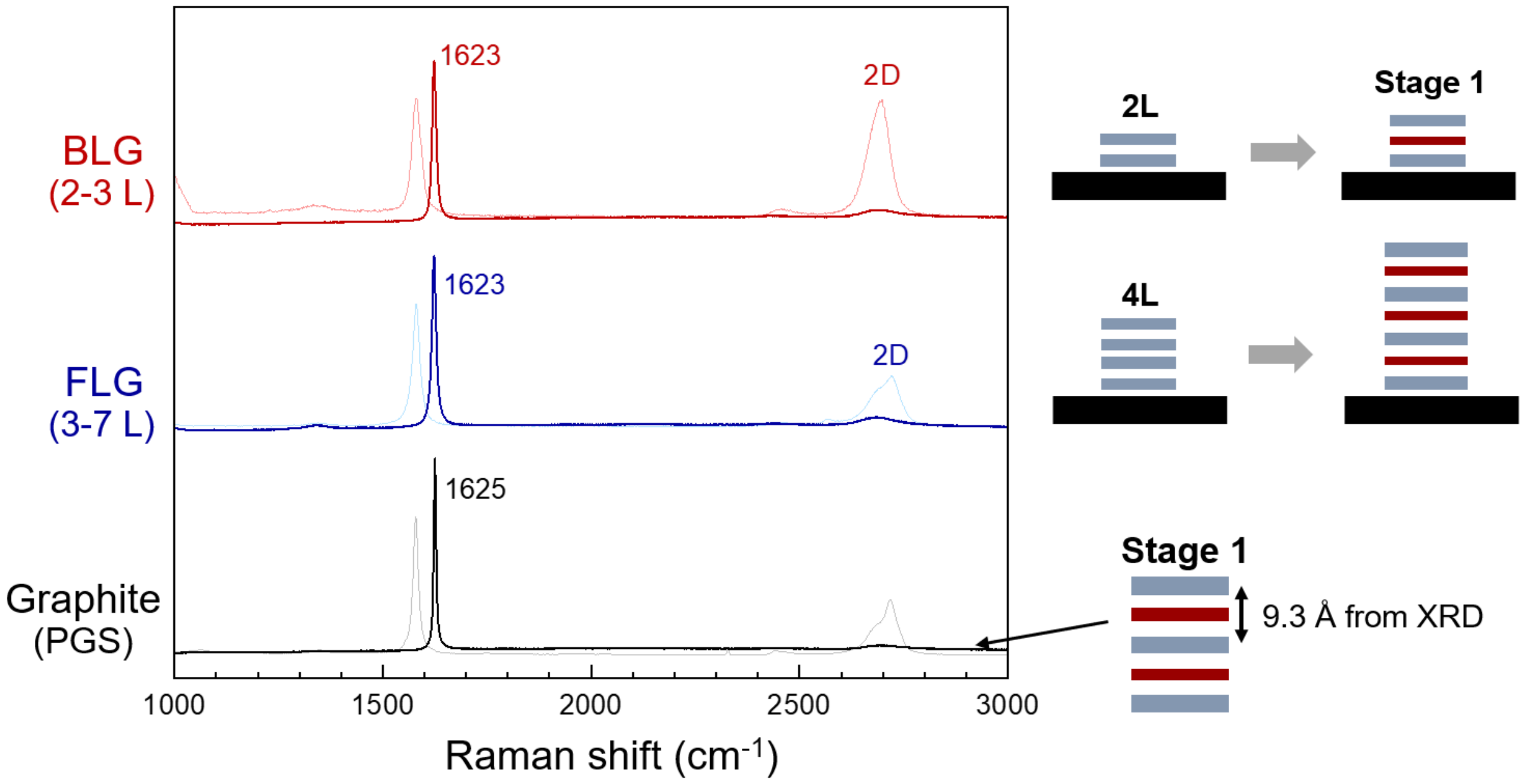

Supplementary Fig. 23. Raman spectra of fully intercalated graphene and graphite. Bilayer graphene (BLG) with 2-3 layers, few-layer graphene (FLG) with 3-7 layers, and graphite (pyrolytic graphite sheet (PGS)) were used to intercalate $FeCl_3$ molecule. The Raman measurement was performed in vacuum without exposing the samples to air. Light and dark colors show the Raman spectra of the pristine and intercalated samples, respectively. The metal chloride intercalation induces large upshift of the G band to 1623-1625 cm^{-1} with strong suppression of the 2D band due to hole doping.

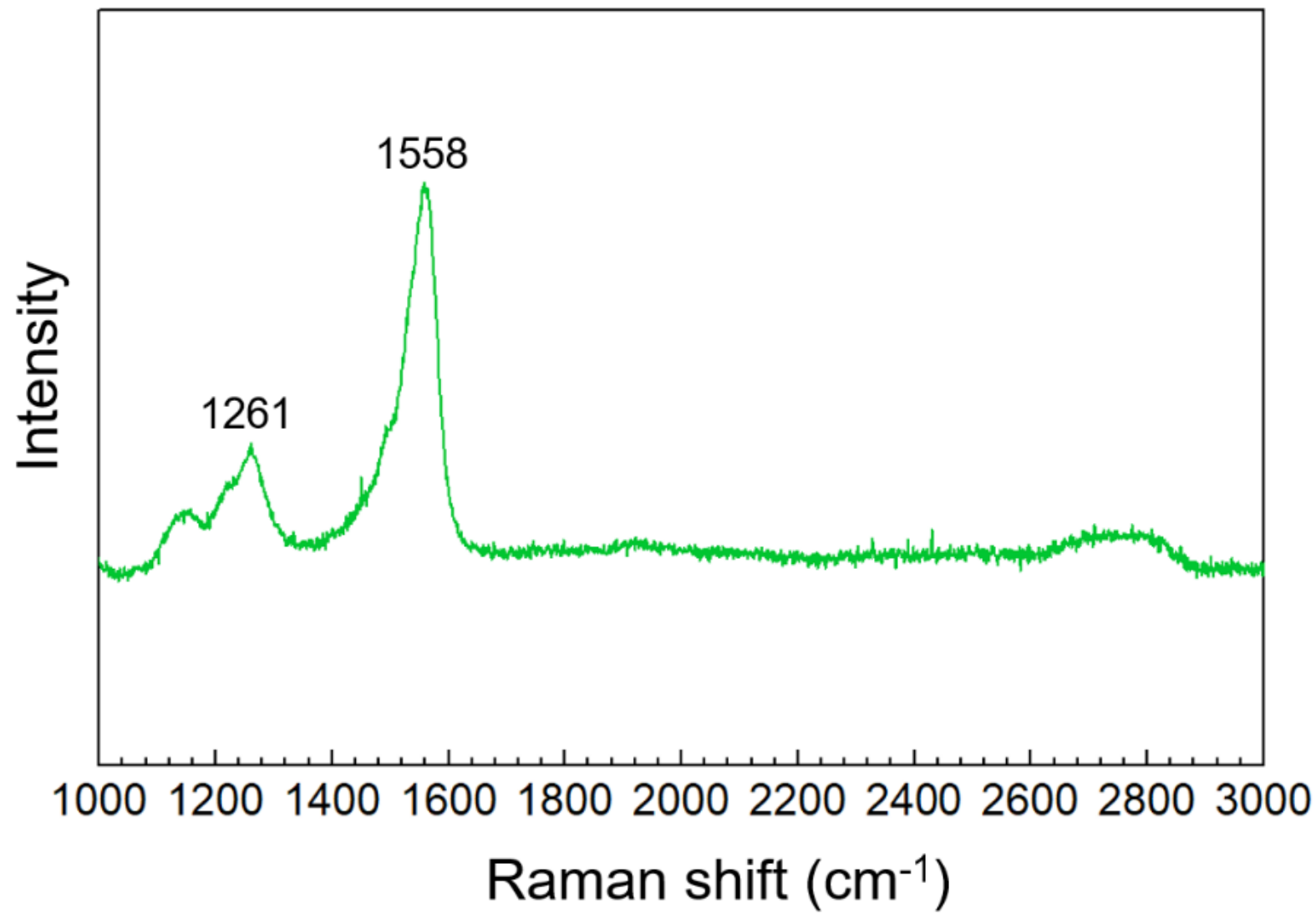

Supplementary Fig. 24. Raman spectrum of K-intercalated GNS. Raman spectrum was measured without exposing air. The observed redshift of the G band to 1558 cm^{-1} indicates the effective n-type doping to the GNS host.

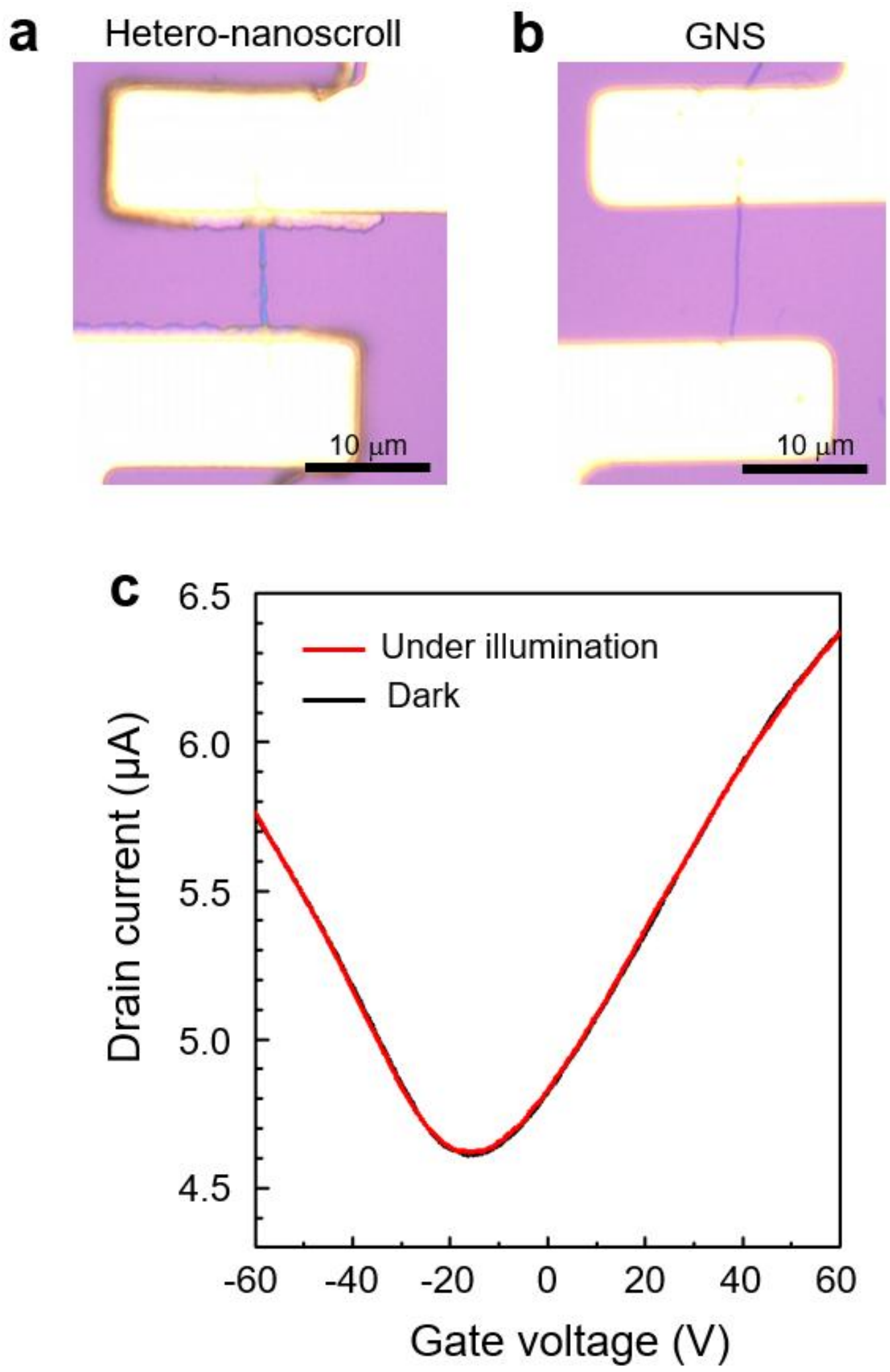

Supplementary Fig. 25. Optical images of heteronanoscroll and GNS devices and optical response. Optical micrographs of a heteronanoscroll (**a**) and GNS (**b**) FETs. It can be seen that the heteronanoscroll has stronger optical contrast than GNS due to the presence of MoS_2. **c** Transfer curve of the GNS-FET in dark (black) and under 532 nm illumination (red) at V_D = 100 mV. No photoresponse was observed in the GNS-FET.

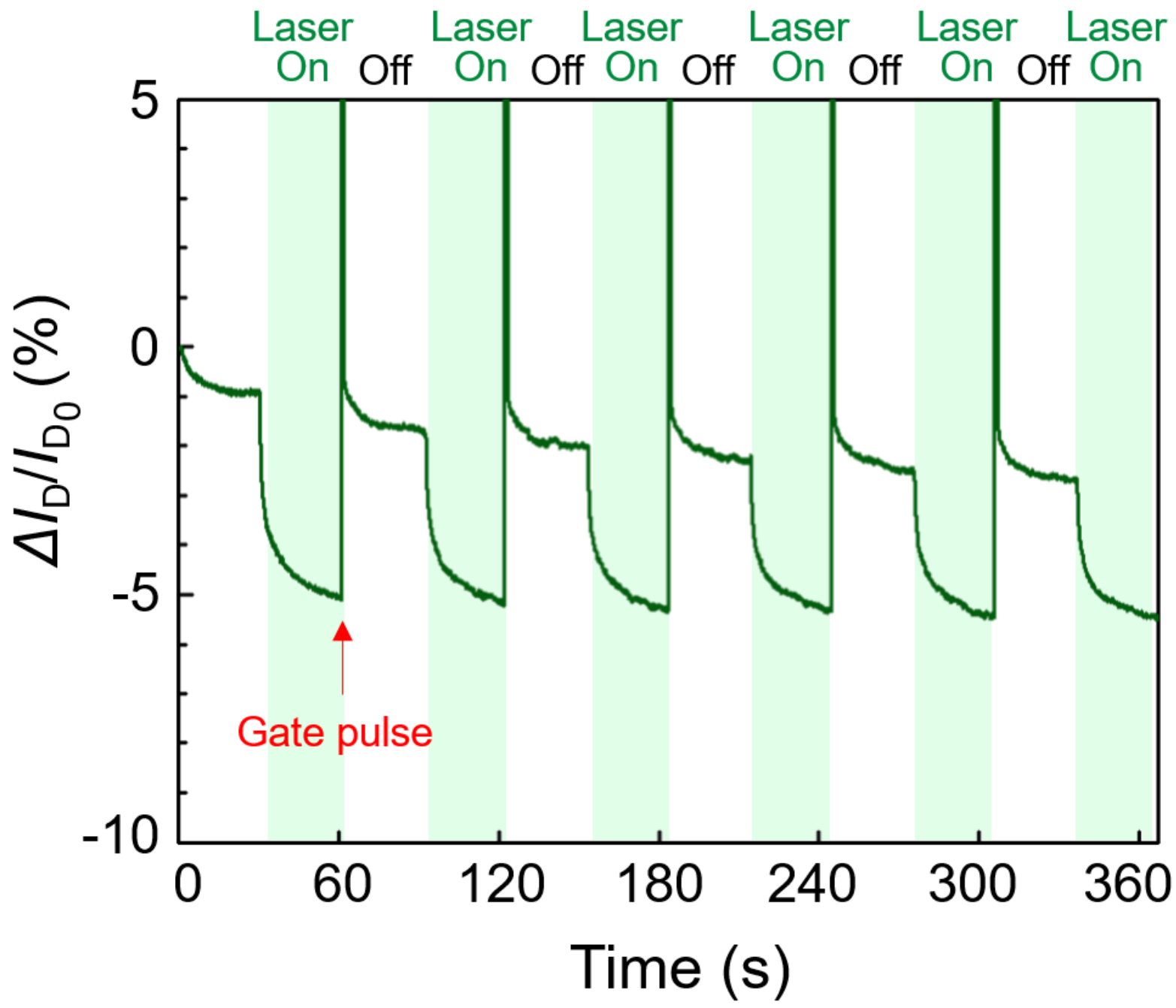

Supplementary Fig. 26. Sequential control of the drain current with the illuminated light. The drain and gate voltages are 0.1 and -50 V, respectively, and the gate pulses of 60 V were applied to refresh the device. The device demonstrated a stable photoresponse with the aid of the gate pulses.

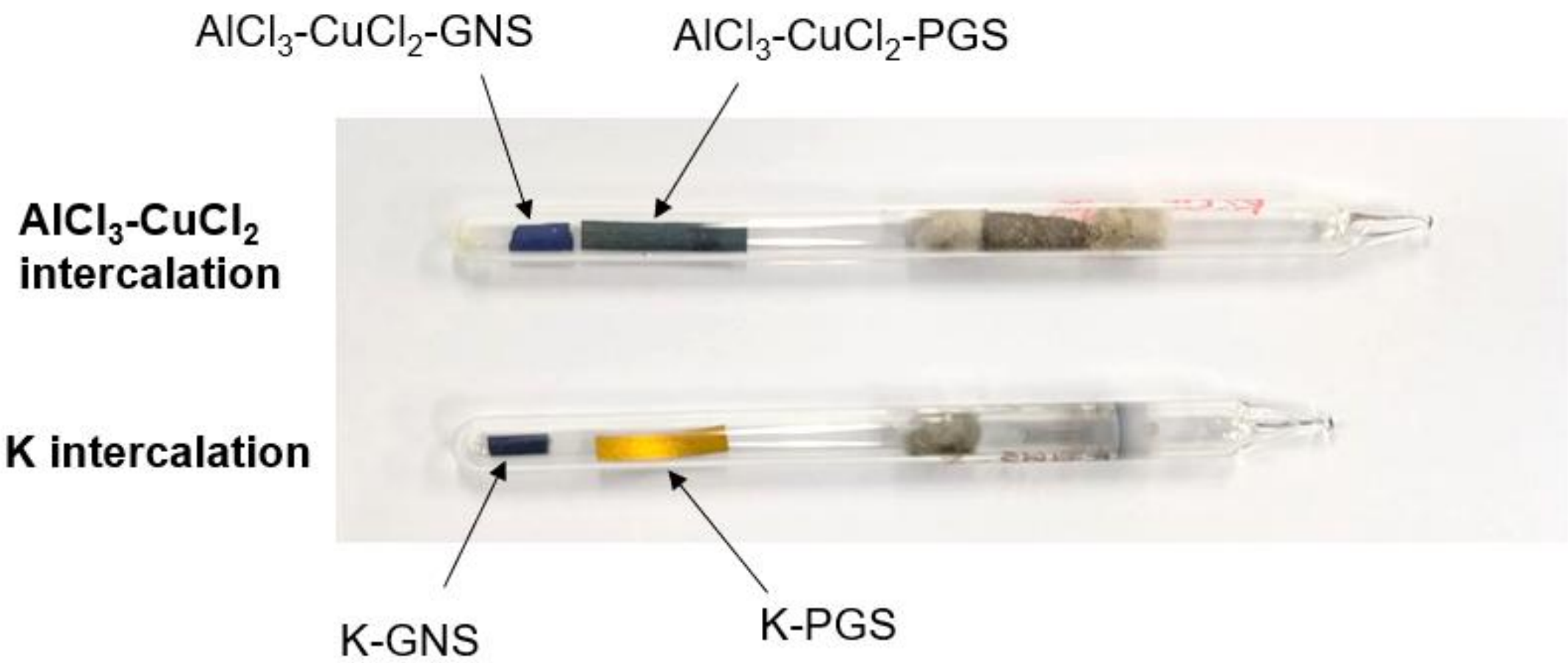

Supplementary Fig. 27. Photograph of the glass tubes after the intercalation. This photograph was taken after the intercalation of $AlCl_3$-$CuCl_2$ (top) and K (bottom) to GNS. PGS was also enclosed in order to monitor the progress of the intercalation from its color change.

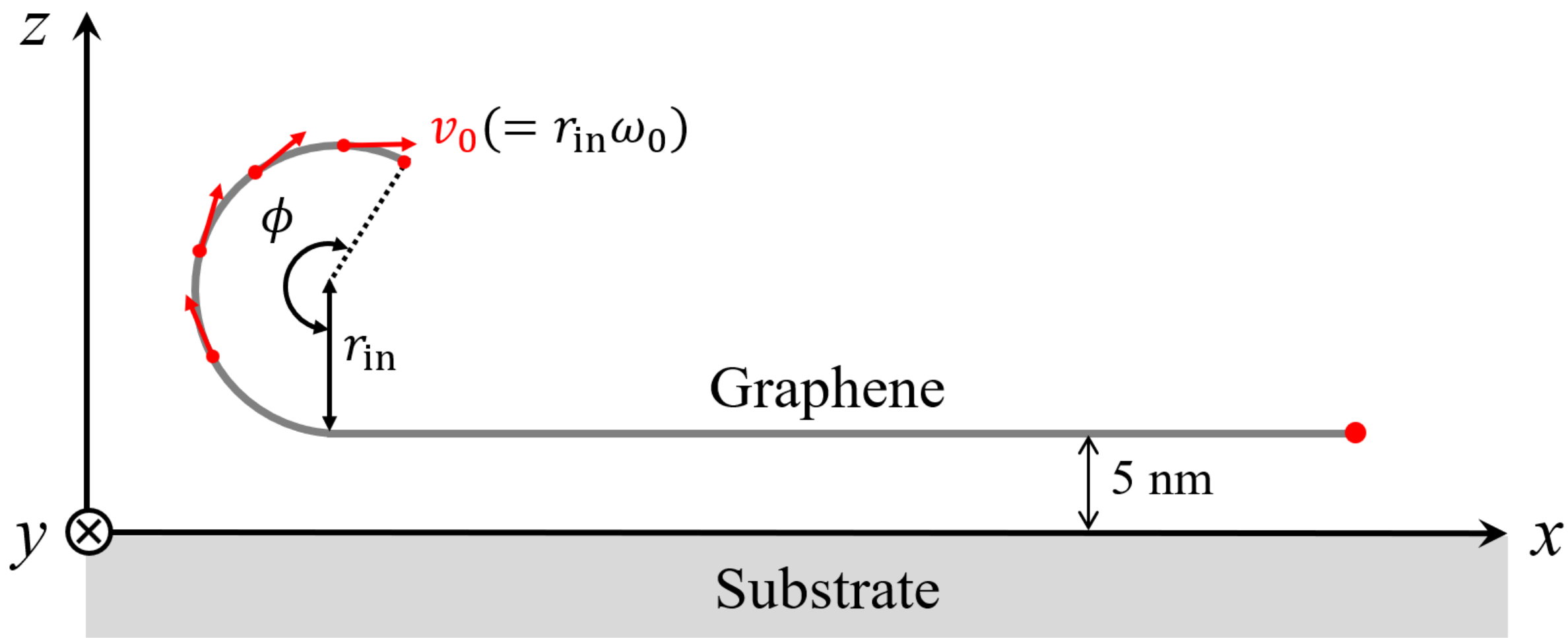

Supplementary Fig. 28. Schematic of the initial GNS configuration. The scrolled region is modeled as an Archimedean spiral defined by the inner radius r_{in} and the azimuthal angle. To keep the clearance available after fracture/delamination from the UV tape, the flat graphene region is initially positioned ~5 nm above the effective substrate plane. The carbon atoms at the left-edge side of the GNS, highlighted by the red circles, are constrained: their motion in the directions parallel to the substrate plane (*x*-*y* plane) is fixed, while movement in the perpendicular (*z*) direction is allowed. Red arrows denote the initial tangential velocities (corresponding to an angular velocity ω_0) assigned to the carbon atoms in the scrolled region. A planar wall potential is placed at the effective substrate plane and acts when atoms approach within the cutoff distance during the rolling process.
