# Supplementary material for: Strain-engineered fabrication of high-density aligned graphene-based nanoscrolls via UV-tunable adhesive tapes
Source: Nat Commun. 2026 Oct 21;17:10647. doi: 10.1038/s41467-026-78264-8 (PMC13687067; doi:10.1038/s41467-026-78264-8)
Supplement: Supplementary file 2 [file 41467_2026_78264_MOESM2_ESM.pdf]

Description of Additional Supplementary File

Supplementary Movie 1. Evolution of the strain distribution in the UV tape determined by DIC method, which was measured while applying tensile strain.

Supplementary Movie 2. In-situ manipulation of graphene ribbons with a sharp probe inside the SEM chamber.

Supplementary Movie 3. MD simulation of the GNS formation at temperature of 0 K. Graphene nanoribbons with different initial curvatures are calculated upon strain relaxation.

Supplementary Movie 4. MD simulation of the GNS formation captured from a different view angle.

Supplementary Movie 5. Comparison of GNS MD simulation at 0 K and 90 K.
