# Supplementary material for: Strain-engineered fabrication of high-density aligned graphene-based nanoscrolls via UV-tunable adhesive tapes
Source: Nat Commun. 2026 Oct 21;17:10647. doi: 10.1038/s41467-026-78264-8 (PMC13687067; doi:10.1038/s41467-026-78264-8)
Supplement: Supplementary file 4 [file 41467_2026_78264_MOESM9_ESM.pdf]

nature portfolio

Peer Review File

Strain-engineered fabrication of high-density aligned graphene-based nanoscrolls via UV-tunable adhesive tapes

Corresponding Author: Professor Hiroki Ago

Parts of this Peer Review File have been redacted as indicated to remove third-party material.

This file contains all reviewer reports in order by version, followed by all author rebuttals in order by version.

Version 0:

Reviewer comments:

Reviewer #1

(Remarks to the Author)
This work utilizes functional tape with ultraviolet (UV) light-tunable adhesion to achieve the large-scale synthesis of graphene nanoscrolls (GNSs) through strain regulation. This strain-assisted method is efficient and also enables the control of the scrolling angle (i.e., the chiral angle) by manipulating the stretching direction. The work promises to be a significant contribution to the field, but more comprehensive studies are needed prior to publication:

1.The strain-regulation method using functional tape with UV-tunable adhesion allows control of the scrolling angle by varying the stretching direction. The main novelty of this method is the high yield and good control on the rolling direction. However, we did not observe sufficient demonstration on tunning the scrolling angles, particularly the chiral angles, analysis on the controllability of scroll angles etc. in the manuscript. Is the scrolling direction solely determined by the direction of the applied tensile strain?
2.Optical microscopy observations reveal non-uniform morphology of the graphene nanoscrolls (e.g., Fig. 1d). What causes this non-uniformity? Can the synthesis be optimized to yield higher-quality GNSs?
3.The electronic properties of graphene scrolls need more comprehensive study. Except sustain higher current density, what is the advantage of graphene scrolls? The measurements in Fig. 5 seems indicate the graphene scroll show neglectable gate effect, thus what kind of application is expected in electronics?
4.Do the low-frequency Raman breathing modes (LBM) of the graphene nanoscrolls exhibit differences depending on the scrolling angle and diameter?
5.Can the preparation of graphene-based heteronanoscrolls maintain the similarly high efficiency demonstrated for pure GNSs? Could corresponding characterization data (e.g., optical microscopy) be provided?
6.Does the intercalation with metal ions alter the band structure of the graphene scrolls?

Reviewer #2

(Remarks to the Author)
This manuscript reports on a large-area synthesis of dense graphene nanoscrolls (GNSs) with controlled scrolldirection by utilizing a functional tape, whose adhesive strength can be tuned with ultraviolet light. The authors demonstrate that stretching the tape applies tensile strain to graphene, resulting in the formation of vertically oriented GNSs during peeling. The ultrathin structure of graphene is difficult to manipulate, while the nanoscroll structure is beneficial for metal chloride and alkali metal intercalation, enhancing conductivity. This method is also extended to the preparation of GNS/MoS_2 heterostructures, providing a new approach for one-dimensional heterostructures. However, several critical aspects require clarification to fully substantiate the claims and solidify the mechanistic understanding of the process. Specifically, many of the produced scrolls exhibit twisted or wavy morphologies rather than being perfectly rectilinear, and their winding tightness appears to vary significantly. These are important structural inconsistencies that are likely to impact the intrinsic properties of the GNSs and necessitate further investigation.Also,graphene nanoscroll with certain scrolldirection have been achieved by Wenping Hu,et,al ,so the novety is also a question. Also with certain Given that these fundamental issues regarding structural uniformity and formation mechanism remain unresolved, I cannot recommend publication in Nature Communications in its current form.

1.The authors claim that tensile strain is uniformly applied to graphene across the tape surface, leading to aligned GNS arrays. However, no quantitative characterization of strain distribution is provided. For large-area graphene films, strain gradient is inevitable due to edge effects and uneven adhesion between the UV tape and graphene. The paper only mentions periodic linear cracks perpendicular to the strain direction but lacks statistical data on crack density, width, and spacing, as well as their correlation with GNS density and alignment degree. It is recommended to supplement micro-Raman mapping of strain distribution (based on G-band shift) across the large-area graphene, and establish quantitative relationships between strain magnitude, crack parameters, and GNS structural characteristics (density, length, chiral angle).
2.The provided SEM and optical images (e.g., Fig. 1c, 1g, and Supplementary Fig. 5) reveal that a significant number of the nanoscrolls exhibit noticeable morphological defects. Specifically, many scrolls appear twisted or wavy rather than perfectly rectilinear, and the winding tightness seems to vary significantly, which are critical structural flaws that may affect their intrinsic properties. The authors should provide a more critical discussion on the origins of these morphological defects. Additionally, for the fabrication of graphene–MoS_2 heteronanoscrolls, the reproducibility or yield, nor the degree of structural uniformity (e.g., layer sequence consistency or scroll completeness) are discussed. Clarifying how reliably such heteronanoscrolls can be produced over larger areas would be important for assessing their practical relevance and scalability.
3.The correlation between Raman signals and specific structural parameters (number of rolls, interlayer spacing, residual strain, etc.) is not quantitatively established. The authors attribute the low I2D/IG ratio, broadened 2D band, and G-band red shift to multi-layer coupling and residual strain, but no control experiments with different strain magnitudes (e.g., 3%, 5%, 7% extension degree) or GNS layer numbers are performed to verify these attributions.
4.Molecular dynamics (MD) simulations are conducted at 0 K, which deviates significantly from the experimental conditions involving 90 °C heating and organic solvent treatment. Though the authors claim that thermal effects are not essential for the scrolling phenomena examined in the study, it still remains possible for them to remarkably affect graphene's flexibility, interlayer van der Waals interactions, and scrolling dynamics. Additionally, the MM calculations only analyze the energy balance between bending and van der Waals interactions, but do not involve the effect of solvent molecules on interlayer interactions.
5.For the atomic simulation part, the size, boundary conditions of the simulation system and the basis for force field selection need to be clarified. The paper mentions that "initial edge curvature" is a triggering factor, but fails to explain how to regulate the edge curvature of graphene. Could you supplement the regulation method of edge curvature and corresponding experimental verification to further strengthen the rationality of the mechanism?

Reviewer #3

(Remarks to the Author)
This manuscript presents a highly efficient approach for the large-scale synthesis of graphene nanoscrolls (GNSs) with controlled orientation, utilizing a UV tape. The authors demonstrate that applying tensile strain via tape stretching, followed by the application of external stimuli, successfully yields dense arrays of aligned GNSs. While the findings reported here present a new approach, key parameters in the manuscript are lacking. The authors measure a variety of properties of the GNS, but many experimental details and some details of the measurements and their description need to be added. While the results are promising, several key issues must be addressed to fully substantiate the claims and ensure the robustness of the methodology.

Question 1: In the abstract, the authors mention that the work results … in the highest GNS density and length. Similar, even higher lengths have been reported for example in Li et al., Commun Phys 1, 44 (2018). https://doi.org/10.1038/s42005-018-0043-2.

Question 2: The manuscript lacks detailed descriptions of the experimental detail. The duration of UV illumination has a significant impact on the adhesion of the functional tape and graphene, which will further influence the strain transfer efficiency and subsequent peeling/curling behavior. Providing these details such as the fabrication process of the UV tape and the illumination time is essential for the reproducibility of the method. Additionally, more discussions and characterizations about the influence of tape-graphene adhesion on the formation of GNSs should also be provided. Furthermore, after releasing the strain, an excess stimulus is required. The authors mentioned the use of an organic solvent, such as acetone. Does this solvent have any impact on the tape? For example, causing the tape to swell? It should be explained in detail.

Question 3: According to Figures. 1a, S2 and S3, after the tape was stretched, periodic linear cracks oriented perpendicular to the strain direction appeared on the graphene film. By comparing Figures S3a-c and d, it is evident that without external stimulation, the graphene did not curl into GNSs. In the manuscript, the authors primarily focus on analyzing the role of tensile stress, but they do not provide a detailed explanation of how subsequent stimuli, such as organic solvents, influence the scrolling of graphene. While stress is responsible for the formation of periodic cracks, it is the external stimuli that should be regarded as one of a key driving force behind the actual scrolling of graphene into nanoscrolls. The authors should provide a detailed discussion on this issue.

Question 4: As the authors regulated the adhesion of the tape by UV illumination and achieved the curling of graphene through strain-engineering and external stimuli, the potential for polymer residue from the tape on the graphene surface must be considered. This issue should be taken into consideration, ideally with supporting characterization data.

Question 5: The authors claim the ability to synthesize GNSs with controlled chirality (zigzag, armchair, chiral) by simply

adjusting the stretching direction of the tape/graphene stack (Lines 114-118, Fig. 1c-e). This claim is primarily inferred from the optical microscopy images (Fig. 1c-e) and the known relationship between crack orientation and strain direction. However, direct atomic-scale evidence linking the prescribed stretching direction to the resulting edge structure of the final GNS is lacking. It is recommended to employ techniques such as STEM with high resolution to directly visualize the edge atomic structure of individual scrolls synthesized under different strain orientations. This will provide crucial validation for the proposed control over GNS chirality.

Question 6: The SEM image (Figure 3) interpretation might be ambivalent (Line 184-192). We observe that a GNS like contrast is already present on the substrate. It is not clear, if this structure is displaced or if an already formed GNS continues to roll up.

Question 7: Is the description of the experimental observation during the GNS formation complete? A challenge in such processes is the competition between complete delamination/rolling and fracture into smaller fragments. To demonstrate a complete understanding of the process and establish a convincing rationale for the high yield of GNSs reported, it should be clarified what specific measures were taken to decisively favor the scrolling pathway over fracture.

Question 8: Please explain in more detail which in Figure S9, where the structure collapsed. The geometry is not clear form figure or text.

Question 9: In Figure 3f, we obtain quite consistently 3.7 Å interlayer spacing, not the reported 0.34 nm. This brings the lattice plane spacing of the GNS in this work into the range of the ones observed by others. And the strain driven part of the scrolling process might not be very different from the impact of strain in other GNS work. Also, the authors report that, similar to other works, the scrolling result is best in the presence of solving. Therefore, we believe that in the modelling, the pressure coming from surface tension must be considered as a second factor. Or, if there is only a non-specified pressure used in the system, the share between pressure coming from surface tension and from the straining procedure should be calculated (Line 196-197).

Question 10: line 218: “unstable...” . We believe that the strain might not be the only mechanism causing the rolling effect. A solvant is used and surface tension can contribute to the formation of a 3nm GNS.

Question 11: In the section of “Electrical characterization and intercalation”, the fabrication process of the device must be clearly outlined. where is Photolithography, where is electron beam deposition used?

Question 12: Is a hysteresis observed when reducing V(drain)? (Line 272-280)

Question 13: Line288-289, here, the current density needs to be mentioned, i.e. the diameter of the GNS needs to be reported.

Question 14: line 298 ff, EDX and EELS spectra should be shown.

Question 15: The terminology in some sections of the manuscript lacks precision. For example, “Raman and cross-sectional electron microscopy” (Line 36) should likely be specified as “Raman spectroscopy and cross-sectional transmission electron microscopy”. Additionally, the abbreviation “MD” is used without being defined. Do the authors use MM and MD for the same simulation technique used in this work? Please thoroughly review and revise the relevant descriptions throughout the manuscript to ensure terminological accuracy and professionalism.

Question 16: In literature, we found only deformation of graphene adhered to tape or polymer is in the range of 1-3 %, not the 5% reported here, see for example Guo et al, Adv Mat Interfaces, 10, 2201531, 2023. The 5% reported here seem extraordinarily high. The authors should explain, how they reach this very high value and prove that the graphene stays really adhered to the polymer up to a strain of 5% of the graphene sheet, i.e. not 5% strain of the tape. (Line 93-97)

Question 17: line 158: Did you only measure graphene thickness by RS, or did you, in addition measure height on graphene flakes transferred to for example Si substrates by AFM?

Reviewer #4

(Remarks to the Author)
I co-reviewed this manuscript with one of the reviewers who provided the listed reports. This is part of the Nature Communications initiative to facilitate training in peer review and to provide appropriate recognition for Early Career Researchers who co-review manuscripts.

Reviewer #5

(Remarks to the Author)
I co-reviewed this manuscript with one of the reviewers who provided the listed reports. This is part of the Nature Communications initiative to facilitate training in peer review and to provide appropriate recognition for Early Career Researchers who co-review manuscripts.

Version 1:

Reviewer comments:

Reviewer #1

(Remarks to the Author)
In the revised manuscript, the authors have provided additional experimental results to address the referees' comments. Overall, I am satisfied with the revisions and recommend publication of this excellent study in its present form.

Reviewer #2

(Remarks to the Author)
The authors have adequately addressed all the concerns raised in the previous review. The revised manuscript has supplemented sufficient experimental data, rationalized the discussion, and clarified ambiguous points. I believe the current version meets the requirements for publication, and I support accepting this manuscript.

Reviewer #3

(Remarks to the Author)

Reviewer #5

(Remarks to the Author)
I co-reviewed this manuscript with one of the reviewers who provided the listed reports. This is part of the Nature Communications initiative to facilitate training in peer review and to provide appropriate recognition for Early Career Researchers who co-review manuscripts.

Open Access This Peer Review File is licensed under a Creative Commons Attribution 4.0 International License, which permits use, sharing, adaptation, distribution and reproduction in any medium or format, as long as you give appropriate credit to the original author(s) and the source, provide a link to the Creative Commons license, and indicate if changes were made.
In cases where reviewers are anonymous, credit should be given to 'Anonymous Referee' and the source.
The images or other third party material in this Peer Review File are included in the article’s Creative Commons license, unless indicated otherwise in a credit line to the material. If material is not included in the article’s Creative Commons license and your intended use is not permitted by statutory regulation or exceeds the permitted use, you will need to obtain permission directly from the copyright holder.
To view a copy of this license, visit https://creativecommons.org/licenses/by/4.0/

Reviewer #1 (Remarks to the Author):

This work utilizes functional tape with ultraviolet (UV) light-tunable adhesion to achieve the large-scale synthesis of graphene nanoscrolls (GNSs) through strain regulation. This strain-assisted method is efficient and also enables the control of the scrolling angle (i.e., the chiral angle) by manipulating the stretching direction. The work promises to be a significant contribution to the field, but more comprehensive studies are needed prior to publication:

We acknowledge the reviewer for valuable comments on our manuscript and also for understanding of the importance of our original synthesis method of GNSs. Based on the reviewer's comments, we have significantly revised our manuscript and performed additional experiments to address the raised concerns.

1. The strain-regulation method using functional tape with UV-tunable adhesion allows control of the scrolling angle by varying the stretching direction. The main novelty of this method is the high yield and good control on the rolling direction. However, we did not observe sufficient demonstration on tuning the scrolling angles, particularly the chiral angles, analysis on the controllability of scroll angles etc. in the manuscript. Is the scrolling direction solely determined by the direction of the applied tensile strain?

This reviewer's comment is quite reasonable, and as pointed out, the optical microscope images of GNSs are not sufficient to claim the control of their chirality. Therefore, we have performed TEM and STEM measurements for three types of GNSs (zigzag, armchair, and chiral) and analyzed the chirality by fast Fourier transform (FFT) analysis. As shown in Fig. 1g-i and Supplementary Fig. 4, we confirmed the chirality-controlled nanoscroll formation. We consider this is an interesting finding, because it is widely acknowledged the difficulty to control the chirality for multiwalled carbon nanotubes, in which each wall has a different chirality. Thus, our method allows fabricating chirality-controlled GNSs, offering new opportunities to explore a novel type of 1D materials.

The scrolling direction is determined by the direction of the applied tensile strain, which generates well-aligned cracks in the 2D material that are perpendicular to the strain direction. These cracks subsequently act as the edges from which the scrolling initiates. In all our experiments, performed under several conditions (including use of different solvents to assist the scrolling, and applied strains), we consistently found that the crack orientation, and consequently the scrolling direction, are controlled by the applied strain.

New Fig. 1c,g-i:

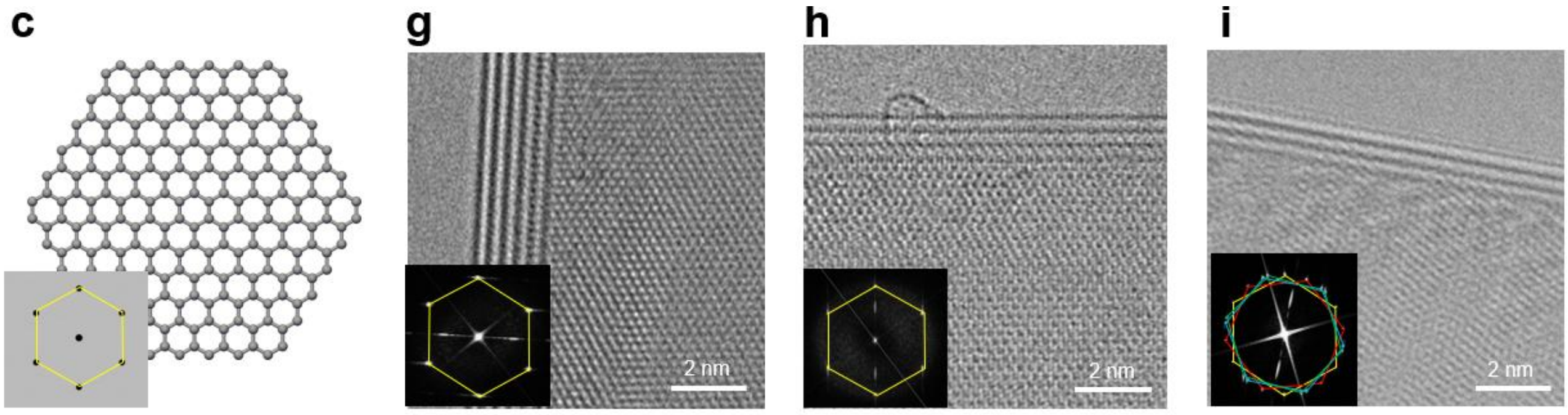

Fig. 1. c, Schematic atomic structure and corresponding simulated diffraction pattern (inset) of the hexagonal grain shown in **b. d-f**, GNSs made by stretching the UV tape/graphene stack along the directions indicated by the arrows. (**d**) zigzag GNSs, (**e**) armchair GNSs, and (**f**) chiral GNSs. **g-i,** Top-view high-resolution TEM images of the corresponding GNSs. Insets are the FFT patterns.

Pages 5-6 of the revised manuscript:

"To confirm the chirality-selective formation of nanoscrolls, the GNS samples were further characterized using high-resolution electron microscopes. Fig. 1g-i display the top-view transmission electron microscopy (TEM) images of the nanoscrolls, together with their corresponding FFT patterns shown in the insets (the corresponding high-resolution scanning transmission electron microscopy (STEM) images are displayed in Supplementary Fig 4). In Fig. 1g-i, we clearly observed the layered structure reflecting the rolling structure. The FFT patterns shown in the insets confirmed the formation of zigzag and armchair nanoscrolls, which are solely determined by the stretch directions. It is worth noting that it is difficult to grow chirality-controlled carbon nanotubes, while our unique tensile-based method allows to make zigzag and armchair scrolls in a highly controlled manner. In addition, due to the scrolling structure, all the rolls have the same chirality, which is completely different from a MWCNT in which each layer has different chiralities.[19] Chiral GNS exhibited interesting FFT patterns (inset of Fig. 1i); the number of the set of spots almost corresponding to the number of rolls. Although the origin of the multiple patterns is not clear, it may reflect a cone-like chiral nanoscroll structure. The possibility of the chirality control, including zigzag and armchair, is an advantage of our strain-assisted nanoscroll formation, which offers new opportunities to explore chiral-related phenomena."

2. Optical microscopy observations reveal non-uniform morphology of the graphene nanoscrolls (e.g., Fig. 1d). What causes this non-uniformity? Can the synthesis be optimized to yield higher-quality

GNSs?

Thank you for this crucial comment. To improve the quality of GNS arrays, such as density and structure, we have thoroughly investigated the mechanism of the scroll formation. The following are our attempts to improve the GNS quality during this revision:

(1) We have studied the actual strain applied to the UV tape by using a digital image correlation (DIC) method (new Fig. 2 and Supplementary Fig. 8), to improve our understanding of the tape behavior.

(2) The *in-situ* crack formation in graphene was studied while applying strain to the graphene/tape stack (new Supplementary Fig. 9)

(3) Raman spectra have been collected while applying the strain (new Supplementary Figs. 11, 12), allowing to follow the strain in the graphene before and after cracks formed.

(4) Effects of solvent and temperature on the scrolling were also studied (new Supplementary Fig. 13)

As a result of these investigations, we could increase the nanoscroll yield, as shown below (Fig. R1-1). To the best of our knowledge, our method achieves among the highest yields (density) and alignment compared with reports in previous literature (please refer to Figs. R1-2).

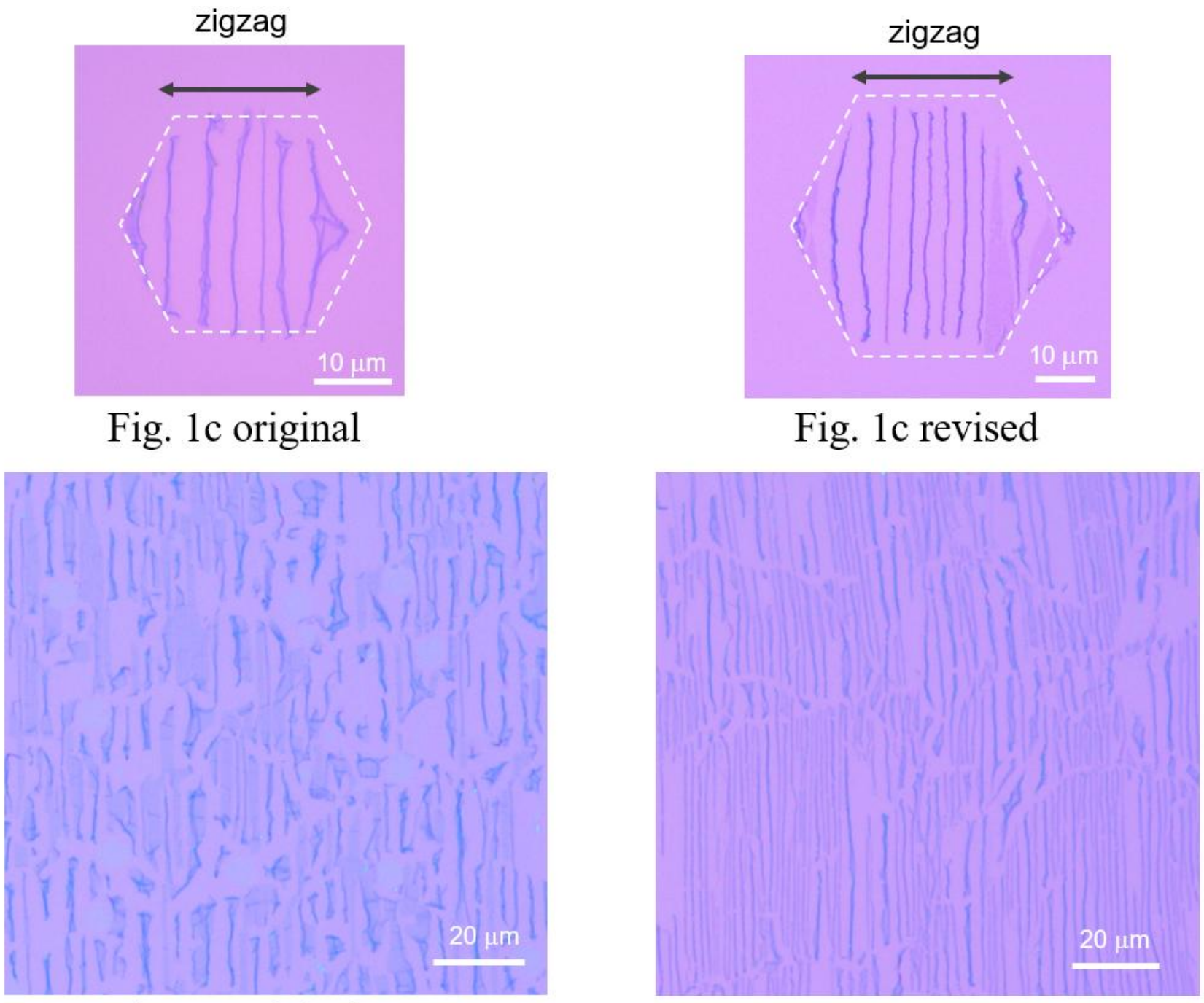

Fig. 1g original (zoom) Fig. 1g revised (zoom, now Fig. 1o)

Fig. R1-1 Comparison of the original and the revised GNS structures. The applied strain was increased from 5% to 10%, and a different solvent was used in the graphene release process.

[FIGURE REDACTED]

Fig. R1-2 GNSs structures reported in the previous literature. (a) GNSs synthesized on substrates, mainly on SiO_2. (b) GNSs synthesized in solvent (liquid dispersion). It can be seen that GNSs synthesized on substrates have a lower scroll density than those reported in our work (see Fig. R1-1). In addition, we have demonstrated the synthesis of GNS arrays for much larger areas than those of the previous literature, proving the high efficiency of our method. On the other hand, the liquid phase method gives entangled network of nanoscrolls, making bundles, without any specific alignment after depositing on a substrate.

New figure (Fig. 2):

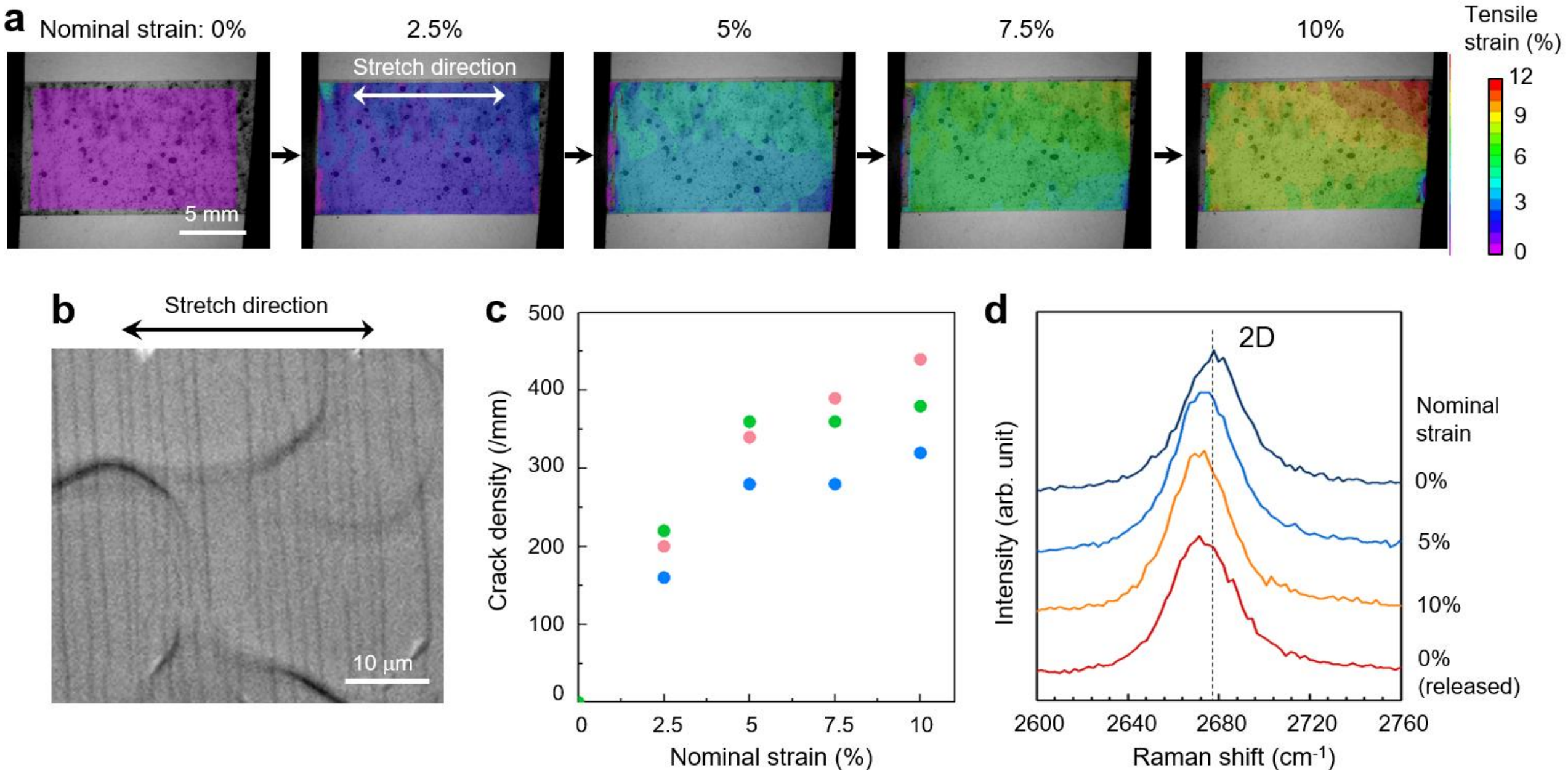

Fig. 2. *In-situ* measurement of tape deformation, crack formation, and Raman spectra. a, DIC analysis of the actual tensile strain applied to the graphene/tape stack. The nominal strain is calculated from the change of the tape length, and tensile strain was determined from the local changes of each position of the stack. **b**, Laser confocal microscope image of the graphene on the UV tape, captured while applying a nominal strain of 10%. **c**, Evolution of crack density of graphene with nominal tensile strain, measured at three different positions. **d**, *in-situ* Raman spectra measured while applying the strain, and after releasing the nominal strain applied to the stack.

New supplementary figure (Supplementary Fig. 8):

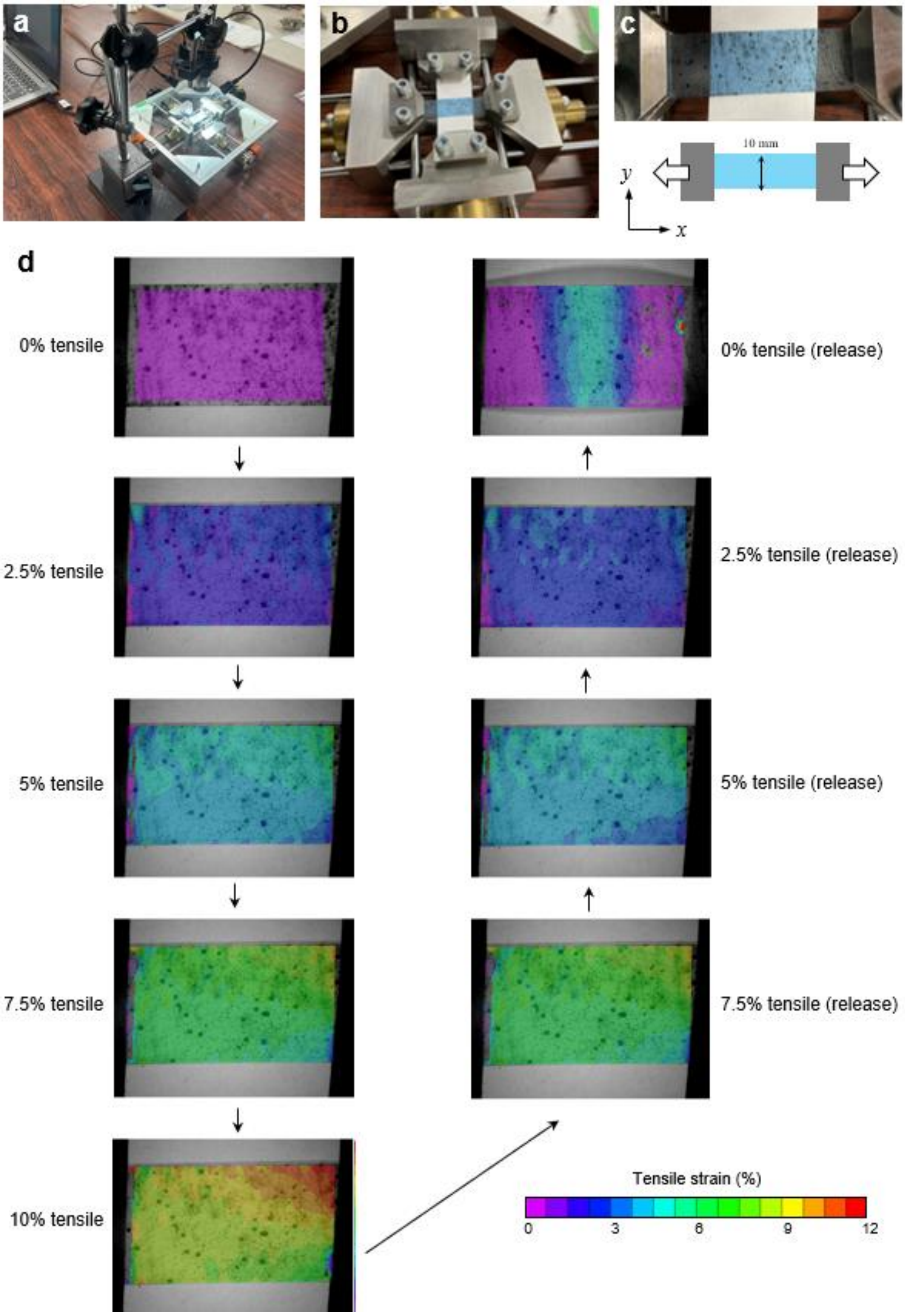

Supplementary Fig. 8. Experimental measurement of tensile strain applied in the UV tape measured by a digital image correlation method. a,b, Experimental setup used to measure the spatial distribution of applied strain. **c**, On the graphene/UV tape surface, a number of small black

particles were widely deposited, which were used to monitor the change of each particle position with respect to the applied strain using the mechanical jig. The stack was stretched along *x*-direction in the lower panel, while following the positions of particles. **d,** Evolution of tensile strain in the UV tape. We stretched the UV tape up to 10%, and then returned to the original position (0%). These images confirm that the applied tensile strain closely follows the calculated value from the extended length of the UV tape. However, when the tensile was increased to ~10%, inhomogeneities were observed due to the deformation of the UV tape. Note that applying strain up to 10% induces plastic deformation in the tape, resulting in the buckling after fully releasing the strain. Thus, the strain distribution shown in "0% tensile (release)" does not represent the actual remaining strain. The continuous images of the strain distribution can be found in Supplementary video 1.

New supplementary figure (Supplementary Fig. 9):

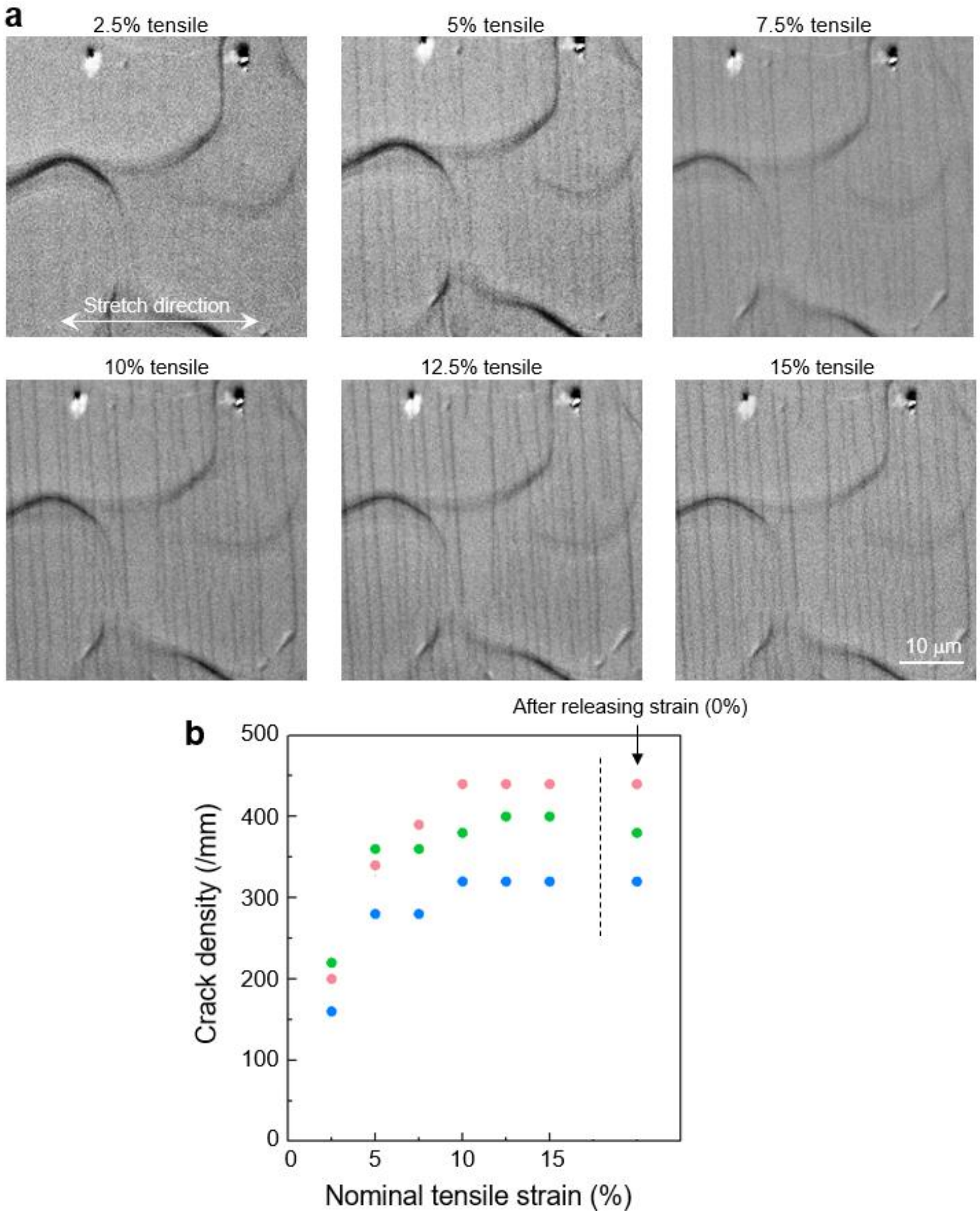

Supplementary Fig. 9. Crack formation while applying strain to the UV tape. a, Confocal laser microscope images of monolayer graphene on the UV tape captured under different applied strains

(the strain values are the nominal values calculated from the change of the tape length). To monitor the crack formation, we captured images at identical positions. It is clearly seen that cracks are formed by extending the UV tape, with the number of cracks increasing with strain. **b**, Crack density plotted against the applied tensile strain. The plot shows three different positions of the stack, indicating a similar trend regardless of the area. The crack density practically saturates at ~10% strain.

New supplementary figure (Supplementary Fig. 11):

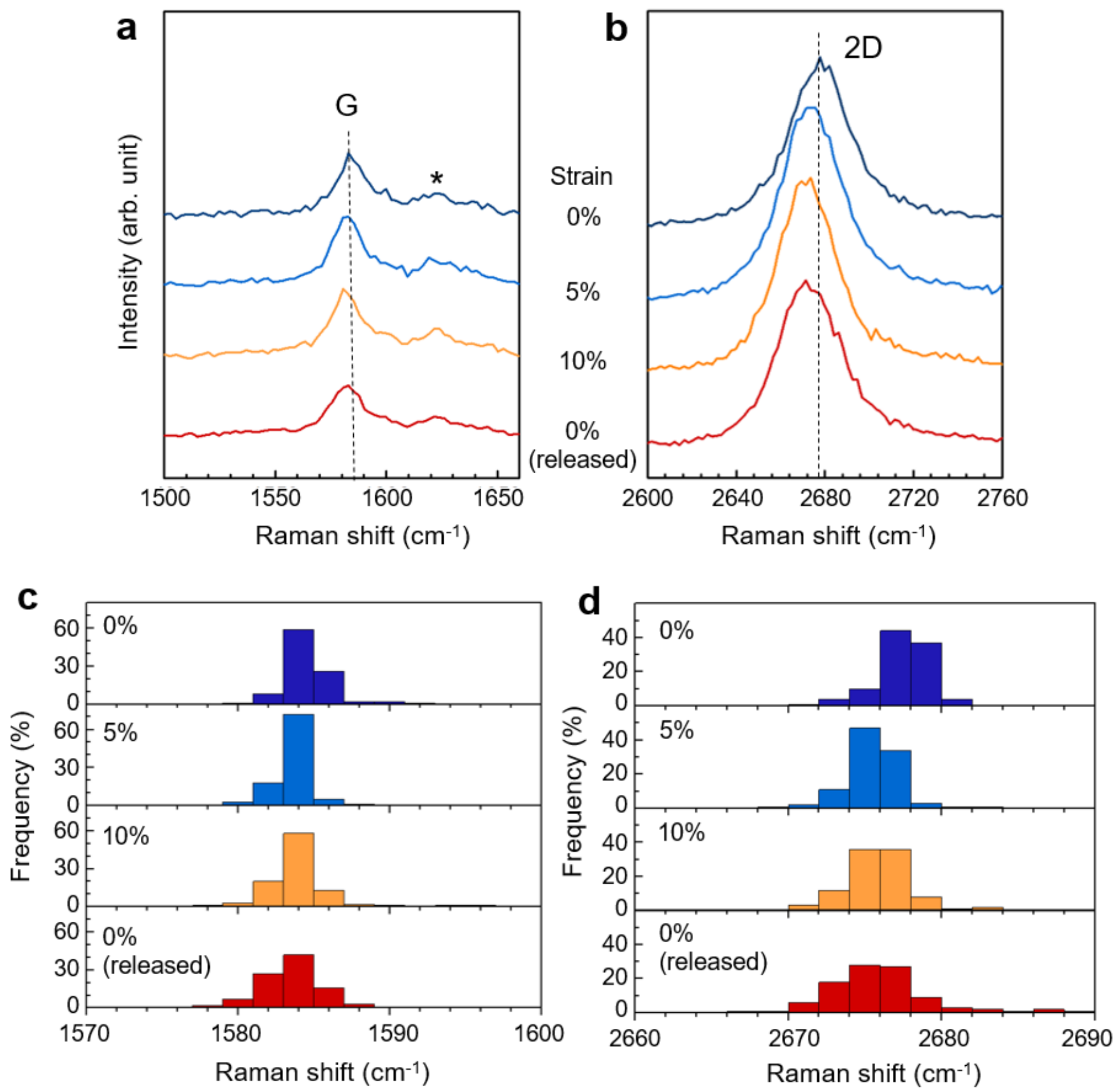

Supplementary Fig. 11. Raman spectra of graphene on the strained UV tape. a,b, Raman spectra of monolayer graphene measured while applying tensile strain to the graphene/tape stack: G band (**a**) and 2D band (**b**). The strain values are nominal values calculated from changes of the tape length. The bottom spectra (red) were taken after releasing the strain of the tape. Asterisk in **a** represents a peak from the adhesive layer of the tape. **c,d,** Distributions of peak positions of G band (**c**) and 2D band (**d**).

New supplementary figure (Supplementary Fig. 12):

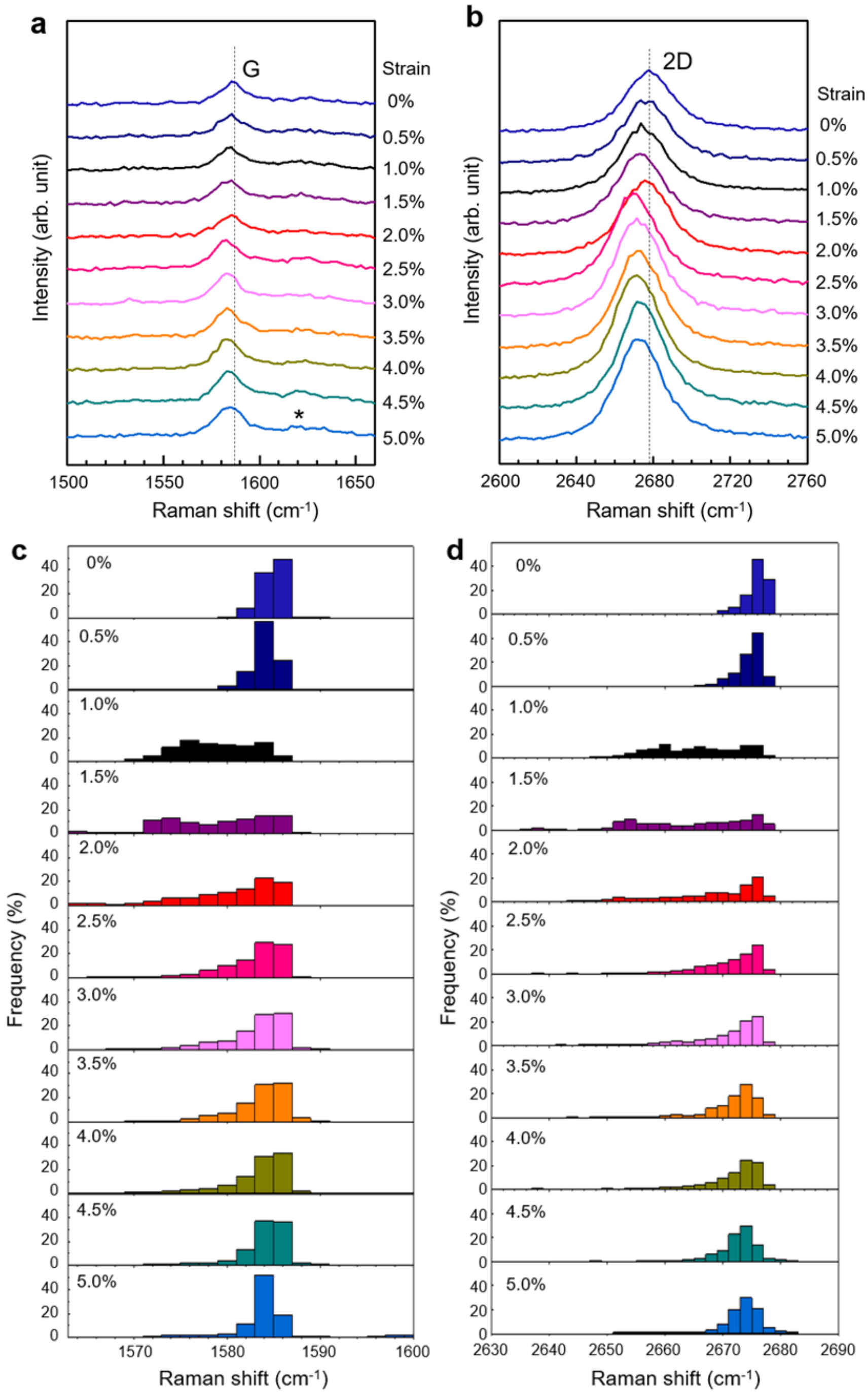

Supplementary Fig. 12. Raman spectra of graphene on the UV tape under low strain loads. a,b, Raman spectra of monolayer graphene measured while applying tensile strain (up to 5%) to the graphene/tape stack: G band (**a**) and 2D band (**b**). The strain values are the nominal values applied to the UV tape. Asterisk in (**a**) represents a peak from the tape. **c,d,** Distributions of the positions of G band (**c**) and 2D band (**d**).

Significant downshifts were observed of both G and 2D bands in the range of 1.0~1.5% strain (Supplementary Fig. 12c,d), indicating the introduction of substantial tensile strain in the graphene. However, when the graphene/UV tape stack was extended to 2.0%, the fraction of spectra exhibiting downshifted G and 2D bands decreased. As the applied strain was increased to 5%, both the 2D and G bands gradually shifted back to higher wavenumbers, as evident in the decrease of the low-wavenumber tails of the histograms. This behavior suggests the onset of crack formation at approximately 2.0% strain, followed by the progressive formation of additional cracks with increasing applied strain, which gradually relieves the tensile strain stored in the graphene (but the overall peak distributions are slightly shifted to lower wavenumbers as compared with those before applying strain). In addition, an increase of the 2D band intensity was also observed with increasing the strain, suggesting the slight decoupling from the tape surface.

New supplementary figure (Supplementary Fig. 13):

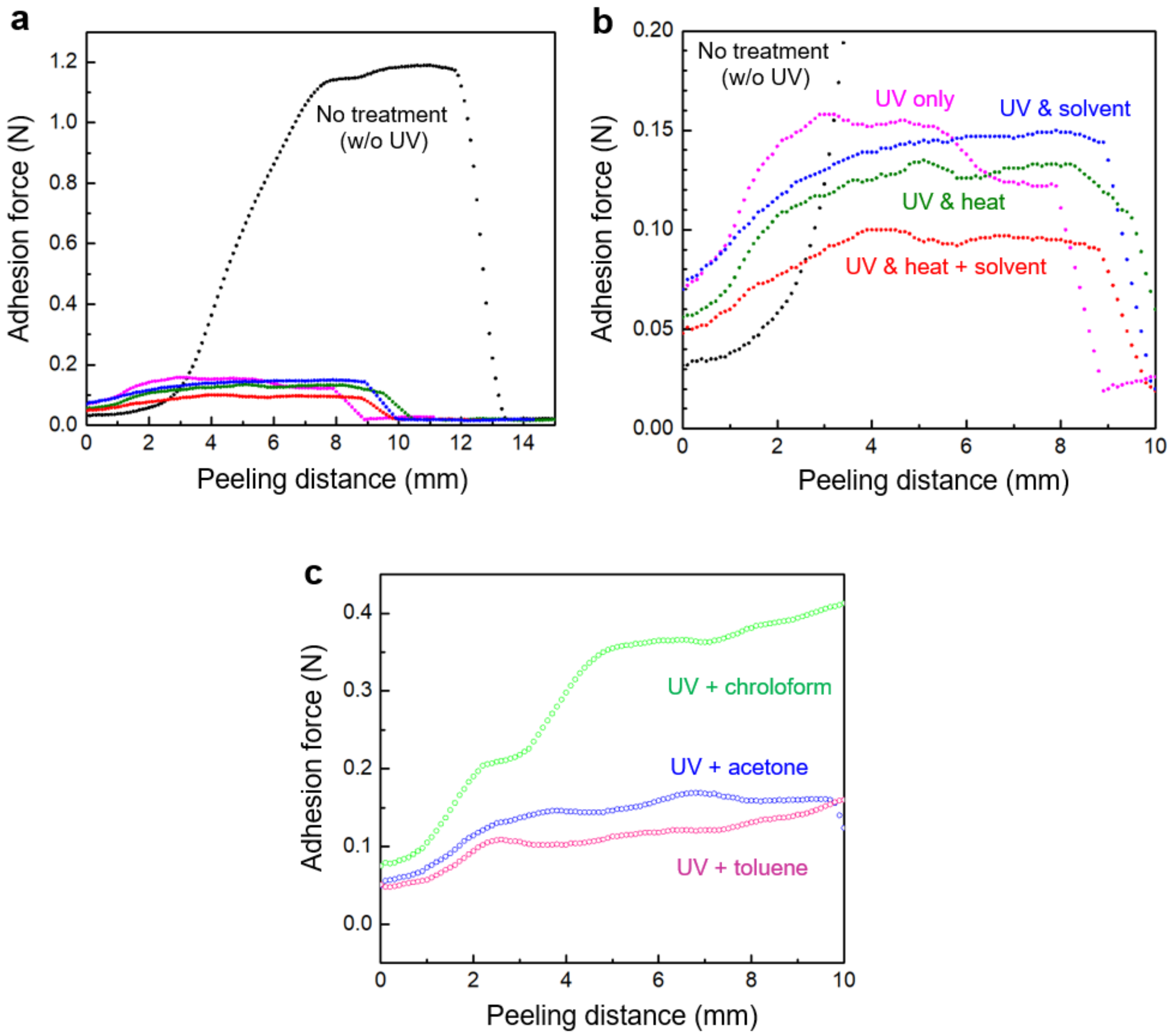

Supplementary Fig. 13. Influence of the post-UV treatment on the adhesion force of the UV tape

a, Comparison of adhesion force of UV tapes measured after different treatments. The adhesion force

was measured by removing the treated UV tape from a bare SiO_2/Si substrate (10 mm width). No UV illumination (black) maintained strong adhesion force. **b,** After UV illumination the adhesion significantly decreased (pink). The drop of solvent (acetone) slightly decreased the adhesion force (blue), but the heat treatment (80 ºC) was more effective to decrease the adhesion force (green). Interestingly, combining the heat treatment and solvent showed the lowest adhesion force (red). **c,** Solvent dependence of the adhesion force (without heat treatment). To synthesize GNSs from the strained graphene, we used toluene and acetone, which are more effective than chloroform.

As the combination of heat and solvent reduces the adhesion force, the solvent can also contribute to effective detachment of graphene from the tape surface. In addition, intercalation of solvent to the graphene-tape interface can assist the formation of GNSs.

3. The electronic properties of graphene scrolls need more comprehensive study. Except sustain higher current density, what is the advantage of graphene scrolls? The measurements in Fig. 5 seems indicate the graphene scroll show neglectable gate effect, thus what kind of application is expected in electronics?

As mentioned in the original manuscript, the high current density is one of the advantages of GNSs. This one-dimensional high electrical conductor can find applications, such as interconnect application, like that demonstrated with intercalated multilayer graphite (Katagiri, M. et al. Intercalation doping of narrow multilayer graphene interconnects with sub-100 nm widths. *Jpn. J. Appl. Phys.* **56**, 07KD01 (2017)).

In addition to this application, we have demonstrated two new applications in the revised manuscript; nanoscale memory and photodetectors. As shown in new Fig. 6b, GNSs can exhibit hysteresis in the transfer curves even under vacuum, due to trapped charged species inside the nanoscroll. This differs from graphene FETs (Supplementary Fig. 22). Such hysteresis can be used for miniaturized memory devices. We therefore demonstrate the memory operation in Fig. 6c. The ability to store molecules and ions offers new possibilities, such as improved electrical conductivity induced by the molecular intercalation (Fig. 6e).

With graphene-MoS_2 heteronanoscrolls, we can expect improved optical response due to the excellent electrical conductivity of graphene and high optical absorbance of the MoS_2 rolls. Thus, we fabricated the heteronanoscroll devices, demonstrating their clear photoresponse, as shown in new figures (Fig. 6f, Supplementary Fig. 26). This optical response was absent in GNS-FETs

New figure (Figs, 6b, 6c, and 6f are new data):

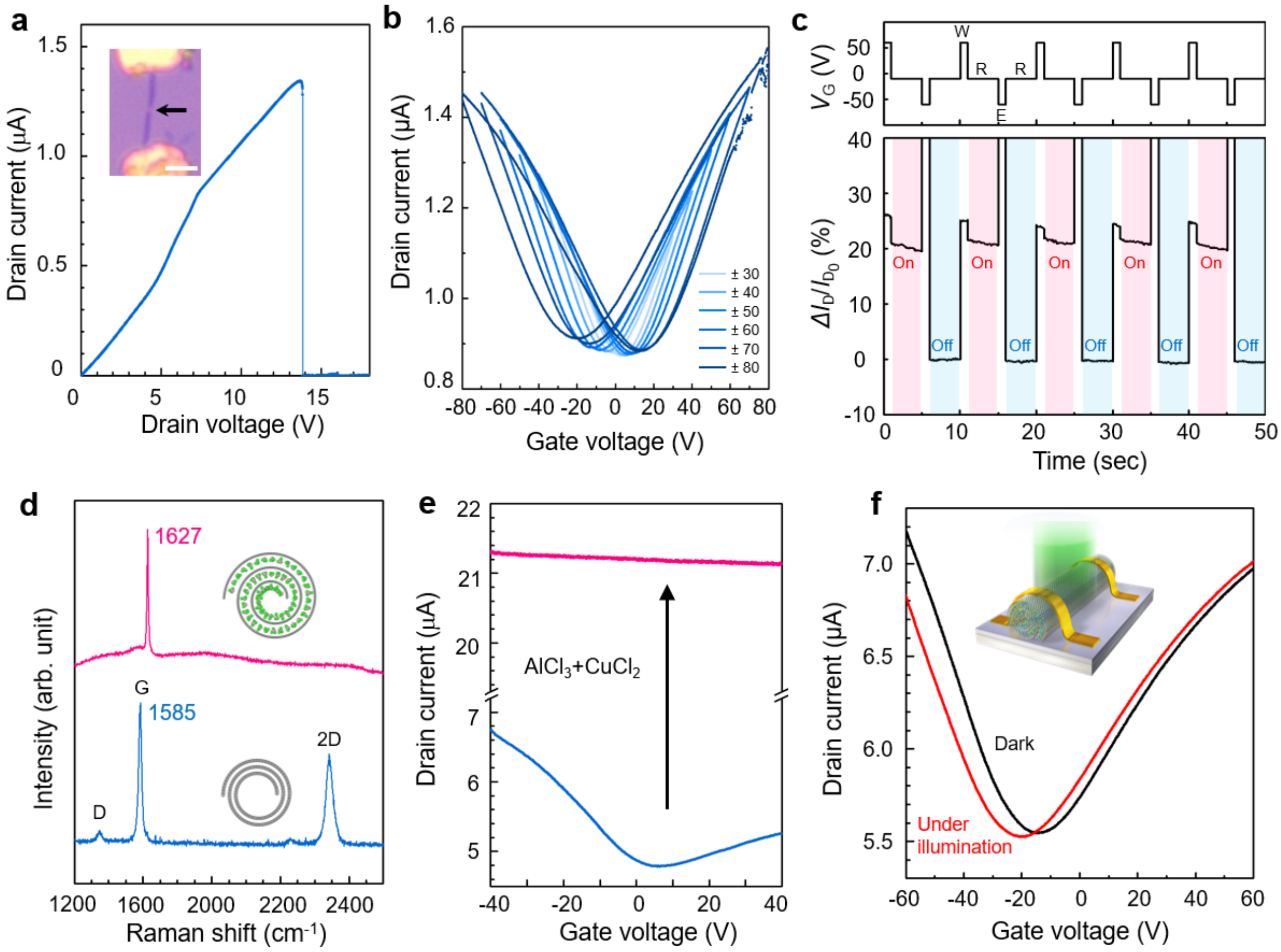

Fig. 6. Electrical transport properties and applications of GNSs, intercalated GNS, and heteronanoscrolls. a, Breakdown behavior of a GNS channel. Inset shows the optical image after the breakdown (scale bar: 2 μm). The arrow indicates the position where the breakdown occurred. **b,** Transfer curves of a GNS-FET measured at six different sweep gate voltage ranges (from ±80 to ±30 V with a 10 V step) in vacuum. **c,** Memory effect of a GNS-FET. The upper panel shows the profile of the applied gate voltage, and the lower panel is the change of the drain current showing the memory effect due to the large hysteresis. **d,** Raman spectra of a pristine GNS (blue) and an $AlCl_3$-$CuCl_2$ intercalated GNS (pink). Insets show the schematic models. **e,** Transfer curves of a GNS measured before (blue) and after the intercalation (pink). **f,** Light response of the co-nanoscroll of MoS_2 and graphene, showing a light-induced shift of the transfer characteristics toward negative gate voltages. Inset is a schematic of a co-nanoscroll under illumination.

New supplementary figure (Supplementary Fig. 22):

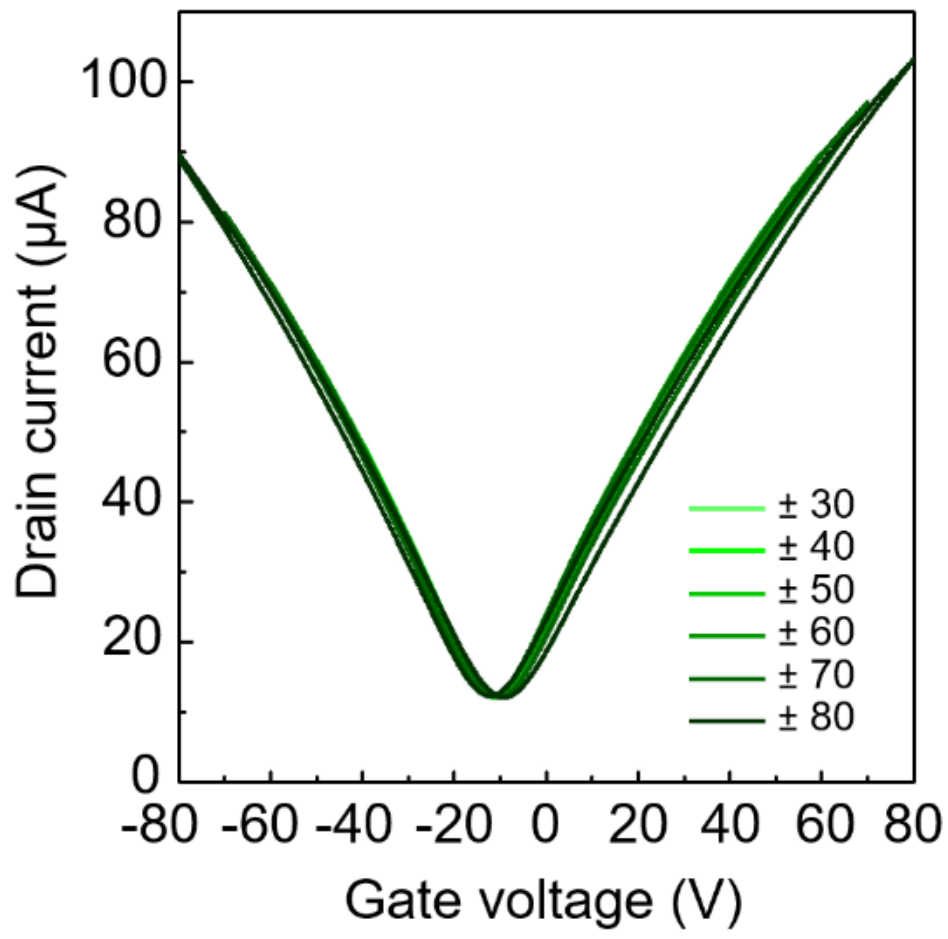

Supplementary Fig. 22. Negligible hysteresis in a graphene-FET. Transfer curves of a graphene-FET measured at six different sweep gate voltage ranges (from ±80 to ±30 V with a 10 V step). In contrast to the GNS-FET (shown in Fig. 6b), a graphene sheet did not show apparent hysteresis in the transfer curves.

New supplementary figure (Supplementary Fig. 25):

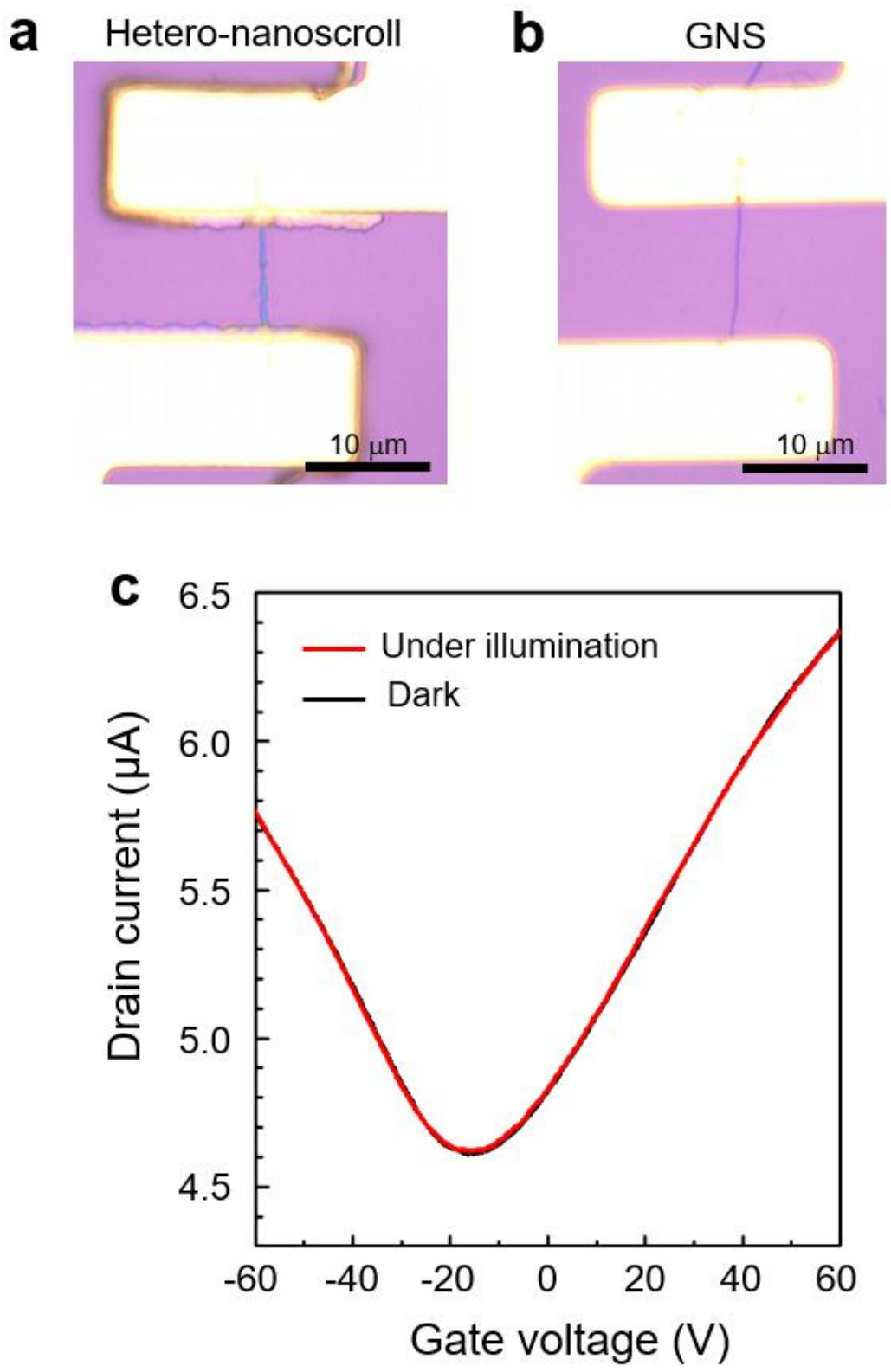

Supplementary Fig. 25. Optical images of heteronanoscroll and GNS devices and optical response. Optical micrographs of a heteronanoscroll (**a**) and GNS (**b**) FETs. It can be seen that the

heteronanoscroll has stronger optical contrast than GNS due to the presence of MoS_2. **c** Transfer curve of the GNS-FET in dark (black) and under 532 nm illumination (red) at V_D=100 mV. No photoresponse was observed in the GNS-FET.

Pages 16-17 of the revised manuscript:

"We further investigated the carrier transport properties of GNSs by fabricating bottom-gated field-effect transistors (FETs). Figure 6b shows transfer curves of the GNS-FET device measured in vacuum for different sweep ranges of the gate voltage. The GNS-FET exhibited a clear hysteresis that increases with the sweep gate voltage. The hysteresis was almost independent of the applied drain voltage, as shown in Supplementary Fig. 21, which is consistent with gate-controlled charge trapping processes. Together with the negligible hysteresis in the non-scrolled graphene FET (Supplementary Fig. 22), this suggests that the dominant charge trapping is associated with the structure of the GNSs, likely due to water and/or oxygen molecules trapped within the spaces between turns of the scroll. This result is consistent with the previous study reporting large hysteresis in MoS_2 nanoscroll devices, implying that the nanoscroll structure is suitable to store charges owing to the unique open and flexible structure.[48]

The substantial hysteresis observed in the GNS devices can be exploited for nanoscale memory applications. Figure 6c shows the change in drain current measured while the gate voltage was varied sequentially. Here, the write (W) and erase (E) pulses were set 60 and -60 V, respectively, while read voltage (R) was -10 V. The high-resistance state (HRS) and low-resistance state (LRS) can be stably maintained by input gate voltage. Therefore, the large and reproducible hysteresis in the GNS device enabled a repeatable memory effect. Further optimization of the trapped species within the GNS may provide a route to enhancing the memory performance of these devices."

Page 18 of the revised manuscript:

"The MoS_2-graphene heteronanoscrolls can be potentially used as a photosensor, combining the high optical absorbance of MoS_2 and the high carrier mobility of graphene, with the scroll geometry also expected to enhance light absorption.[51,52] Thus, we fabricated FETs using co-scrolls of graphene and MoS_2, as illustrated in Fig. 6f inset. The co-scroll FET exhibited clear photo-switching behavior, with a substantial shift of the transfer characteristics toward negative gate voltage upon visible light illumination (532 nm wavelength), corresponding to a decrease in the drain current in the hole-doped regime (Fig. 6f). On the other hand, the graphene-FET did not exhibit such light-induced current modulation (Supplementary Fig. 25). The observed light-induced current decrease in the GNS-FET can be explained by the photo-induced electron transfer from MoS_2 to graphene, as reported

previously for a vertically stacked MoS_2/graphene heterostructure.[35] The modulation of the drain current (~5%) is larger than the previously reported heterostructure (~2%),[35] owing to the scroll structure having a larger cross-section against the illuminated light. Stable optical response was observed over repeated measurement cycles (Supplementary Fig. 26). Overall, the unique nanoscroll structure of graphene and its heterostructures, including co-scroll and core-shell scrolls, offer a unique platform to derive new electronic and optoelectronic applications."

New reference:
(48) Qiao, S. et al. One-dimensional MoS_2 nanoscrolls as miniaturized memories. *Nano Lett.* **24**, 4498 (2024).

4. Do the low-frequency Raman breathing modes (LBM) of the graphene nanoscrolls exhibit differences depending on the scrolling angle and diameter?

We think that the observed low frequency Raman originates in the layer breathing modes (LB modes), which correspond to the phonons of the graphene moving along the normal direction of the layers (Yang S. et al. *ACS Appl. Mater. Interfaces* **17**, 14415 (2025)). We would like to note that these LB modes are observed even in multilayer graphene sheets (Lin, M. L. et al. *J. Raman Spec*. **49**, 19 (2018), Wu, J.-B. et al. *Carbon* **110**, 225 (2016)).

These LB modes are different from the so-called radial breathing modes (RBMs) widely observed in carbon nanotubes, which strongly depend on diameter and chirality. As our GNSs have a relatively larger inner diameter (TEM images in Fig. 3m,n), we think that the low frequency Raman peaks originate in the LB modes. In principle the frequencies of the LBMs can also be influenced by the diameter and scroll angle of the scroll. However, current literature indicates that LBM frequencies are mainly governed by the strength of the interlayer coupling and the number of interacting layers (for instance Wu, J.-B. et al *ACS Nano* **9**, 7440 (2015)), although a systematic study of the dependence on the scrolling angle or diameter has not been established experimentally. Our experiments also did not show clear evidence of any dependence, although it cannot be completely ruled out. The observation of the Raman LB mode peak hints to graphene forming a scroll structure with many interacting graphene turns.

Pages 10-11 of the revised manuscript:
"This LBM originates in the lattice vibration of graphene sheets in the out-of-plane direction, being also characteristic of multilayer graphene.[36,37] Therefore, the observation of the LBM peaks in the

GNSs indicates a tightly wound structure, consistent with the observed low I_{2D}/I_G ratio and broadened 2D band (Fig. 3c,g). As inner diameters of GNSs are relatively large (Fig. 3m,n), the observed low frequency Raman peaks do not seem to originate in the radial breathing modes (RBMs) which are well known for carbon nanotubes with diameters usually smaller than 2 nm.[38,39]"

New references:

(38) Rao, A. M. et al. Diameter-selective Raman scattering from vibrational modes in carbon nanotubes. *Science* **275**, 187 (1997).

(39) Ago, H. et al. Growth of double-wall carbon nanotubes with diameter-controlled iron oxide nanoparticles supported on MgO. *Chem. Phys. Lett.* **391**, 308 (2004).

5. Can the preparation of graphene-based heteronanoscrolls maintain the similarly high efficiency demonstrated for pure GNSs? Could corresponding characterization data (e.g., optical microscopy) be provided?

As the reviewer commented, we did not show the optical microscope images in our original manuscript. In order to address this reviewer's concern, we have added the optical image of the MoS_2-graphene heteronanoscrolls in Fig. 5b. We have also added more images in new Supplementary Fig. 17. As can be seen in Fig. 17c, bright contrast highlighted by the black arrows corresponds to MoS_2. Therefore, Fig. 5b and Supplementary Fig. 17a,b indicate a high yield of the heteronanoscrolls .

New figure (Fig. 5b):

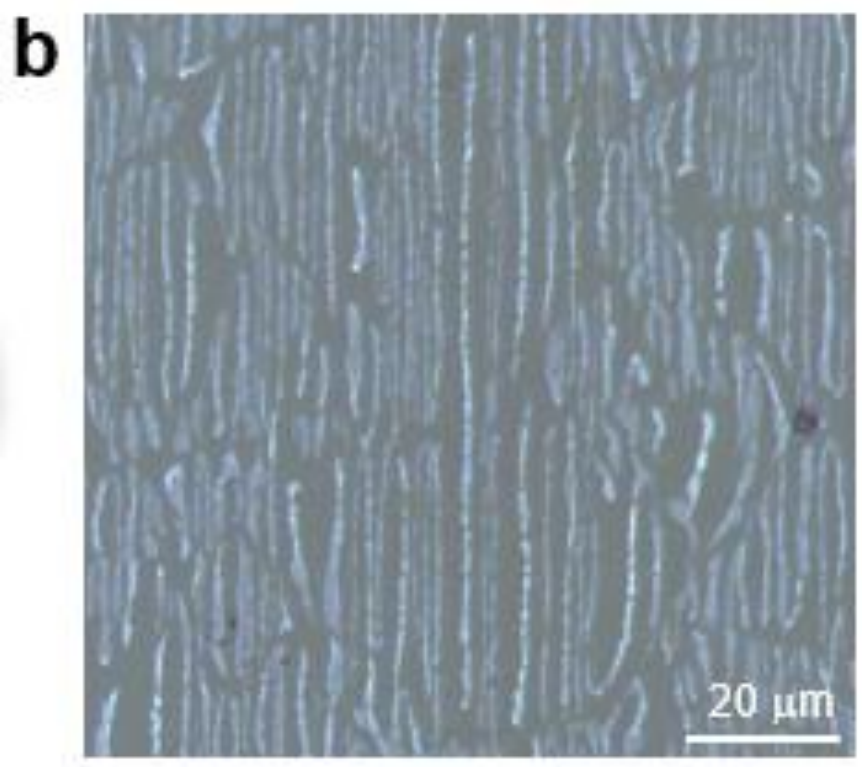

b, Confocal laser microscope image of the aligned co-nanoscrolls.

New supplementary figure (Supplementary Fig. 17):

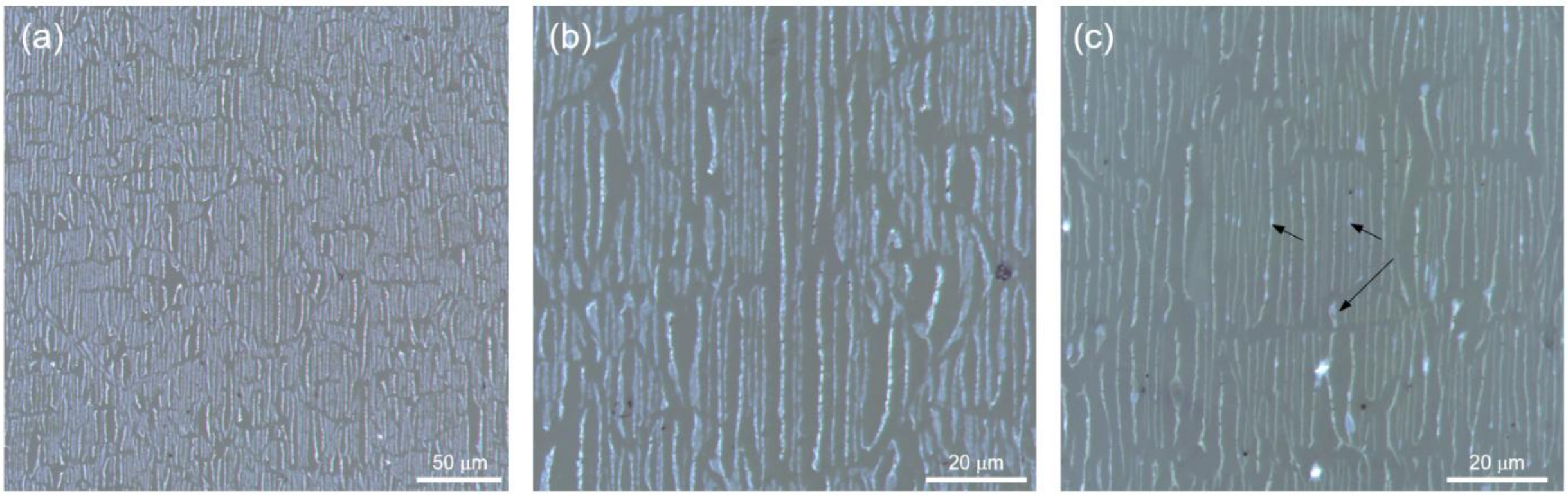

Supplementary Fig. 17. Optical images of MoS_2-graphene co-nanoscrolls. a,b, Most of nanoscrolls consist of MoS_2 and graphene. **c,** MoS_2-graphene heteronanoscrolls using MoS_2 with low coverage. Bright contrast indicated by the arrows show the presence of MoS_2.

6. Does the intercalation with metal ions alter the band structure of the graphene scrolls?

In the case of graphite, the intercalation of alkaline metals, such as K, increases the interlayer distance from 3.35 Å to 5.4 Å. This results in the decoupling of neighboring graphene layers. Accordingly, decoupling also occurs for graphene nanoscrolls, and thus it can be regarded as a quasi-free-standing monolayer graphene sheet having a loosely curved structure. The intercalation of alkali ions donates electrons to the conduction band of GNS. Therefore, we think that the electronic band structure of K-intercalated GNS becomes similar to electron-doped monolayer graphene having one-dimensional structure.

During the intercalation of metal chloride molecules, as shown in Fig. 6c,d ($AlCl_3$+$CuCl_2$ intercalation), electrons are withdrawn from graphene while also expanding the inter-layer distance.

Page 17 of the revised manuscript:

"In the case of bulk graphite, the intercalation of metal chlorides, increases the interlayer distance from 3.35 Å to 9~10 Å,[49] leading to the decoupling of adjacent graphene layers. This can also occur to the intercalated GNSs, partly decoupling the van der Waals interaction between neighboring rolls. At the same time, due to hole doping from the metal chlorides to the GNS, its electrical conductivity dramatically increased, as shown in Fig. 6e."

Reviewer #2 (Remarks to the Author):

This manuscript reports on a large-area synthesis of dense graphene nanoscrolls (GNSs) with controlled scroll direction by utilizing a functional tape, whose adhesive strength can be tuned with ultraviolet light. The authors demonstrate that stretching the tape applies tensile strain to graphene, resulting in the formation of vertically oriented GNSs during peeling. The ultrathin structure of graphene is difficult to manipulate, while the nanoscroll structure is beneficial for metal chloride and alkali metal intercalation, enhancing conductivity. This method is also extended to the preparation of GNS/MoS_2 heterostructures, providing a new approach for one-dimensional heterostructures. However, several critical aspects require clarification to fully substantiate the claims and solidify the mechanistic understanding of the process. Specifically, many of the produced scrolls exhibit twisted or wavy morphologies rather than being perfectly rectilinear, and their winding tightness appears to vary significantly. These are important structural inconsistencies that are likely to impact the intrinsic properties of the GNSs and necessitate further investigation. Also, graphene nanoscroll with certain scroll direction have been achieved by Wenping Hu et al, so the novety is also a question. Also with certain Given that these fundamental issues regarding structural uniformity and formation mechanism remain unresolved, I cannot recommend publication in Nature Communications in its current form.

We acknowledge the reviewer for the many crucial comments and suggestions. About the uniformity and quality of our GNSs, we have optimized the synthesis process mainly by studying the effects of strain and solvent, which will be described below. The reviewer raised a concern about the novelty of our method by referring to the recent paper recently by Prof. Wenping Hu (*Nat. Mater.* **24**, 377 (2025)). Their approach involves dipping the graphene/SiO_2 substrate into solvent to induce the scrolling by intercalating solvent between graphene and the substrate. Optical images of their nanoscrolls are reproduced in Figs. R2-1 and R2-2. While this paper represents an important advancement in the field of graphene nanoscroll synthesis, it does not provide a complete control of the yield (density), orientation, and spatial uniformity of the resulting nanoscrolls. In contrast, our approach enables the formation of highly aligned graphene nanoscroll arrays with high spatial uniformity and controllability over the scrolling direction. The scrolling direction is predetermined by the strain-induced crack orientation, enabling deterministic alignment of the nanoscrolls. Therefore, our method represents a distinct and complementary approach beyond previously reported solvent-induced scrolling methods, providing a new route toward the controlled synthesis of nanoscrolls. We believe that these advances represent a significant contribution to the field and make our work suitable for publication in *Nature Communications.*

[FIGURE REDACTED]

Fig. R2-1. Optical images taken from Fig. 1 of *Nat. Mater.* **24**, 377 (2025) by Prof. Wenping Hu. All scale bars are 10 μm.

[FIGURE REDACTED]

Fig. R2-2. Optical images taken from Fig. 2 of *Nat. Mater.* **24**, 377 (2025). All scale bars are 10 μm.

1. The authors claim that tensile strain is uniformly applied to graphene across the tape surface, leading to aligned GNS arrays. However, no quantitative characterization of strain distribution is provided. For large-area graphene films, strain gradient is inevitable due to edge effects and uneven adhesion between the UV tape and graphene. The paper only mentions periodic linear cracks perpendicular to the strain direction but lacks statistical data on crack density, width, and spacing, as well as their correlation with GNS density and alignment degree. It is recommended to supplement micro-Raman mapping of strain distribution (based on G-band shift) across the large-area graphene, and establish quantitative relationships between strain magnitude, crack parameters, and GNS structural characteristics (density, length, chiral angle).

We appreciate the reviewer for this essential comment, which raises very important questions. In the original manuscript we did not pay particular attention to the strain during stretching the graphene/tape stack, which certainly influence the structure and yield of final GNS arrays, as the reviewer commented. Based on this reviewer's comment, we systematically investigated the spatial distribution of strain and crack formation induced by extending the graphene/tape stack.

1. Spatial distribution of applied stain

First, we measured the spatial distribution of strain using a digital image correlation (DIC) method. For this, many small particles are dispersed on the surface of the stack, with their positions being tracked while mechanically extending the graphene/tape stack. The result has been newly added as Fig. 2 and Supplementary Fig. 8. In addition, a movie showing the process has been also created (new Supplementary video 1).

New figure (Fig. 2):

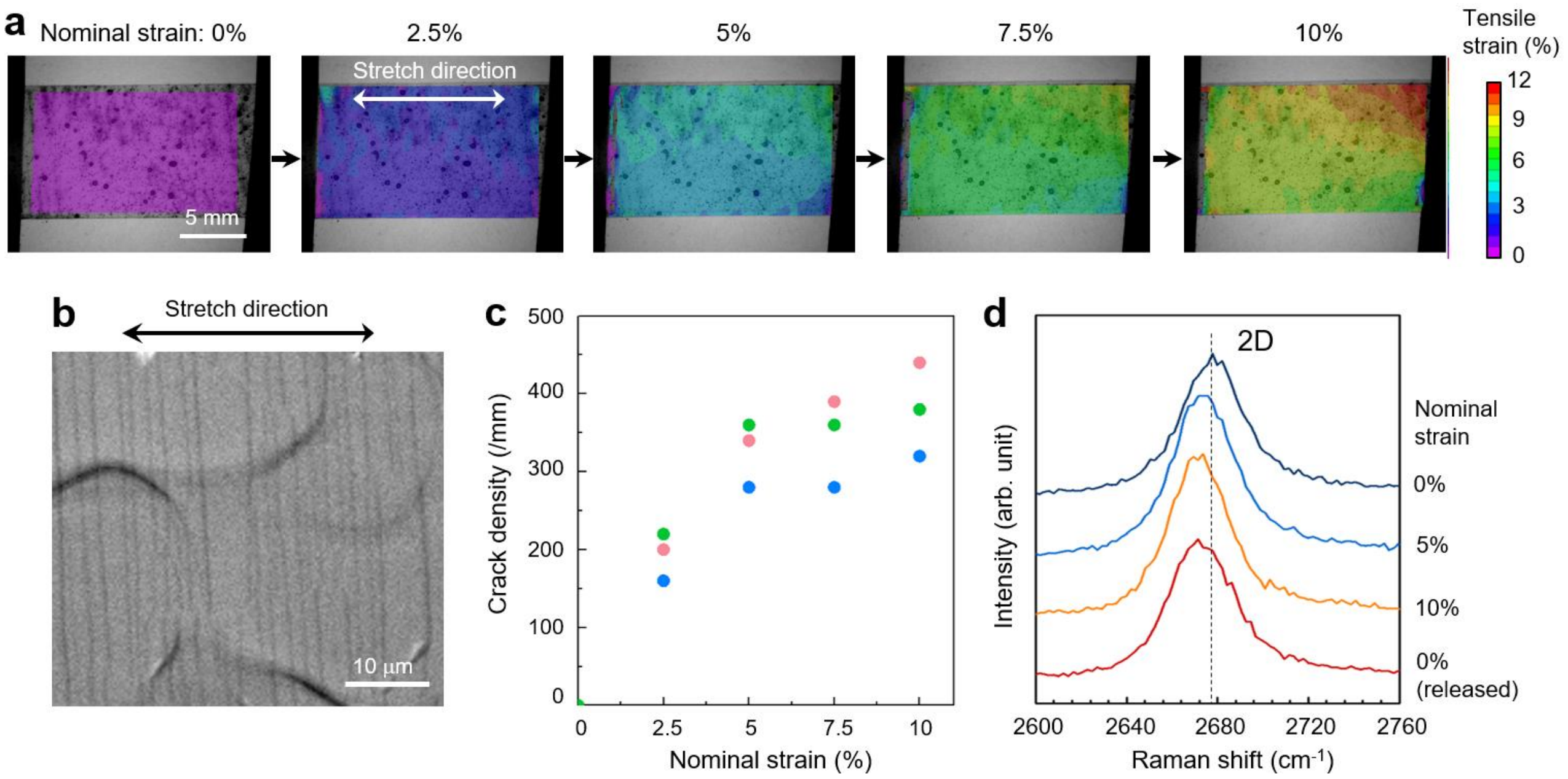

Fig. 2. *In-situ* measurement of tape deformation, crack formation, and Raman spectra. a, DIC analysis of the actual tensile strain applied to the graphene/tape stack. The nominal strain is calculated from the change of the tape length, and tensile strain was determined from the local changes of each position of the stack. **b**, Laser confocal microscope image of the graphene on the UV tape, captured while applying a nominal strain of 10%. **c**, Evolution of crack density of graphene with nominal tensile strain, measured at three different positions. **d**, *in-situ* Raman spectra measured while applying the strain, and after releasing the nominal strain applied to the stack.

New supplementary figure (Supplementary Fig. 8):

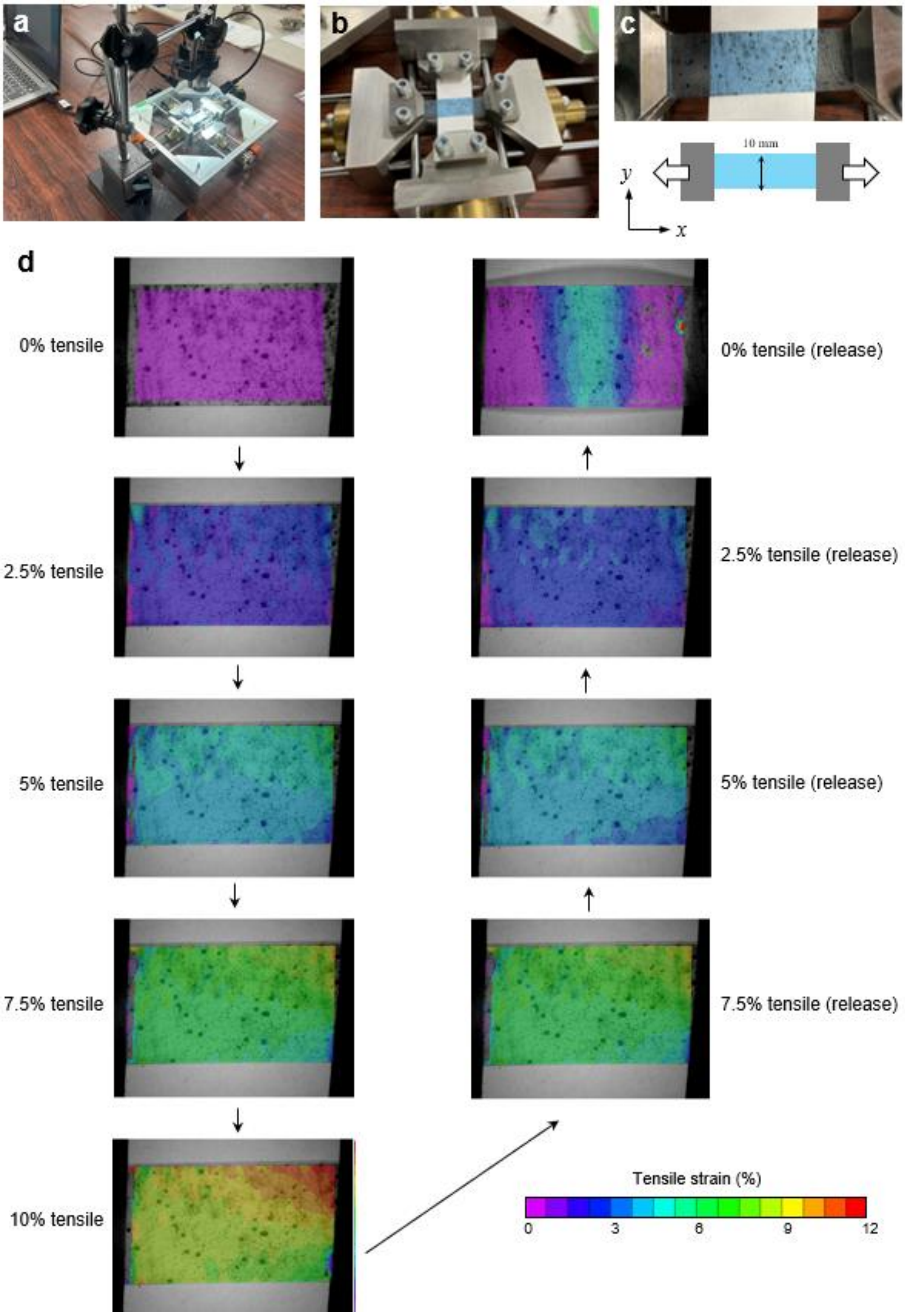

Supplementary Fig. 8. Experimental measurement of tensile strain applied in the UV tape measured by a digital image correlation method. a,b, Experimental setup used to measure the spatial distribution of applied strain. **c**, On the graphene/UV tape surface, a number of small black

particles were widely deposited, which were used to monitor the change of each particle position with respect to the applied strain using the mechanical jig. The stack was stretched along *x*-direction in the lower panel, while following the positions of particles. **d,** Evolution of tensile strain in the UV tape. We stretched the UV tape up to 10%, and then returned to the original position (0%). These images confirm that the applied tensile strain closely follows the calculated value from the extended length of the UV tape. However, when the tensile was increased to ~10%, inhomogeneities were observed due to the deformation of the UV tape. Note that applying strain up to 10% induces plastic deformation in the tape, resulting in the buckling after fully releasing the strain. Thus, the strain distribution shown in "0% tensile (release)" does not represent the actual remaining strain. The continuous images of the strain distribution can be found in Supplementary video 1.

As can be seen in Supplementary Fig. 2, we confirmed that relatively uniform strain was applied to the stack. The measured strain agreed with the expected value calculated from the length change of the tape. For strains up to 7.5%, applied strain is uniformly distributed to the stack, except for the top right corner. At 10% strain, we observed a slightly inhomogeneous strain distribution, especially near the clamps at the sides. This inhomogeneity is probably due to plastic deformation of the support layer of the UV tape. However, as graphene was placed at the center of the tape in our experiments (typically the tape is 3 cm in length, while the graphene has a 1×1 cm^2 size), the influence of this strain inhomogeneity in the graphene was limited. Although the strain distribution can be optimized by modifying the support layer of the UV tape, the size of the tape, or the clamping geometry, such optimization is beyond the scope of this work. Importantly, to our knowledge this work already enables the fabrication of the highest-density array of the longest GNSs to date, as shown in the benchmark (Fig. 1k).

2. Analysis of crack formation

As claimed in the original manuscript, the applied strain in graphene induces the formation of linear cracks. However, we did not present detailed analysis of the crack formation. By following the reviewer's suggestion, we have measured the crack density while changing the applied tensile strain to the graphene/tape stack. As shown below (new Supplementary Fig. 9a), we observed an increase of the crack density with the tensile strain. At the low strain (2.5%) the crack density was rather low, but increasing the strain to 5% induced clear cracks with higher crack density. The crack became longer and more obvious by further increasing the strain, resulting in the formation of a number of aligned graphene ribbons. The density plotted against the applied strain (Fig. 2c and Supplementary Fig. 9b) almost saturates at 10%. Therefore, we conclude that >10% strain is better than 5% strain in

terms of the GNS density, thus we have increased the strain from 5% to 10-15% in the revised manuscript to make more uniform and denser GNS arrays.

Concerning the chiral angle, it is determined by the stretch direction, because the residual strain acts as one of the main deriving forces to make nanoscroll structure. Although the value of the tensile strain was not completely uniform, the DIC experiments showed that the strain direction was largely preserved, resulting in cracks always aligned perpendicularly to the applied strain. Hence, the chirality of the ribbons is not affected by the inhomogeneities of the strain. Scroll angle (*i.e.*, chiral angles) of GNSs have been newly measured by TEM and STEM, as shown in new Fig. 1g-i of the main manuscript and Supplementary Fig. 4, respectively. The fast Fourier transform (FFT) patterns in Fig. 1g-i confirm that the chiral angle can be determined by the direction of the extension.

New supplementary figure (Supplementary Fig. 9):

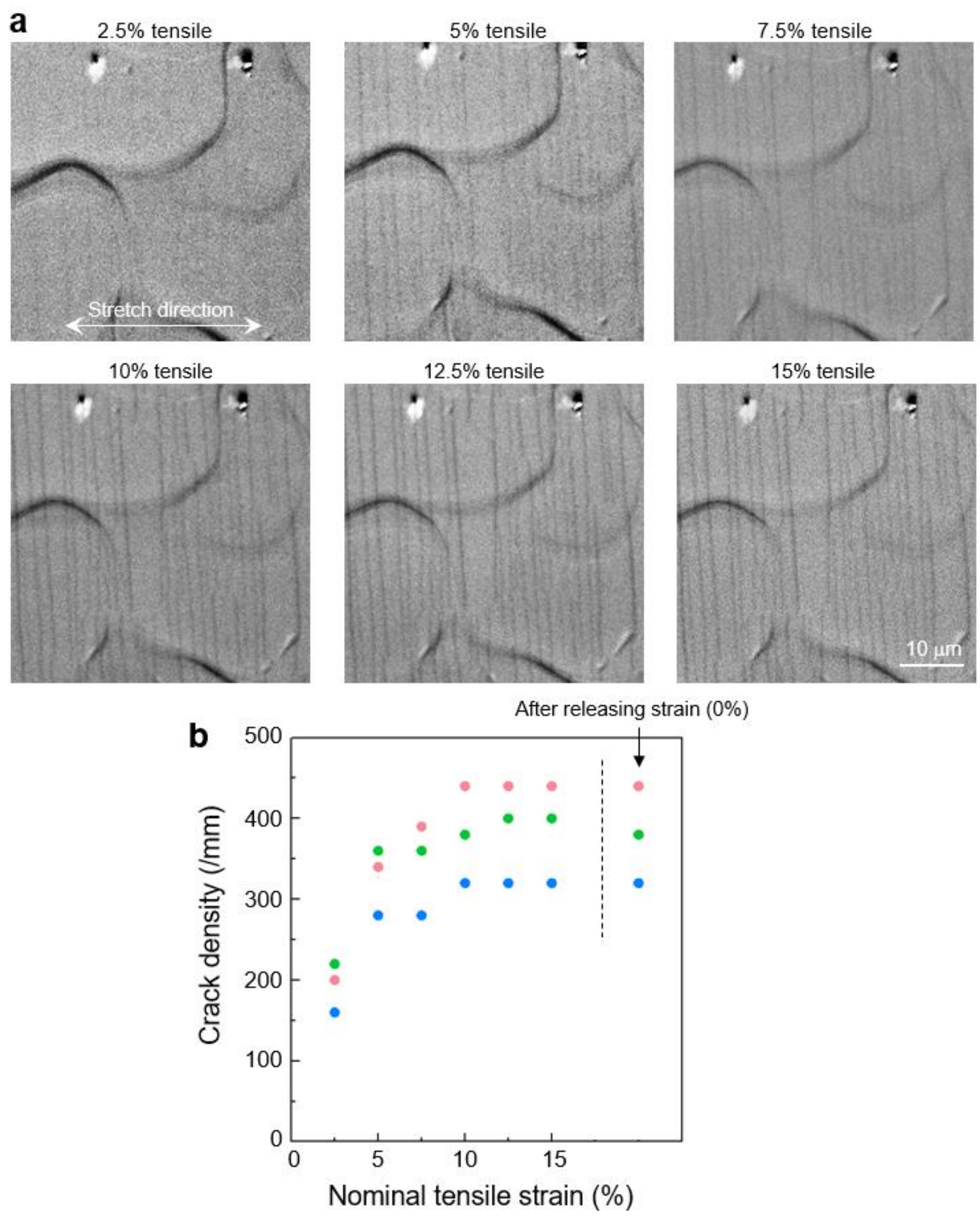

Supplementary Fig. 9. Crack formation while applying strain to the UV tape. a, Confocal laser microscope images of monolayer graphene on the UV tape captured under different applied strains

(the strain values are the nominal values calculated from the change of the tape length). To monitor the crack formation, we captured images at identical positions. It is clearly seen that cracks are formed by extending the UV tape, with the number of cracks increasing with strain. **b**, Crack density plotted against the applied tensile strain. The plot shows three different positions of the stack, indicating a similar trend regardless of the area. The crack density practically saturates at ~10% strain.

New Fig. 1c,g-i:

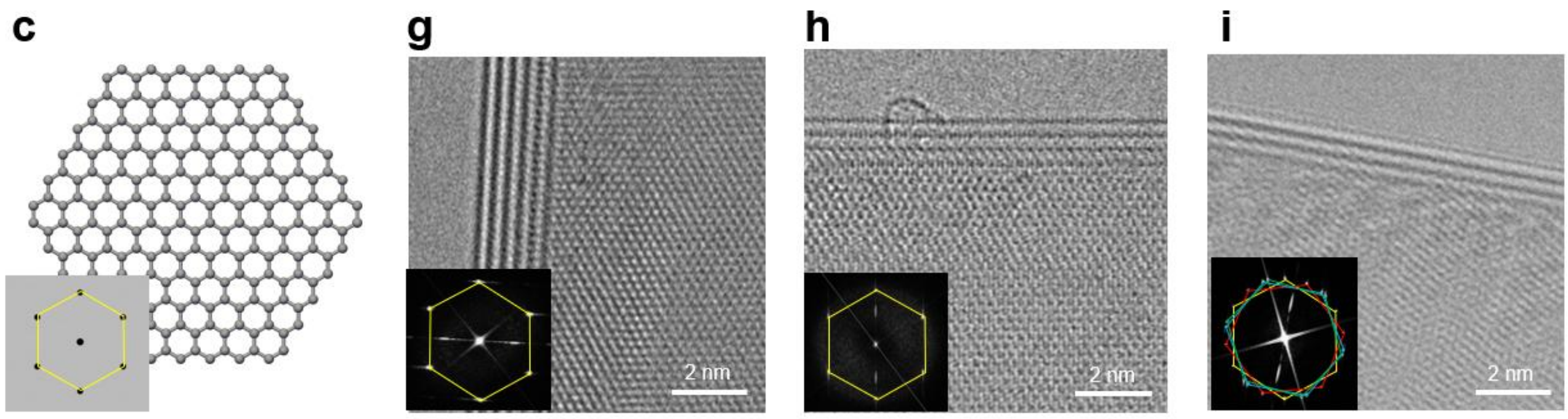

Fig. 1. c, Schematic atomic structure and corresponding simulated diffraction pattern (inset) of the hexagonal grain shown in **b. d-f**, GNSs made by stretching the UV tape/graphene stack along the directions indicated by the arrows. (**d**) zigzag GNSs, (**e**) armchair GNSs, and (**f**) chiral GNSs. **g-i,** Top-view high-resolution TEM images of the corresponding GNSs. Insets are the FFT patterns.

Pages 5-6 of the revised manuscript:

"To confirm the chirality-selective formation of nanoscrolls, the GNS samples were further characterized using high-resolution electron microscopes. Fig. 1g-i display the top-view transmission electron microscopy (TEM) images of the nanoscrolls, together with their corresponding FFT patterns shown in the insets (the corresponding high-resolution scanning transmission electron microscopy (STEM) images are displayed in Supplementary Fig 4). In Fig. 1g-i, we clearly observed the layered structure reflecting the rolling structure. The FFT patterns shown in the insets confirmed the formation of zigzag and armchair nanoscrolls, which are solely determined by the stretch directions. It is worth noting that it is difficult to grow chirality-controlled carbon nanotubes, while our unique tensile-based method allows to make zigzag and armchair scrolls in a highly controlled manner. In addition, due to the scrolling structure, all the rolls have the same chirality, which is completely different from a MWCNT in which each layer has different chiralities.[19] Chiral GNS exhibited interesting FFT patterns (inset of Fig. 1i); the number of the set of spots almost corresponding to the number of rolls. Although the origin of the multiple patterns is not clear, it may reflect a cone-like

chiral nanoscroll structure. The possibility of the chirality control, including zigzag and armchair, is an advantage of our strain-assisted nanoscroll formation, which offers new opportunities to explore chiral-related phenomena."

3. Actual strain in graphene on the UV tape (Raman analysis)

The reviewer asked about the actual strain in graphene induced during stretching the graphene/tape stack. To respond this question, we have performed *in-situ* Raman measurements of the graphene, while applying the tensile strain. New Supplementary Figs. 9 and 10 display Raman spectra under different strains. Both G and 2D bands shift to lower wavenumbers, proving that the tensile strain is indeed applied to graphene supported on the UV tape.

As it is well acknowledged that 2D band is more sensitive to the strain than the G band (see *e.g.* T. M. G. Mohiuddin, *Phys. Rev. B*, **79** 205433 (2009)), our Raman 2D band showed clearer difference. Importantly, we confirmed that some residual strain exists in graphene even after relaxing the graphene/tape stack (red spectrum in Supplementary Fig. 11a,b). One reason for this is the plastic deformation of the underlying UV tape, which remains slightly elongated after releasing the strain (we speculate the tape suffers a residual ~2% strain from the DIC analysis). This remaining strain in the graphene ribbons and solvent intercalation result in the GNS arrays via detaching from the tape surface

We also performed a more detailed analysis for small tensile strains. As shown in the new Supplementary Fig. 12, both G and 2D bands exhibited large downshift at 1-1.5% strain, indicating that the graphene suffered intensive strain up to 1.5%. However, when increased to 2%, the some of the effective strain was released, which we consider is due to the formation of cracks perpendicular to the extension direction.

New supplementary figure (Supplementary Fig. 11):

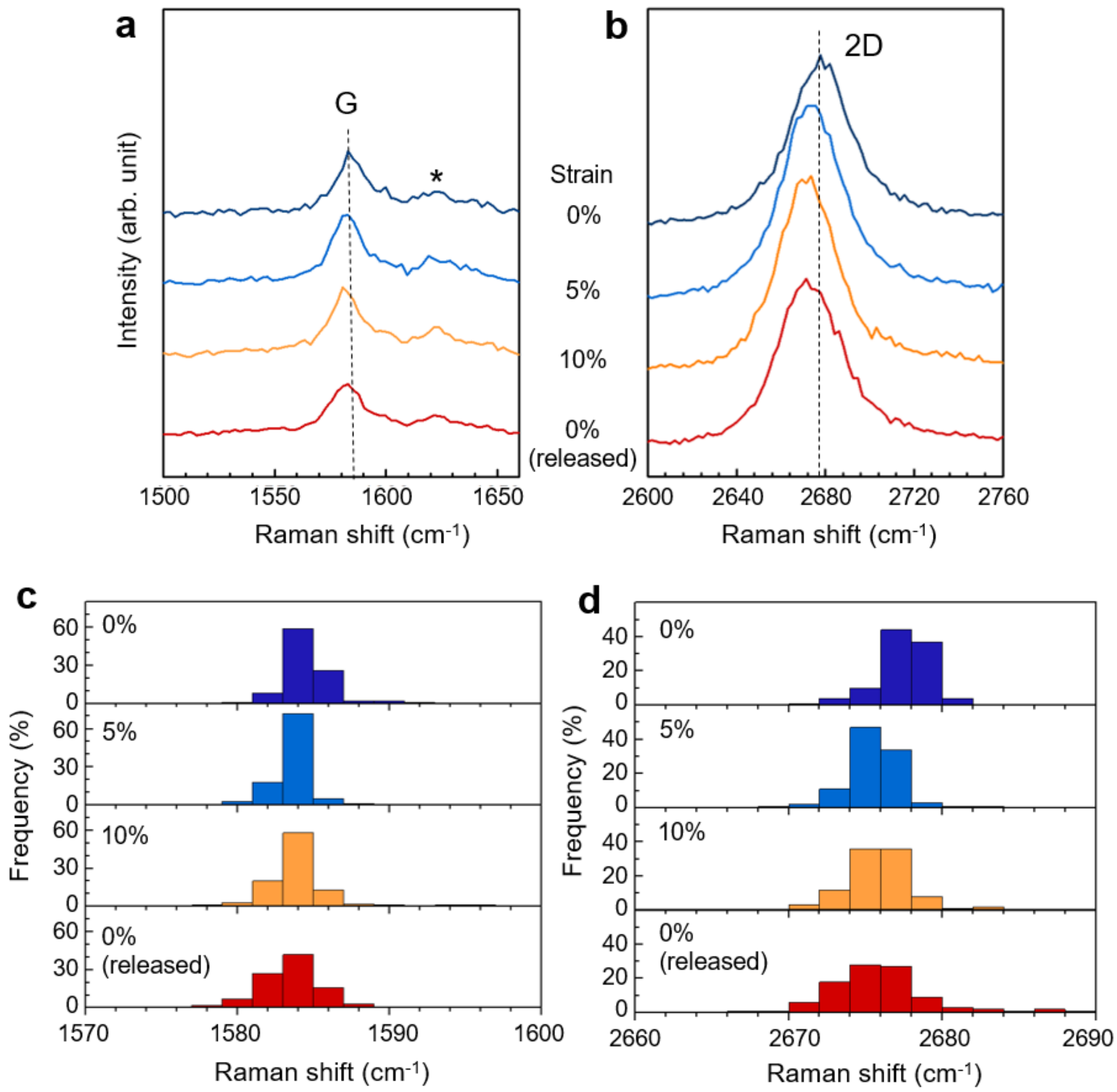

Supplementary Fig. 11. Raman spectra of graphene on the strained UV tape. a,b, Raman spectra of monolayer graphene measured while applying tensile strain to the graphene/tape stack: G band (**a**) and 2D band (**b**). The strain values are nominal values calculated from changes of the tape length. The bottom spectra (red) were taken after releasing the strain of the tape. Asterisk in **a** represents a peak from the adhesive layer of the tape. **c,d,** Distributions of peak positions of G band (**c**) and 2D band (**d**).

New supplementary figure (Supplementary Fig. 12):

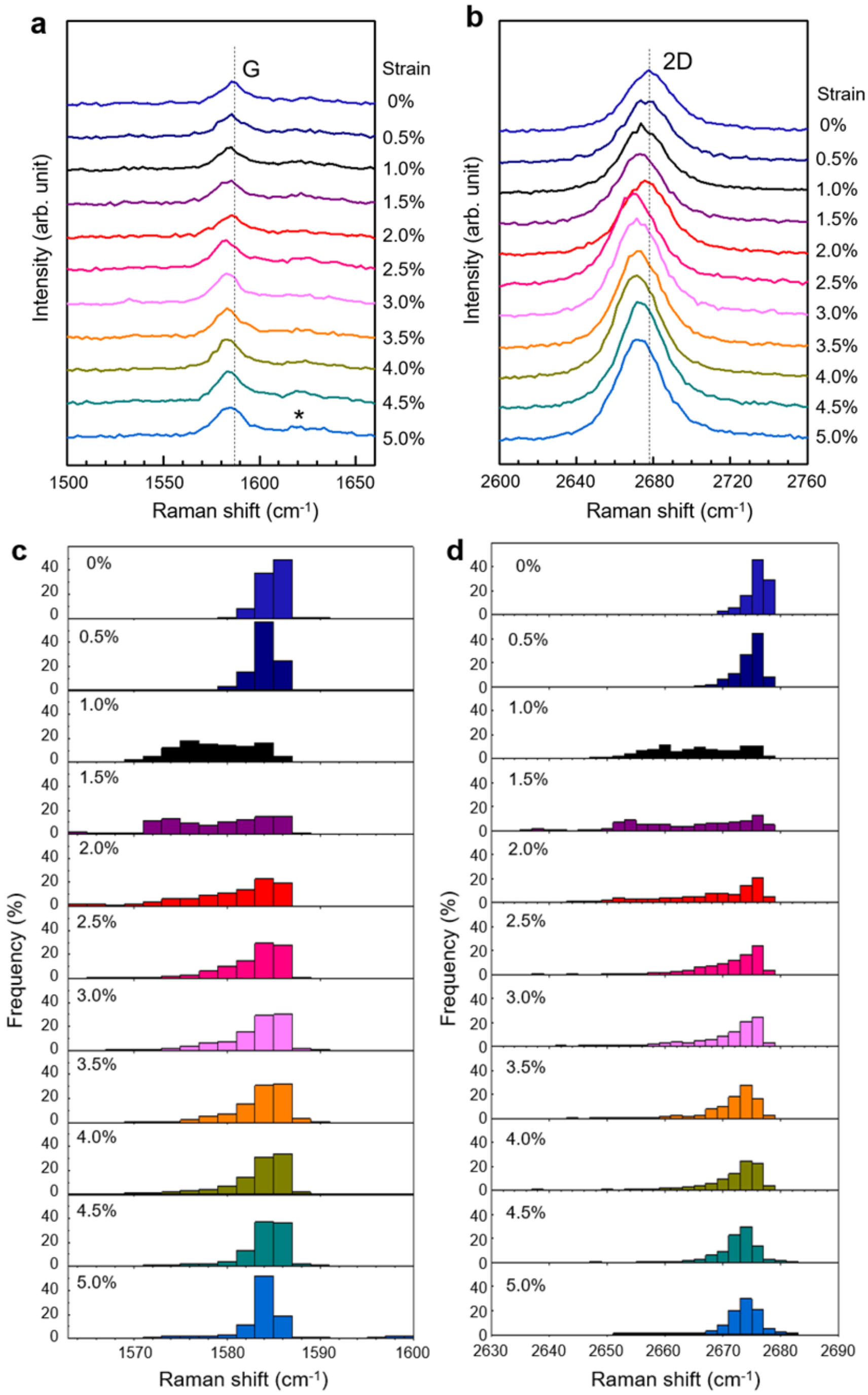

Supplementary Fig. 12. Raman spectra of graphene on the UV tape under low strain loads. a,b, Raman spectra of monolayer graphene measured while applying tensile strain (up to 5%) to the graphene/tape stack: G band (**a**) and 2D band (**b**). The strain values are the nominal values applied to the UV tape. Asterisk in (**a**) represents a peak from the tape. **c,d,** Distributions of the positions of G band (**c**) and 2D band (**d**).

Significant downshifts were observed of both G and 2D bands in the range of 1.0~1.5% strain (Supplementary Fig. 12c,d), indicating the introduction of substantial tensile strain in the graphene. However, when the graphene/UV tape stack was extended to 2.0%, the fraction of spectra exhibiting downshifted G and 2D bands decreased. As the applied strain was increased to 5%, both the 2D and G bands gradually shifted back to higher wavenumbers, as evident in the decrease of the low-wavenumber tails of the histograms. This behavior suggests the onset of crack formation at approximately 2.0% strain, followed by the progressive formation of additional cracks with increasing applied strain, which gradually relieves the tensile strain stored in the graphene (but the overall peak distributions are slightly shifted to lower wavenumbers as compared with those before applying strain). In addition, an increase of the 2D band intensity was also observed with increasing the strain, suggesting the slight decoupling from the tape surface.

Pages 7-9 of the revised manuscript:

"To fully understand the mechanism of the nanoscroll formation, we first studied the actual tensile strain that is applied to the UV tape during the mechanical extension of the graphene/tape stack. Fig. 2a, Supplementary Fig. 8, and video 1 show the strain distribution in the UV tape determined by a digital image correlation (DIC) method (the details of the measurement are described in Supplementary Fig. 8). As seen in Fig. 2a, the DIC result confirmed that the measured tensile strain is consistent with the strain calculated from the extended length of the UV tape. However, when the tensile was increased to ~10%, some inhomogeneity was observed due to the deformation of the UV tape.

Next, we determined the crack formation in the graphene sheet using a laser confocal microscope while applying strain. As shown in Fig. 2b, Supplementary Figs. 9 and 10, extending the stack induces cracks that are oriented normal to the applied strain. The crack density increased with the strain up to ~10% and then almost saturates up to 15% (Fig. 2c and Supplementary Fig. 9). The highest crack density reached 440 mm^{-1}, corresponding to an average ribbon width of 2.3 μm, with each of these graphene ribbons producing a single nanoscroll after detaching from the tape surface.

It is also important to measure the actual strain transferred to the graphene sheet from the UV tape, because the crack formation in graphene and possible slippage can reduce the actual strain. Therefore, we measured *in-situ* Raman spectra of the graphene while extending the graphene/tape stack. Fig. 2d shows the evolution of the Raman G and 2D bands with respect to the nominal strain (more detailed data can be found in Supplementary Figs. 11 and 12). The strained graphene exhibited downshift of the 2D band when the stack was extended. Part of this shift is retained even after releasing the strain of the stack. This result indicates that the graphene experiences tensile strain even

after relaxing the UV tape. This residual strain is supposed to trigger the nanoscroll formation.

To investigate the role of the additional stimuli employed to produce the GNSs, we measured the adhesion force of the UV tape after different treatment including UV exposure, solvent treatment, and heating (Supplementary Fig. 13). The pristine UV tape showed a high adhesion force (0.12 N/mm), which decreased to 0.015 N/mm after exposure to UV light. Post-UV heating and solvent treatment further decreased the adhesion force to 0.009 N/mm, with toluene being the most effective among the studied solvents (Supplementary Fig. 13c).

Therefore, after applying and releasing the tensile strain, the graphene/tape stack was treated with toluene while being heated on a hot plate. The reduced adhesion eases the local detachment of graphene from the tape surface, enabling the graphene to roll up into nanoscrolls. We think that the contact with solvent not only weakens the adhesion force of the UV tape, but also promotes the scrolling through intercalation at the strained graphene and tape interface, a mechanism that has been widely used to make graphene scrolls.[14,18,28] Because the crack orientation in graphene is predetermined by the applied tensile strain, the scrolling preferentially initiates from these predefined edges. Consequently, the scrolling direction and chirality are deterministically controlled, allowing to realize large arrays of aligned GNSs."

New supplementary figure (Supplementary Fig. 13):

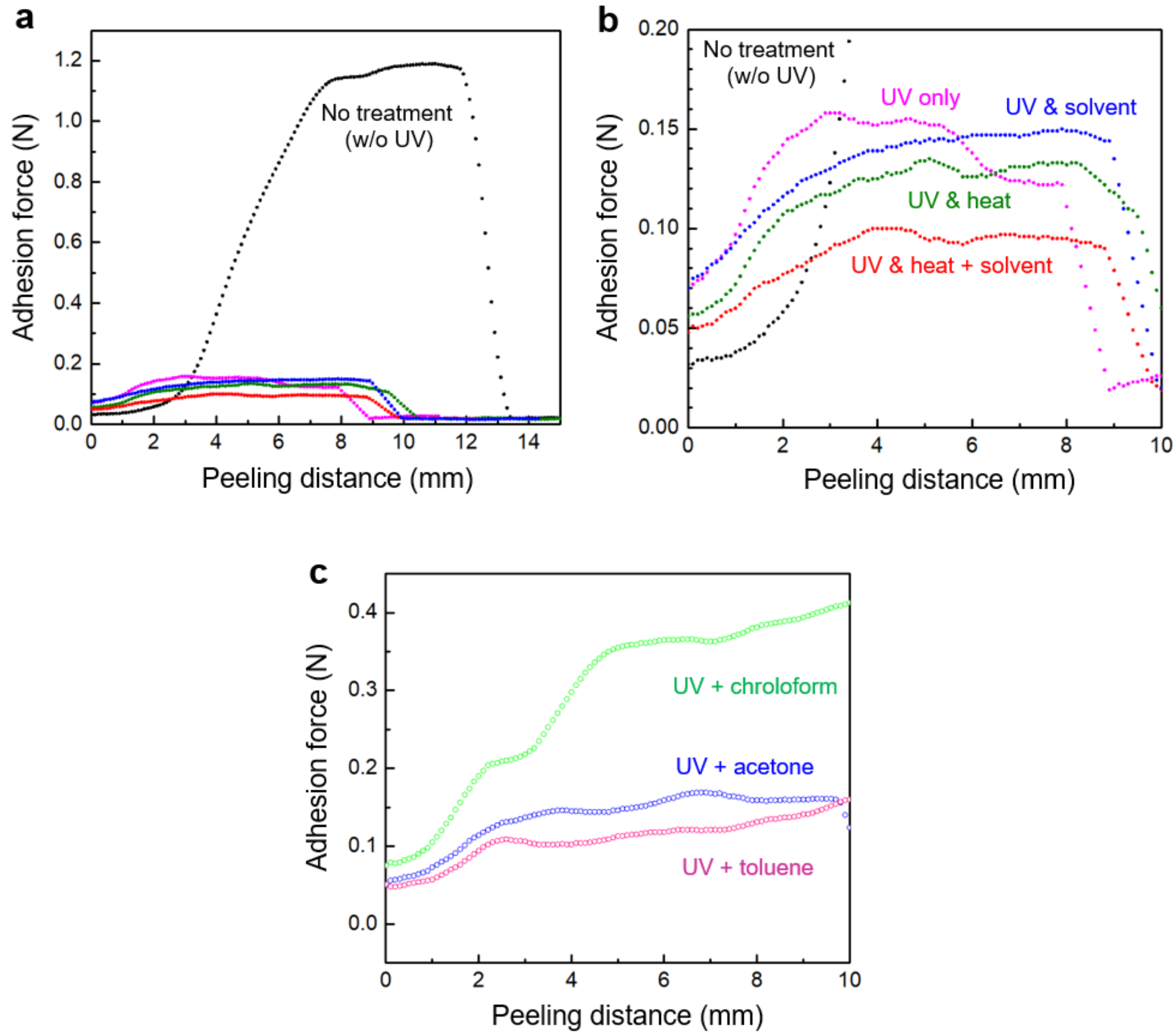

Supplementary Fig. 13. Influence of the post-UV treatment on the adhesion force of the UV tape
a, Comparison of adhesion force of UV tapes measured after different treatments. The adhesion force was measured by removing the treated UV tape from a bare SiO_2/Si substrate (10 mm width). No UV illumination (black) maintained strong adhesion force. **b,** After UV illumination the adhesion significantly decreased (pink). The drop of solvent (acetone) slightly decreased the adhesion force (blue), but the heat treatment (80 ºC) was more effective to decrease the adhesion force (green). Interestingly, combining the heat treatment and solvent showed the lowest adhesion force (red). **c**, Solvent dependence of the adhesion force (without heat treatment). To synthesize GNSs from the strained graphene, we used toluene and acetone, which are more effective than chloroform.

2. The provided SEM and optical images (e.g., Fig. 1c, 1g, and Supplementary Fig. 5) reveal that a significant number of the nanoscrolls exhibit noticeable morphological defects. Specifically, many scrolls appear twisted or wavy rather than perfectly rectilinear, and the winding tightness seems to

vary significantly, which are critical structural flaws that may affect their intrinsic properties.

The authors should provide a more critical discussion on the origins of these morphological defects. Additionally, for the fabrication of graphene–MoS_2 heteronanoscrolls, the reproducibility or yield, nor the degree of structural uniformity (e.g., layer sequence consistency or scroll completeness) are discussed. Clarifying how reliably such heteronanoscrolls can be produced over larger areas would be important for assessing their practical relevance and scalability.

To improve the quality of our GNS arrays, we have conducted a detailed investigation of the synthesis method. The images with the improved results are shown in Fig. R2-3. Specifically, we found that increasing the applied strain and tuning solvent are effective to improve the quality of aligned GNSs. We found that increasing strain from 5% to 10-15% increased the crack density, allowing to make longer graphene ribbons with a higher density. In addition, changing solvent from acetone to toluene was found to contribute to the formation of well-wrapped GNSs. As toluene remains on the tape surface for longer time than acetone due to its lower vapor pressure, toluene can assist GNS formation more effectively than acetone. Through such optimization, we have obtained a high-density of well rolled GNSs, as compared in Fig. R2-3.

Through these optimizations, in this revision we have significantly improved the quality, density and uniformity of the GNS arrays, while maintaining deterministic control of the scrolling direction and chirality. These results demonstrate the reproducibility and scalability of our fabrication method and address the reviewer's concerns regarding the structural quality of the resulting GNSs.

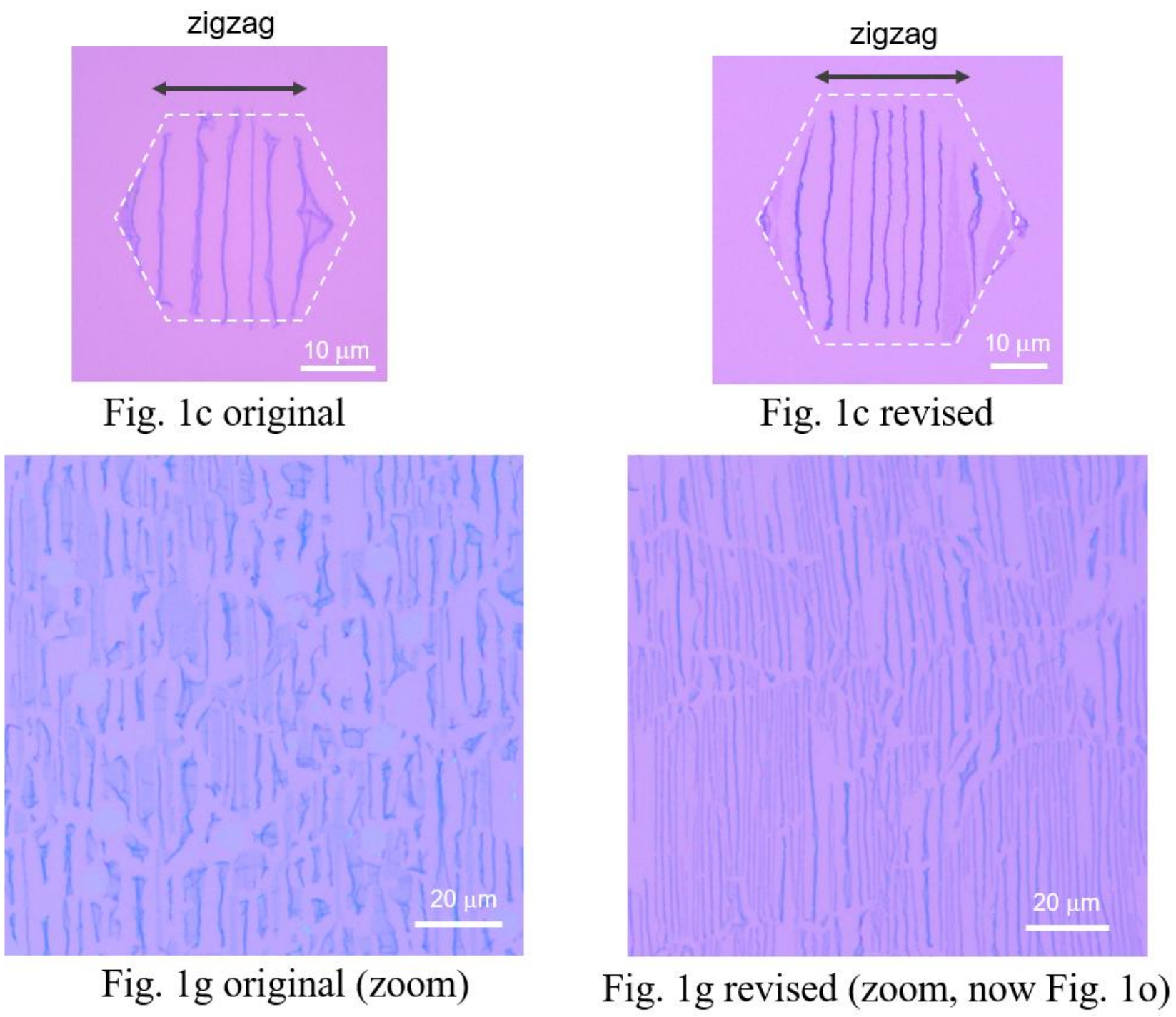

Fig. R2-3. Comparison of the original and revised images.

The uniform synthesis of MoS_2-graphene heteronanoscrolls (co-scrolls) is more challenging than the synthesis of GNSs, as monolayer graphene needs to scroll together with MoS_2, which has three-atom thickness. Nonetheless, after the optimization of tensile strain and solvent, we could obtain arrays of heteronanoscrolls as those shown in Fig. 5b and Supplementary Fig. 17. To address the reviewer's concern, we have added the optical image of the co-scroll heterostructure in Fig. 5b of the revised manuscript. We have also added the low magnification optical image and the image of GNSs that partially contain MoS_2 (made with low density MoS_2 grains) in new Supplementary Fig. 17. From Supplementary Fig. 17, it is evident that we can make uniform array of MoS_2-graphene co-scrolls in high yield.

Page 14 of the revised manuscript:

"Fig. 5b displays the optical image of a dense array of MoS_2-graphene co-scrolls. As shown in Supplementary Fig. 17, bright contrast originates in MoS_2. Therefore, Fig. 5b indicates a high yield of the aligned co-nanoscrolls."

New figure (Fig. 5b):

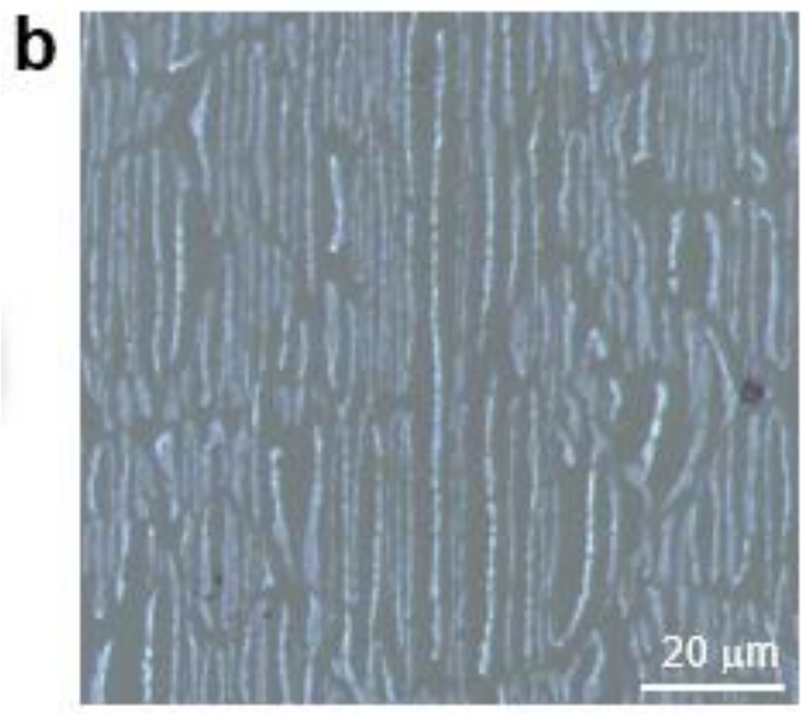

Fig. 5b, Confocal laser microscope image of the aligned co-nanoscrolls.

New supplementary figure (Supplementary Fig. 17):

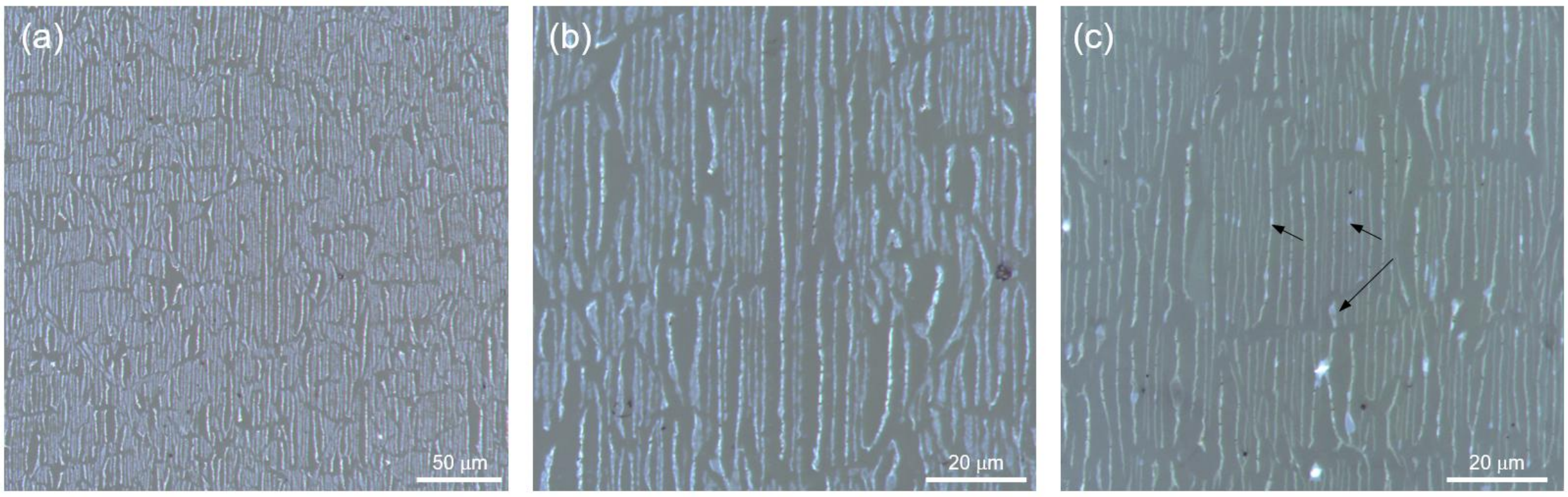

Supplementary Fig. 17. Optical images of MoS_2-graphene co-nanoscrolls. a,b, Most of nanoscrolls are heteronanoscrolls consisting of MoS_2 and graphene. **c,** Graphene nanoscrolls partially formed heteronanoscrolls with MoS_2. Bright contrast indicated by the arrows indicate the presence of MoS_2.

3. The correlation between Raman signals and specific structural parameters (number of rolls, interlayer spacing, residual strain, etc.) is not quantitatively established. The authors attribute the low I_{2D}/I_G ratio, broadened 2D band, and G-band red shift to multi-layer coupling and residual strain, but no control experiments with different strain magnitudes (e.g., 3%, 5%, 7% extension degree) or GNS layer numbers are performed to verify these attributions.

As mentioned by the reviewer, discussion on the relationship between applied strain and Raman spectra of resulting GNSs was lacking in the original manuscript. The Raman spectrum of a GNS contains contributions from multiple graphene layers within the scroll, including inner turns with small diameters and outer turns with larger diameters.

Therefore, establishing a direct correlation between specific structural parameters, such as number of rolls, and the Raman spectra is a complex topic which is beyond the scope of the present manuscript. Our measurements of GNSs fabricated under different conditions did not reveal a clear dependence of the Raman features on the GNS structure. Regarding the interlayer spacing, the observed interlayer distance of GNSs (0.34 nm) is comparable to that of graphite, as confirmed by the cross-sectional TEM images (Fig. 1g-i, Fig. 3m,n, and Supplementary Fig. 16). This suggests that once a graphene sheet starts scrolling, van der Waals interaction stabilize the formation of a tightly bound nanoscroll, leading to the characteristic broad 2D band shown in Fig. 3e,g. Therefore, we consider the Raman spectrum is primarily determined by the final scroll structure rather than by the amount of applied strain to the tape.

In the original manuscript, we attributed the difference of the G band positions of GNSs and monolayer graphene to the residual strain existing in GNSs. However, we now think that this explanation is not sufficient. Monolayer graphene transferred on a SiO_2 substrate is known to suffer partial p-type doping from SiO_2 surface having functional groups, such as -OH groups, resulting in the G band at relatively high wavenumbers, 1587-1591 cm^{-1}. On the other hand, a GNS is weakly affected by a SiO_2 substrate, because the ratio of carbon atoms close the substrate surface is very limited. Instead, the curvature of a GNS can influence the G band position (this is a kind of strain due to curved sp^2 network), like multiwalled carbon nanotubes which show the G band at 1583-1585 cm^{-1} (for example, A. M. Rao *et al.*, *Phys. Rev. Lett.* **84**, 1820 (2009)). We have modified the original

paragraph explaining the difference of the G band positions of GNSs and graphene. We appreciate the reviewer for suggesting us the careful analysis of the Raman spectra.

Page 10 of the revised manuscript:

"Monolayer graphene transferred on a SiO_2 substrate generally experiences p-type doping from SiO_2 surface having functional groups,[33,34] such as silanol groups, resulting in the upshift of the G band to relatively high wavenumbers (1587-1591 cm^{-1}).[35] On the other hand, a GNS is weakly affected by SiO_2 surface, because the ratio of carbon atoms close to the SiO_2 surface is very limited. Therefore, a combination of residual strain, curvature, and limited contact with the SiO_2 surface can be attributed to the observed G band red shift of the transferred GNRs."

New references:

(33) Lee, J. et al. Charge inhomogeneity of graphene on SiO_2: dispersion-corrected density functional theory study on the effect of reactive surface sites. *RSC Adv.* **4**, 37236 (2014).

(34) Goniszewski, S. et al. Correlation of p-doping in CVD graphene with substrate surface charges. *Sci. Rep.* **6**, 22858 (2016).

(35) Ago, H. et al. Controlled van der Waals epitaxy of monolayer MoS_2 triangular domains on graphene. *ACS Appl. Mater. Interfaces* **7**, 5265 (2015).

4. Molecular dynamics (MD) simulations are conducted at 0 K, which deviates significantly from the experimental conditions involving 90 °C heating and organic solvent treatment. Though the authors claim that thermal effects are not essential for the scrolling phenomena examined in the study, it still remains possible for them to remarkably affect graphene's flexibility, interlayer van der Waals interactions, and scrolling dynamics. Additionally, the MM calculations only analyze the energy balance between bending and van der Waals interactions, but do not involve the effect of solvent molecules on interlayer interactions.

We thank the reviewer for this useful advice. By responding to this reviewer's comment, we have performed additional MD simulations at the elevated temperature (90 °C (363 K)). The new data calculated at 363 K has been added as new Supplementary video 6. We found that the high temperature induces thermal fluctuation of the graphene ribbon and makes the scrolling faster, as compared with the result at 0 K. The dynamic behavior at 90 °C looks more realistic. As for the scrolling behavior, we did not see fundamental difference, showing the importance of the initial scroll angle.

As can be seen in the TEM images (Fig. 1g-i, Fig. 2m,n, and Supplementary Fig. 16), most of the nanoscrolls show the intra-layer distance of 0.34 nm, which indicates the absence of solvent molecules between neighboring rolls. We think that solvent intercalation assists the initial formation of nanoscrolls but does not affect intra-layer interaction. The initial stage of the scrolling is affected by many uncertain parameters, such as strain release, solvent evaporation and diffusion, and graphene-adhesive layer interaction. Therefore, in this manuscript we did not include solvent molecules in our MD simulation to discuss the fundamental phenomenon.

Page 14 of the revised manuscript:

"The nanoscroll formation was also simulated at the temperature used in our actual experiments (363 K). The results are compared in Supplementary video 6 with those at 0 K. We found that the high temperature of 363 K induces thermal fluctuation of graphene ribbons and speeds up the scrolling as compared with that calculated at 0 K. However, there was no essential difference in the nanoscroll formation."

Page 22 of the revised manuscript (Methods section):

"MD simulations were performed for different environment temperatures, 0 K and 363 K."

New Supplementary video 6: the following is a snapshot from this video

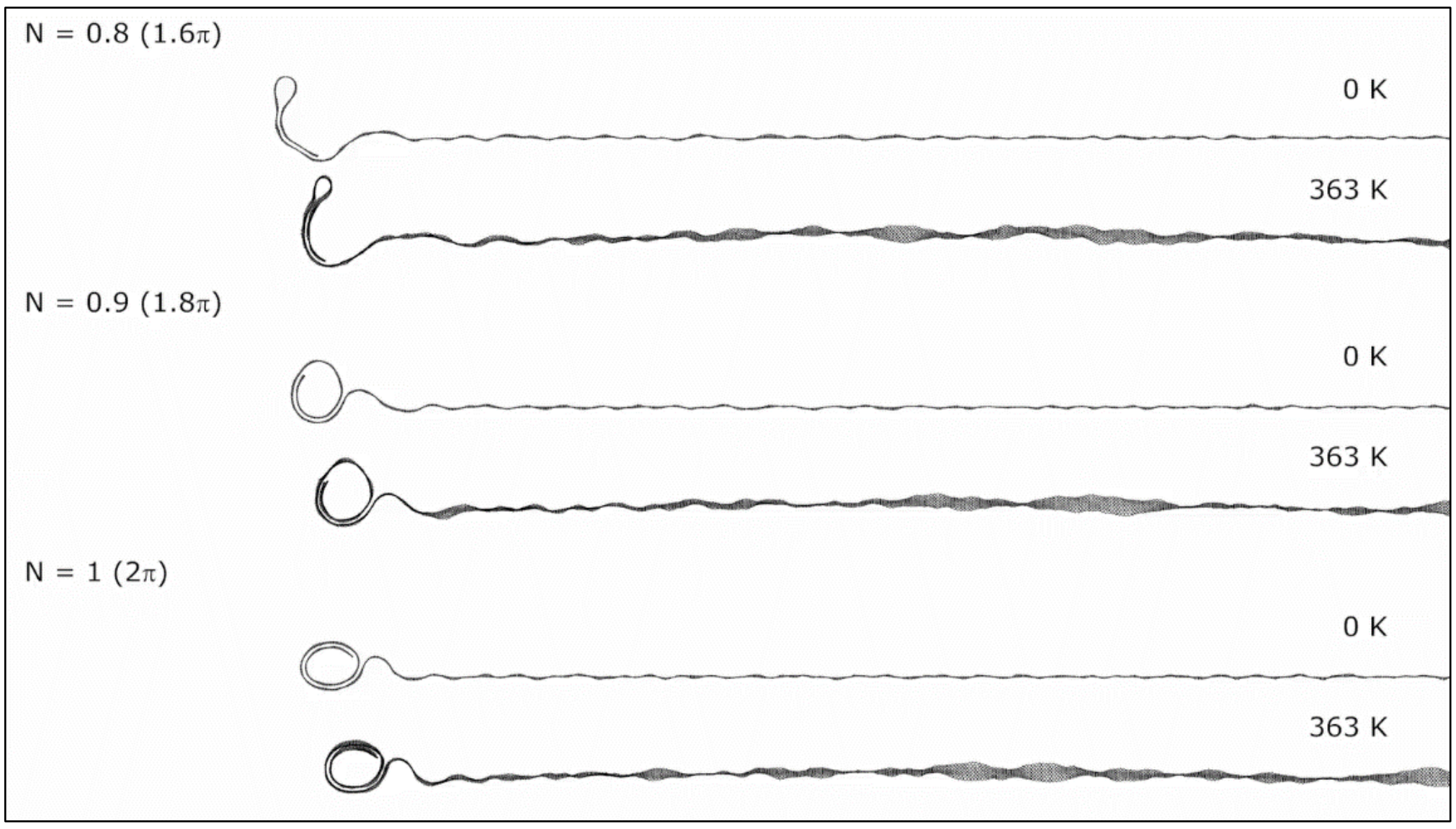

5. For the atomic simulation part, the size, boundary conditions of the simulation system and the basis for force field selection need to be clarified. The paper mentions that "initial edge curvature" is a triggering factor, but fails to explain how to regulate the edge curvature of graphene. Could you

supplement the regulation method of edge curvature and corresponding experimental verification to further strengthen the rationality of the mechanism?

We acknowledge the reviewer for this insightful comment. For the details of the MD simulation, we have modified the experimental section. Briefly, we used an Adaptive Intermolecular Reactive Empirical Bond Order (AIREBO) potential, which is widely used for calculations with carbon materials, such as graphene and carbon nanotubes. The unit size has length L = 204.6 nm and width W = 1.99 nm, containing 16,629 carbon atoms. In our MD calculations, we did not use periodic structure.

The GNS formation is a nanoscale, dynamics phenomenon, thus it is not easy to correlate with the detailed experimental parameters. From the experimental view point, increasing the strain (10-15%) and using low vapor pressure toluene instead of middle strain (5%) and high vapor pressure acetone lead to a higher density GNSs with straighter and more densely packed structures. Therefore, large strain, narrow graphene ribbon width (*i.e.*, high crack density), and solvent intercalation are important process parameters. The residual strain that remained after releasing the applied strain in the graphene/UV tape stack can produce faster initial speeds which can potentially increase the probability of scroll formation, while the speed of solvent intercalation at the graphene/tape interface can related with the assist the scrolling. However, as mentioned above, it is difficult to directly correlate MD simulation with the experimental result.

Page 22 of the revised manuscript (Methods section):

"As illustrated in Supplementary Fig. 28, the initial GNS configuration was constructed by rolling one end of a finite graphene ribbon with zigzag edges (length L = 204.6 nm; width W = 1.99 nm, containing 16,629 carbon atoms, without a periodic condition) into an Archimedean spiral on a substrate (0.34 nm below the graphene sheet), defined by the following polar equation:"

Pages 23 of the revised manuscript (Methods section):

"Interatomic forces were modeled using an adaptive intermolecular reactive empirical bond order (AIREBO) potential[57] for C–C interactions and a continuum Lennard–Jones (12–6) wall potential[58] for interactions between the graphene and the substrate. This AIREBO potential is widely used for calculations of carbon materials including graphene."

Reviewer #3 (Remarks to the Author):

This manuscript presents a highly efficient approach for the large-scale synthesis of graphene nanoscrolls (GNSs) with controlled orientation, utilizing a UV tape. The authors demonstrate that applying tensile strain via tape stretching, followed by the application of external stimuli, successfully yields dense arrays of aligned GNSs. While the findings reported here present a new approach, key parameters in the manuscript are lacking. The authors measure a variety of properties of the GNS, but many experimental details and some details of the measurements and their description need to be added.

While the results are promising, several key issues must be addressed to fully substantiate the claims and ensure the robustness of the methodology.

We acknowledge the reviewer for reviewing our manuscript and giving us many important comments. We also thank the reviewer for recognizing the importance of our research on GNSs. Based on the reviewer's comments, we have substantially revised our manuscript by performing many additional experiments. We have also added detailed experimental conditions with the main focus on the relationship between the synthesis condition and the resulting GNS structure.

Question 1: In the abstract, the authors mention that the work results … in the highest GNS density and length. Similar, even higher lengths have been reported for example in Li et al., Commun Phys 1, 44 (2018). https://doi.org/10.1038/s42005-018-0043-2.

Thank you for pointing out the result of this *Commun. Phys.* paper. Actually, we were aware of this paper and cited as ref. 31 in the original manuscript. In this *Commun. Phys.* paper, the authors synthesized GNSs by dispersing CVD graphene in liquid solvent to make nanoscrolls. As shown below (Fig. R3-1(left)), the liquid-based process resulted in the random network of bundles of multiple GNSs without any control of orientations. Thus, we think that the length reported in the *Commun. Phys.* paper. correspond to the length of GNS bundles, because it is difficult to distinguish individual nanoscrolls. In contrast, our method produces well-defined isolated GNS structures (please refer Fig. 3m,n, and Supplementary Fig. 16 of the revised manuscript for cross sectional TEM images), which allows precise determination of GNS lengths. Therefore, we believe that our claim that the highest density and length is legitimate. However, to avoid misunderstanding, we have modified the main text to clarify that we measured isolated GNSs.

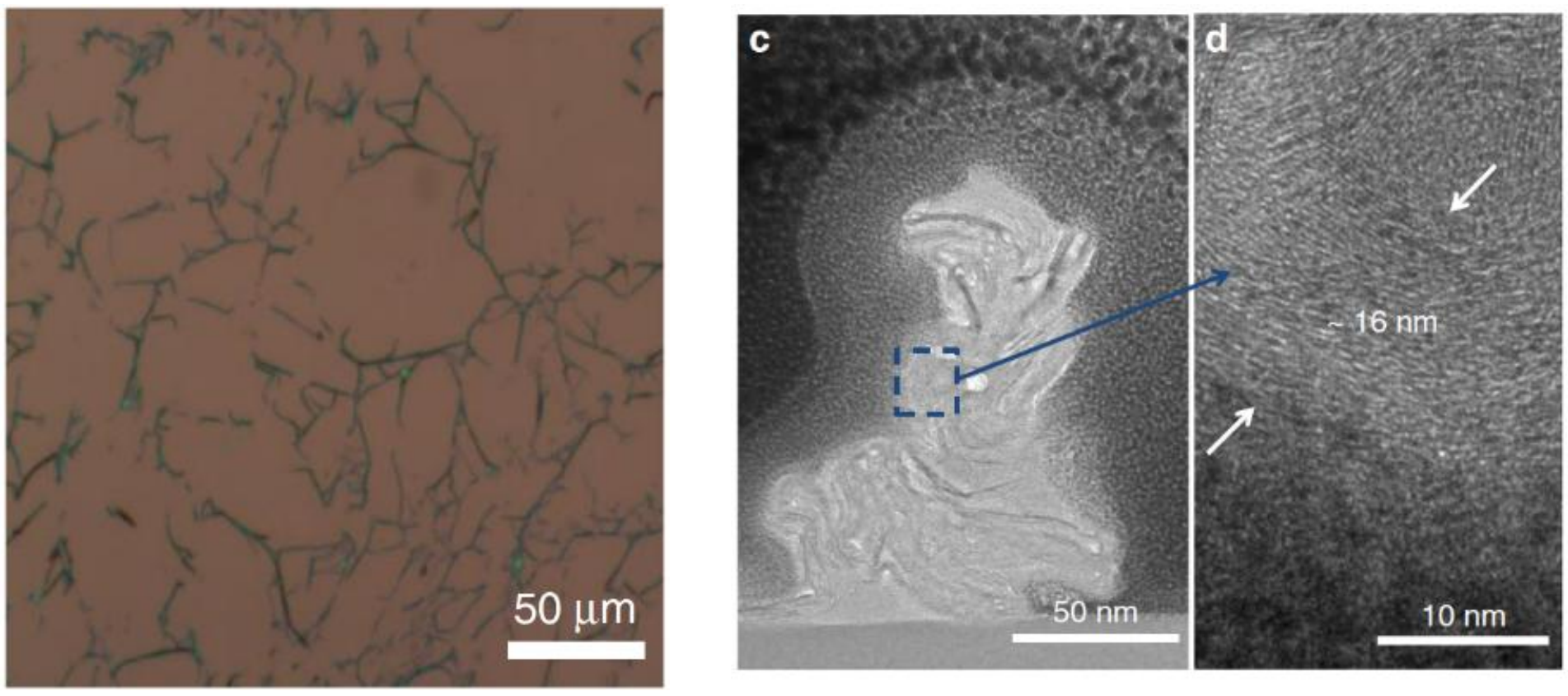

Fig. R3-1. Optical micrograph (left) and cross-section TEM images (right) of liquid-based GNSs, reproduced from *Commun. Phys.* 1, 44 (2018).

Figure 1 caption:

"**Fig. 1h**, Comparison of the density and height of isolated GNSs with the literature."

Page 2 (abstract) of the revised manuscript:

"This strain-assisted method is highly efficient, yielding isolated GNS with the highest reported density (~380 nanoscrolls/mm) and maximum length (90 μm) achieved to date."

Question 2: The manuscript lacks detailed descriptions of the experimental detail. The duration of UV illumination has a significant impact on the adhesion of the functional tape and graphene, which will further influence the strain transfer efficiency and subsequent peeling/curling behavior. Providing these details such as the fabrication process of the UV tape and the illumination time is essential for the reproducibility of the method.

Additionally, more discussions and characterizations about the influence of tape-graphene adhesion on the formation of GNSs should also be provided. Furthermore, after releasing the strain, an excess stimulus is required. The authors mentioned the use of an organic solvent, such as acetone. Does this solvent have any impact on the tape? For example, causing the tape to swell? It should be explained in detail.

The main factor which influences the adhesion force is the total energy applied to the UV tape. Although the required energy of the UV to decrease the adhesion force (1000 mJ/cm^2) was included in our original manuscript, as pointed by the reviewer, this information was not enough. We have added the exposure time (11 sec) in addition to the UV power (~93 mW/cm^2) in the revised manuscript. Since the composition and the fabrication process has been already reported in ref. 29

(*Nat. Electron.* **7**, 119 (2024)), we cited this paper in Methods section.

As the reviewer mentioned, the influence of the adhesion between the UV tape and graphene is essential. Thus, we have added the change of the adhesion force of our UV tape before and after the UV illumination in Method section. Although the UV light significantly decreases the adhesion force, there still exists adhesion force between the tape and graphene. This remaining force is very important, avoiding the graphene to slip on the tape surface when applying the tension. This remaining adhesion force is used to apply tensile strain in the graphene, resulting in the formation of GNSs. At the same time, this remaining force is weak enough to allow releasing the graphene on the target SiO_2 substrate.

Indeed, swelling of the support polymer film (polyolefin) can occur by contact with solvents, such as acetone and toluene, while swelling does not occur when only the adhesion layer is wetted with solvent. Therefore, we dropped a small amount of solvent only on the adhesive layer, avoiding direct contact with the support film. This has been also added in Methods section.

Pages 15 of the revised manuscript:

"Although the MoS_2 monolayer is thicker than monolayer graphene, the tensile strain applied to graphene via the UV tape and the liquid intercalation between graphene and tape surface can effectively assist the formation of a nanoscroll structure. Due to the large diameters of the GNSs, this core-shell scroll showed a collapsed structure, but this method can be used to produce high-order van der Waals superlattices consisting of alternating 2D materials.[46]"

Page 20 of the revised manuscript (Methods section):

"The chemical composition and preparation method of the UV tape are reported in ref. 29. First, we adhered the UV tape onto as-grown graphene and illuminated with a UV-light emitting diode (LED) lamp (Quark Technology Co. Ltd., Japan) to harden the adhesive layer, which reduced the adhesion force to graphene. The UV-LED illumination condition was ~93 mW/cm^2 for 11 sec (the total energy is approximately 1000 mJ/cm^2). Upon this illumination the adhesion force of the UV tape decreased from 1.2 N to 0.15 N for a 10 mm tape width against Si wafers. The electrochemical delamination of the tape/graphene stack from the Cu surface was performed in KOH aqueous solution, followed by washing with deionized water. Then, the tape/graphene was fixed to mechanical clamps, and extended up to 15% and kept for 60 min (the experimental setup is shown in Supplementary Fig. 1)."

Page 21 of the revised manuscript (Methods section):

"Here, we dropped only a small amount of solvent to the adhesion layer in order to avoid swelling of the underlying support layer (polyolefin)."

Question 3: According to Figures. 1a, S2 and S3, after the tape was stretched, periodic linear cracks oriented perpendicular to the strain direction appeared on the graphene film. By comparing Figures S3a-c and d, it is evident that without external stimulation, the graphene did not curl into GNSs. In the manuscript, the authors primarily focus on analyzing the role of tensile stress, but they do not provide a detailed explanation of how subsequent stimuli, such as organic solvents, influence the scrolling of graphene. While stress is responsible for the formation of periodic cracks, it is the external stimuli that should be regarded as one of a key driving force behind the actual scrolling of graphene into nanoscrolls. The authors should provide a detailed discussion on this issue.

We appreciate the reviewer for this important and essential comment. After careful experiments, we recognized that the intercalation of solvent between graphene and tape adhesive also plays an important role in the nanoscroll formation, as the reviewer noted.

We think that the solvent plays two main roles in the scrolling. First, solvent, such as acetone and toluene, decreases the adhesion force, assisting the detachment of graphene from the tape surface. We confirmed that the adhesion force significantly decreases when the adhesive layer contacts with toluene or acetone. Another role is to directly assist scrolling via solvent intercalation between graphene and the tape surface, as suggested by the reviewer. The combination of the applied strain and solvent intercalation is supposed to assist the nanoscroll formation. We have added the following new explanation and Supplementary Fig. 13. We believe that our revised manuscript has become more scientifically solid thanks to the reviewer's comment.

Pages 8-9 of the revised manuscript:

"To investigate the role of the additional stimuli employed to make GNSs, we measured the adhesion force of the UV tape after different treatment including UV exposure (Supplementary Information Fig. 13). The pristine UV tape showed high adhesion force (0.12 N/mm), and it decreased to 0.015 N/mm after illuminated with UV light. After this UV illumination process, the graphene/tape stack was fixed to the jig for the mechanical extension (Supplementary Fig. 1). Post-UV heat treatment and contact with solvent further decreased the adhesion force down to 0.009 N/mm. Toluene was found to be the most effective among other studied solvents (Supplementary Information Fig. S13c). Therefore, we treated the graphene/tape stack with toluene while heating it on a hot plate to detach graphene from the tape surface, which induces the scrolling of graphene. We think that the contact with solvent not only reduces the adhesion force of the UV tape but also stimulate the scrolling via intercalation between the strained graphene and tape surface, because the liquid intercalation is widely used to make graphene scrolls.[14,18,28] However, as we used the strained graphene to the pre-designed direction, we can realize a large array of aligned GNSs with controlled scrolling direction

and chirality."

New supplementary figure (Supplementary Fig. 13):

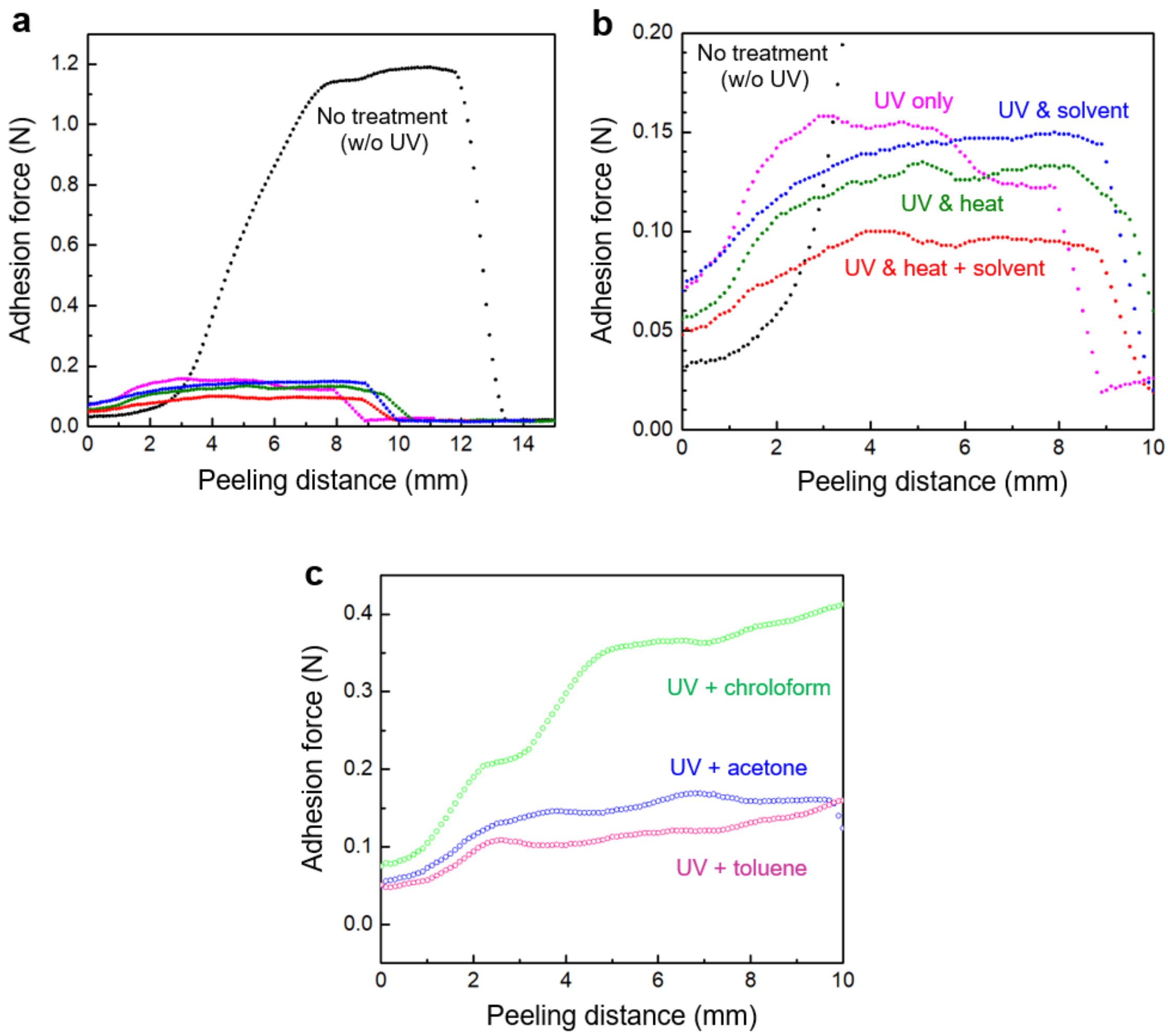

Supplementary Fig. 13. Influence of the post-UV treatment on the adhesion force of the UV tape **a**, Comparison of adhesion force of UV tapes measured after different treatments. The adhesion force was measured by removing the treated UV tape from a bare SiO_2/Si substrate (10 mm width). No UV illumination (black) maintained strong adhesion force, while after UV illumination the adhesion significantly decreased (pink). The drop of solvent (acetone) slightly decreased the adhesion force (blue), but the heat treatment (80 ºC) was more effective to decrease the adhesion force (green). Interestingly, combining the heat treatment and solvent showed the lowest adhesion force (red). **b**, Solvent dependence of the adhesion force (without heat treatment). To synthesize GNSs from the strained graphene, we used toluene and acetone, which are more effective than chloroform.

As the combination of heat and solvent reduces the adhesion force, the solvent can also contribute to effective detachment of graphene from the tape surface. In addition, intercalation of solvent to the graphene-tape interface can assist the formation of GNSs.

Question 4: As the authors regulated the adhesion of the tape by UV illumination and achieved the curling of graphene through strain-engineering and external stimuli, the potential for polymer residue from the tape on the graphene surface must be considered. This issue should be taken into consideration, ideally with supporting characterization data.

In our previous paper (ref. 29, *Nat. Electron.* **7**, 119 (2024)), we confirmed that the UV tape-transferred graphene has a cleaner surface than that transferred by PMMA film. As the main component of the adhesive used in the UV tape is cross-linked block copolymer, there is less chance to leave isolated polymer chains on graphene surface. In contrast, single-chain polymers, such as PMMA, tend to remain on the graphene surface. Thus, the UV tape basically enables a very clean transfer.

To confirm the cleanliness, we have measured XPS. Table R3-1 compares the surface composition of the tape surface and GNS. While the UV tape contains 4% N atoms, we did not observe N_{1s} signal on the GNSs/SiO_2, indicating the tape residue is below the detection limit.

However, as compared with the standard, previously reported UV tape transfer method, there is a still chance to have the residue of the adhesive on GNSs, because we introduced organic solvent to assist the nanoscroll formation. Even this, a small amount of tape residue can be mostly removed by soaking in acetone and H_2 annealing processes. As shown in Table R3-1 (below), N atoms existing in the UV tape was not detected on the GNS sample, indicating the tape residue can be removed efficiently. This agrees with the clean interfaces of the scrolls observed by STEM.

Table R3-1. XPS elemental analysis of the UV tape surface and GNSs on a SiO_2 substrate. Atomic concentrations (%) of each sample are displayed.

	C	N	O	Si	Cu
UV tape surface	78.4	4.0	17.6	—	—
GNS/SiO_2	8.6	—	58.0	33.4	—

Question 5: The authors claim the ability to synthesize GNSs with controlled chirality (zigzag, armchair, chiral) by simply adjusting the stretching direction of the tape/graphene stack (Lines 114-118, Fig. 1c-e). This claim is primarily inferred from the optical microscopy images (Fig. 1c-e) and the known relationship between crack orientation and strain direction. However, direct atomic-scale evidence linking the prescribed stretching direction to the resulting edge structure of the final GNS is lacking. It is recommended to employ techniques such as STEM with high resolution to directly visualize the edge atomic structure of individual scrolls synthesized under different strain orientations. This will provide crucial validation for the proposed control over GNS chirality.

This reviewer's comment is very important, and indeed the optical microscope images of GNSs are not sufficient to claim the control of chirality, which is one of the crucial points of our manuscript. Therefore, we have performed additional TEM and STEM measurements for the three types of GNSs (zigzag, armchair, and chiral). Due to the overlapping of the graphene layer, it was difficult to find edges of GNSs. However, the Fast Fourier Transform (FFT) analysis clearly verified that the GNSs have chiralities determined by the stretch directions. The result has been added in Fig. 1g-i and Supplementary Fig. 4. The three scrolling directions are confirmed to be determined by the directions of the applied tensile strain. Although the solvent intercalation also contributes to the nanoscroll formation, the applied strain mainly determines the chirality via crack formation. It is likely that the residual strain and parallel cracks contribute to the chirality control.

New Fig. 1c,g-i:

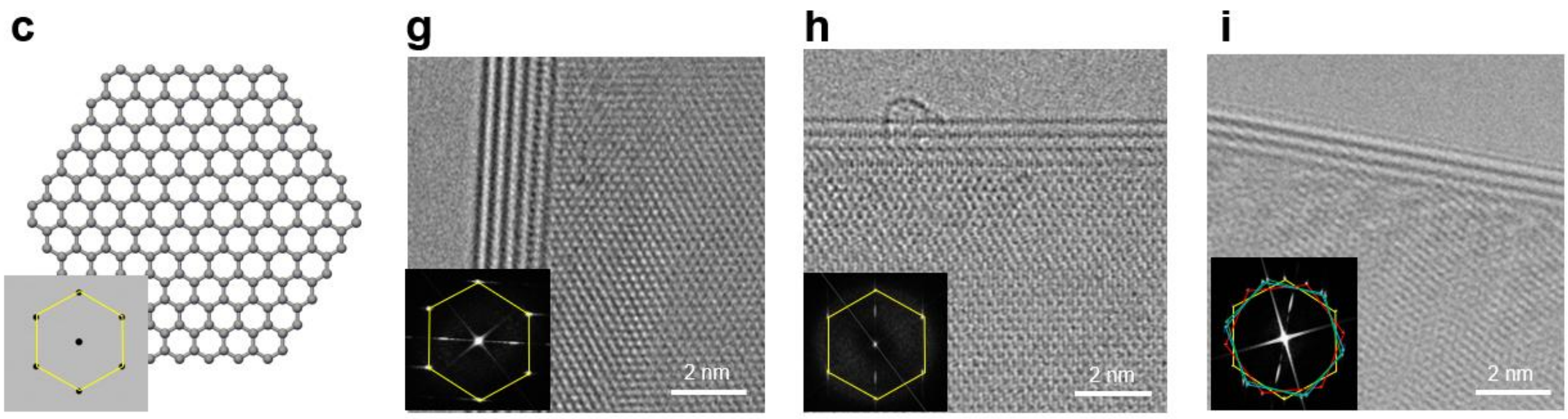

Fig. 1. c, Schematic atomic structure and corresponding simulated diffraction pattern (inset) of the hexagonal grain shown in **b. d-f**, GNSs made by stretching the UV tape/graphene stack along the directions indicated by the arrows. (**d**) zigzag GNSs, (**e**) armchair GNSs, and (**f**) chiral GNSs. **g-i,** Top-view high-resolution TEM images of the corresponding GNSs. Insets are the FFT patterns.

Pages 5-6 of the revised manuscript:

"To confirm the chirality-selective formation of nanoscrolls, the GNS samples were further characterized using high-resolution electron microscopes. Fig. 1g-i display the top-view transmission electron microscopy (TEM) images of the nanoscrolls, together with their corresponding FFT patterns shown in the insets (the corresponding high-resolution scanning transmission electron microscopy (STEM) images are displayed in Supplementary Fig 4). In Fig. 1g-i, we clearly observed the layered structure reflecting the rolling structure. The FFT patterns shown in the insets confirmed the formation of zigzag and armchair nanoscrolls, which are solely determined by the stretch directions. It is worth noting that it is difficult to grow chirality-controlled carbon nanotubes, while our unique tensile-based method allows to make zigzag and armchair scrolls in a highly controlled manner. In addition, due to the scrolling structure, all the rolls have the same chirality, which is completely different from a MWCNT in which each layer has different chiralities.[19] Chiral GNS exhibited interesting FFT patterns (inset of Fig. 1i); the number of the set of spots almost corresponding to the number of rolls. Although the origin of the multiple patterns is not clear, it may reflect a cone-like chiral nanoscroll structure. The possibility of the chirality control, including zigzag and armchair, is an advantage of our strain-assisted nanoscroll formation, which offers new opportunities to explore chiral-related phenomena."

New Supplementary Fig.4:

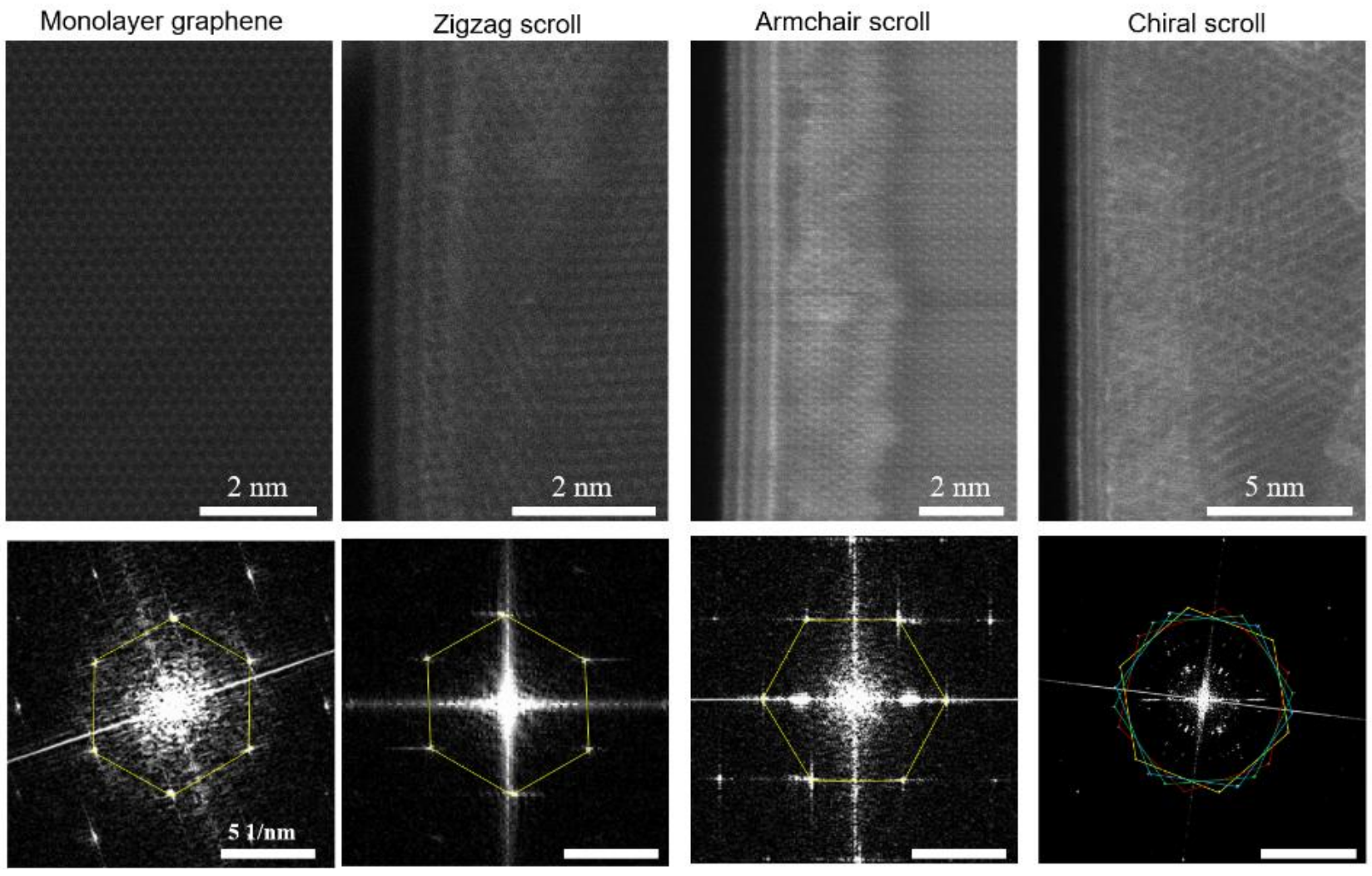

Supplementary Fig. 4. High-resolution STEM images of monolayer graphene and GNSs rolled with controlled chirality. Upper panels show the STEM images, and lower panels show the corresponding FFT patterns.

Question 6: The SEM image (Figure 3) interpretation might be ambivalent (Line 184-192). We observe that a GNS like contrast is already present on the substrate. It is not clear, if this structure is displaced or if an already formed GNS continues to roll up.

Although it is difficult to capture high resolution image with our *in-situ* SEM system, it is apparent that the graphene ribbon sitting on a SiO_2 surface has a width larger than 1 μm (Fig. R3-2). As GNSs have a few hundred nm widths (please refer AFM image shown in Fig. 1n of the revised manuscript), the SEM image in Fig. R3-2 indicates the presence of a wide ribbon on a SiO_2 surface (not a nanoscroll).

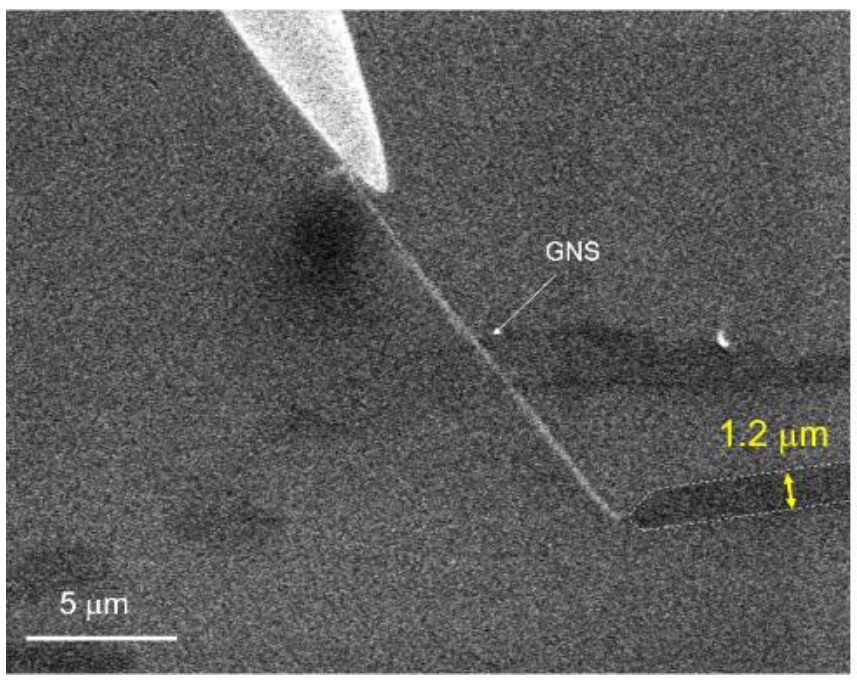

Fig. R3-2. SEM image of a GNS formed from a graphene ribbon with 1.2 μm width.

Question 7: Is the description of the experimental observation during the GNS formation complete? A challenge in such processes is the competition between complete delamination/rolling and fracture into smaller fragments. To demonstrate a complete understanding of the process and establish a convincing rationale for the high yield of GNSs reported, it should be clarified what specific measures were taken to decisively favor the scrolling pathway over fracture.

We acknowledge the comment. As commented by the reviewer, the formation mechanism of GNSs was not very clear in the original manuscript. Therefore, for the revised manuscript, we performed measurements of the crack density while changing tensile strain to a graphene/tape stack. As shown below (new Fig. 2 and Supplementary Fig. 9a), we succeeded in observing an increase of the crack density with the tensile strain. At low strains (up to 2.5%) the crack density was rather low, but increasing the strain to 5% induced more evident cracks with higher crack density. The cracks became longer and clearer by further increasing the strain, contributing to the formation of a number of aligned graphene ribbons. The crack density plotted against the applied strain (Fig. 2c) almost saturates at 10%. Therefore, increasing the strain in the revised version of the manuscript to >10% from the original 5% allowed us to increase the GNS density, obtaining more uniform and denser GNS arrays.

As each graphene nanoribbon produces one GNS, the GNS density is almost equivalent to the density of the ribbons. The chiral angle is determined by the stretch direction, because the residual strain acts as one of the driving forces to make nanoscroll structure. Scroll angle (*i.e.*, chiral angles) of GNSs have been newly measured by TEM and STEM, as shown in new Fig. 1g-i of the main manuscript and Supplementary Fig. 4. The fast Fourier transform (FFT) patterns confirm that the chiral angle can be determined by the direction of the extension.

In addition, we monitored the actual tensile strain in graphene by *in-situ* Raman spectroscopy. The results are shown in Supplementary Figs. 11 and 12.

New figure (Fig. 2):

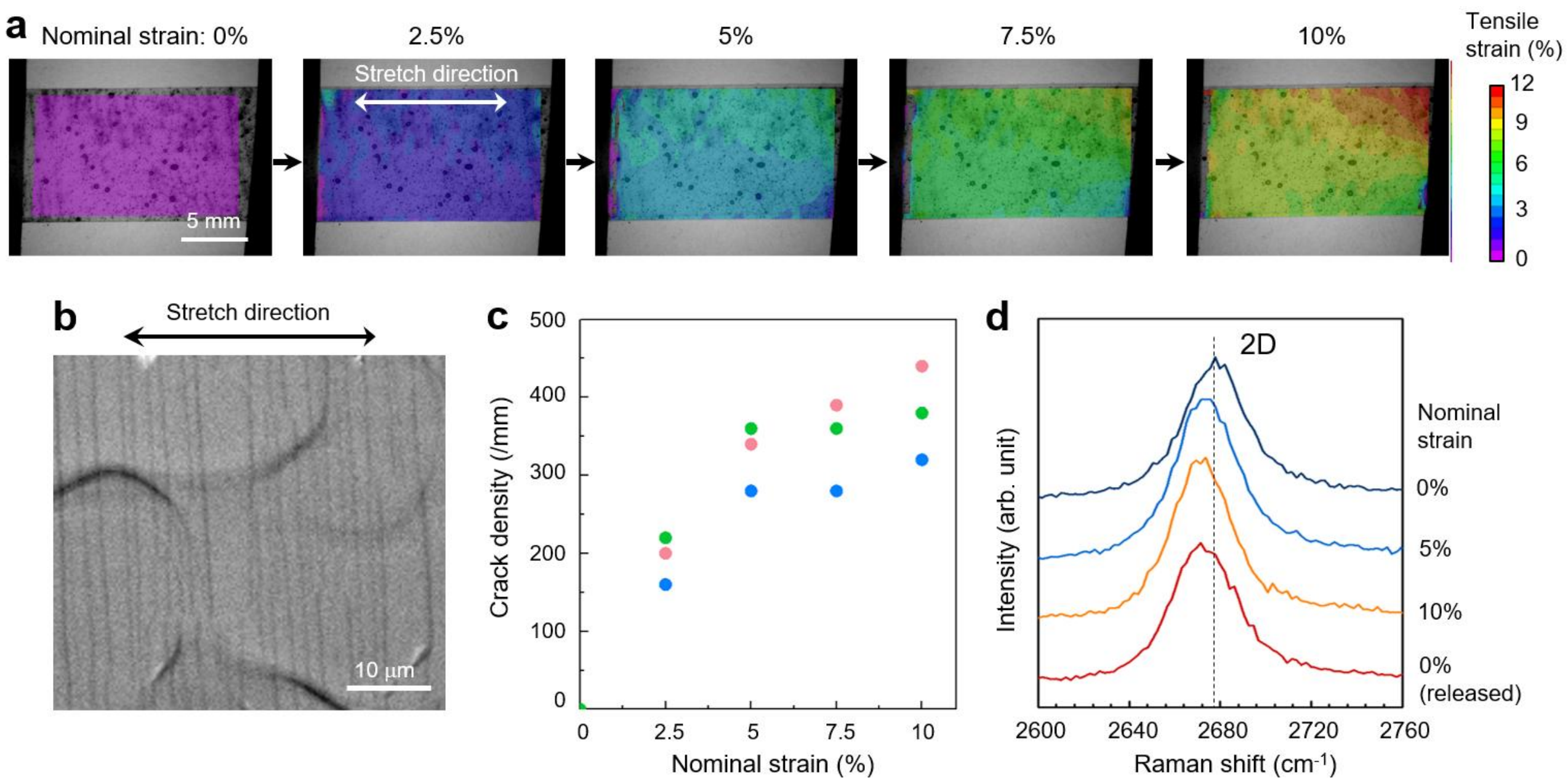

Fig. 2. *In-situ* measurement of tape deformation, crack formation, and Raman spectra. a, DIC analysis of the actual tensile strain applied to the graphene/tape stack. The nominal strain is calculated from the change of the tape length, and tensile strain was determined from the local changes of each position of the stack. **b**, Laser confocal microscope image of the graphene on the UV tape, captured while applying a nominal strain of 10%. **c**, Evolution of crack density of graphene with nominal tensile strain, measured at three different positions. **d**, *in-situ* Raman spectra measured while applying the strain, and after releasing the nominal strain applied to the stack.

New supplementary figure (Supplementary Fig. 9):

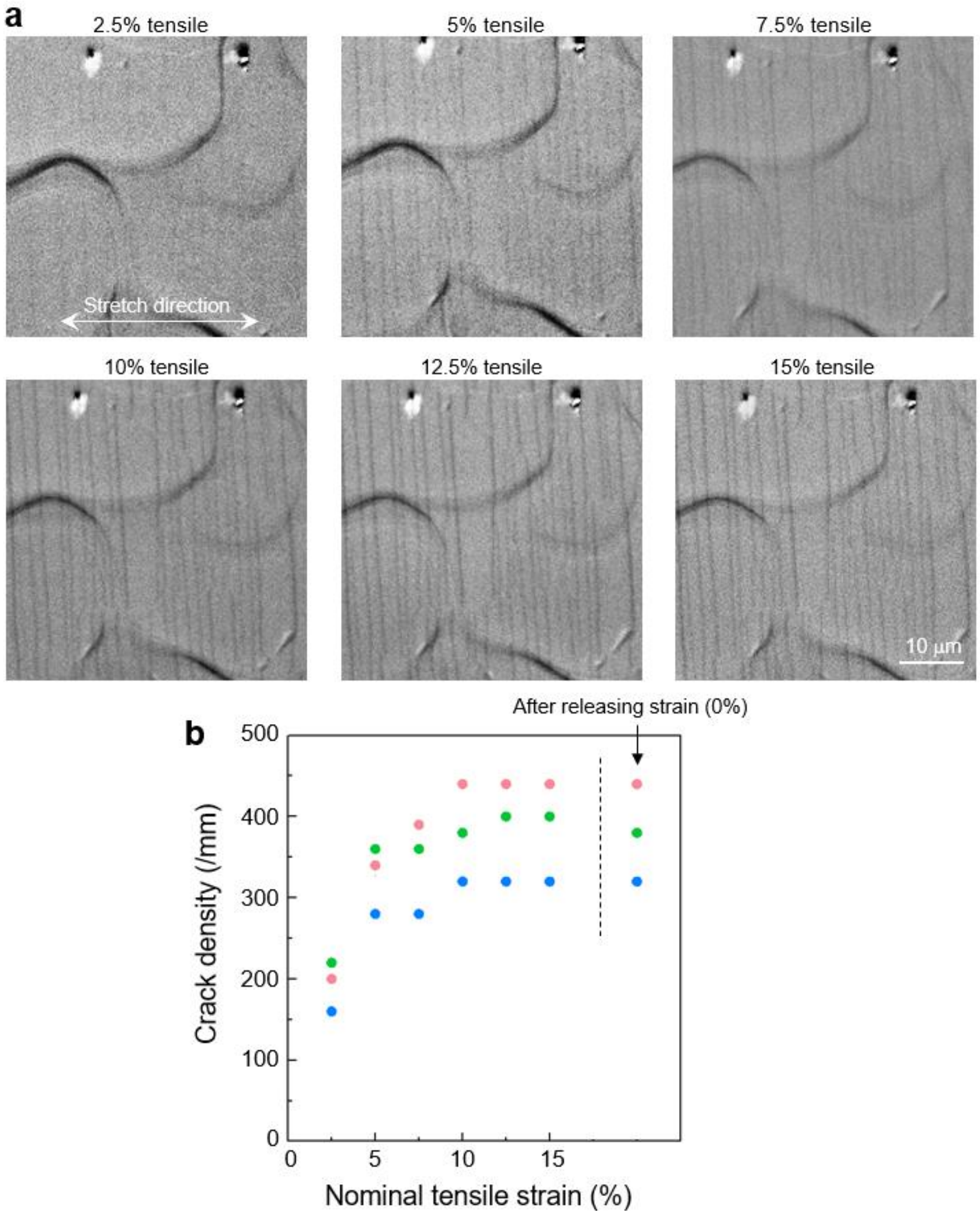

Supplementary Fig. 9. Crack formation while applying strain to the UV tape. a, Confocal laser microscope images of monolayer graphene on the UV tape captured under different applied strains (the strain values are the nominal values calculated from the change of the tape length). To monitor the crack formation, we captured images at identical positions. It is clearly seen that cracks are formed by extending the UV tape, with the number of cracks increasing with strain. **b**, Crack density plotted against the applied tensile strain. The plot shows three different positions of the stack, indicating a similar trend regardless of the area. The crack density practically saturates at ~10% strain.

Question 8: Please explain in more detail which in Figure S9, where the structure collapsed. The geometry is not clear form figure or text.

To assist general readers, we have added an illustration to explain the cross-sectional TEM images in

the inset of Supplementary Fig. 16b. We think that this illustration will help to understand the images.

Supplementary Fig. 16b:

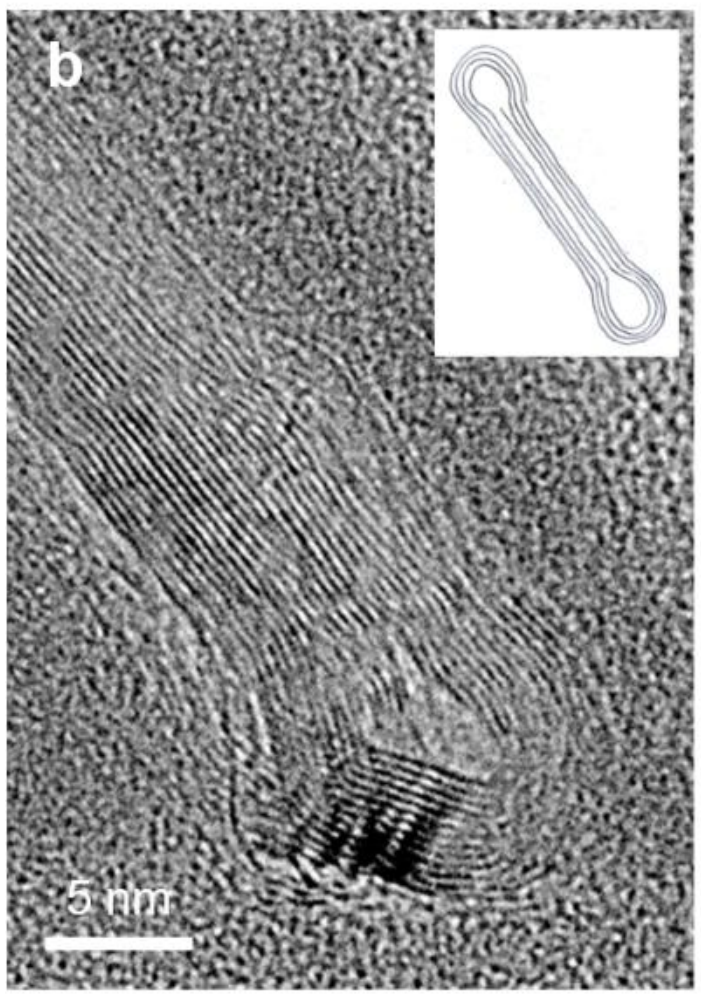

Supplementary Fig. 16. b, High magnification TEM image. This GNS collapsed due to the large diameter, but two sides exhibit the curved structure, representing the nanoscroll is collapsed to make intra-layer van der Waals interaction. This GNS is tilted with respect to the SiO_2 surface, indicating that the GNS is mechanically stabilized with many scrolled graphene layers. The inset is a schematic of the collapsed nanoscroll.

Question 9: In Figure 3f, we obtain quite consistently 3.7 Å interlayer spacing, not the reported 0.34 nm. This brings the lattice plane spacing of the GNS in this work into the range of the ones observed by others. And the strain driven part of the scrolling process might not be very different from the impact of strain in other GNS work. Also, the authors report that, similar to other works, the scrolling result is best in the presence of solving. Therefore, we believe that in the modelling, the pressure coming from surface tension must be considered as a second factor. Or, if there is only a non-specified pressure used in the system, the share between pressure coming from surface tension and from the straining procedure should be calculated (Line 196-197).

We thank the reviewer for pointing out this. We have checked the original image and confirmed that the intra-layer distance is 0.34 nm. We corrected the length of the scale bars of Fig. 3m,n. We also confirmed the intra-layer distance of 0.34 nm from the new TEM images shown in Fig. 1g-i.

Question 10: line 218: "unstable..." . We believe that the strain might not be the only mechanism causing the rolling effect. A solvant is used and surface tension can contribute to the formation of a 3nm GNS.

We acknowledge this comment. As the role of solvent intercalation have already been discussed in Question 3, we kindly ask the reviewer to check our reply to Question 3.

Question 11: In the section of "Electrical characterization and intercalation", the fabrication process of the device must be clearly outlined. where is Photolithography, where is electron beam deposition used?

Thank you for the comment. By responding to this reviewer's comment, a more detailed fabrication process has been added in Methods section.

.

Page 22 of the revised manuscript:

"For the fabrication of FETs with a GNS or a heteronanoscroll channel, we used photolithography to make the pattern for metal electrode deposition. Doped silicon substrates with 300 nm top SiO_2 layer were used. We deposited two types of metal electrodes (Au (30nm)/Ni (10 nm) and Au (30 nm) only) by electron beam evaporation, followed by lift-off with acetone."

Question 12: Is a hysteresis observed when reducing V(drain)? (Line 272-280)

To respond to this reviewer's question, we measured three GNS transistors with three different drain voltages, 100, 10, and 1 mV in vacuum. As shown in newly added Supplementary Fig. 21, all the three devices exhibited clear hysteresis regardless of the applied drain voltage. This indicates that the hysteresis is an intrinsic property of the GNS transistors and not due to measurement conditions. We have added the new Supplementary Fig. 21 and the following comment in the revised manuscript.

Page 21 of the revised manuscript:

"The hysteresis was almost independent of the applied drain voltage, as shown in Supplementary Fig. 21, which is consistent with gate-controlled charge trapping processes."

New supplementary figure (Supplementary Fig. 20):

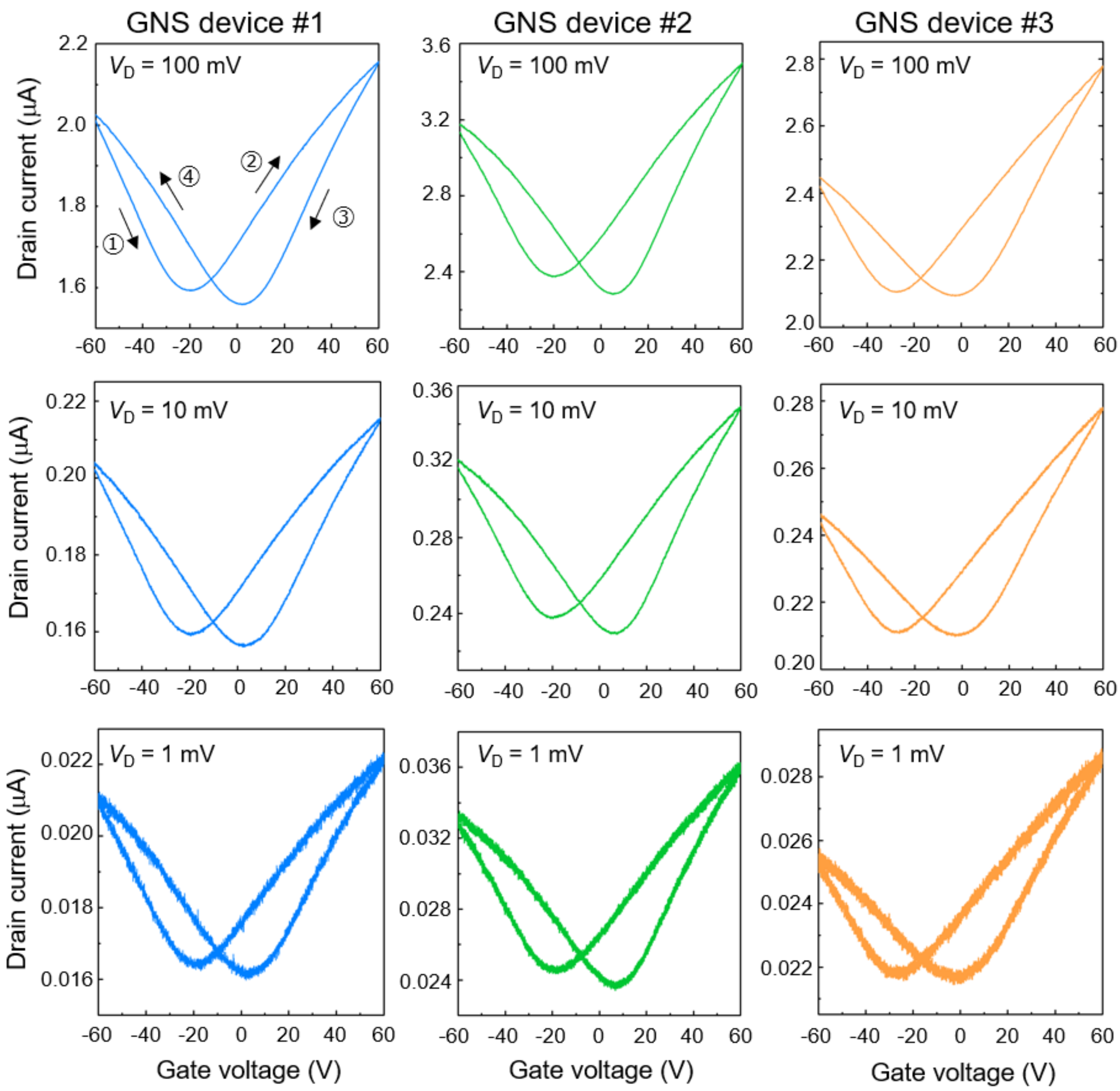

Supplementary Fig. 21. Drain voltage dependence of GNS-FETs. Transfer curves of three GNS-FETs measured at three different drain voltages (100, 10, and 1 mV). Hysteresis was observed regardless of the applied drain voltage.

Question 13: Line288-289, here, the current density needs to be mentioned, i.e. the diameter of the GNS needs to be reported.

As pointed by the reviewer the information of the diameter is important to calculate the current density. In Supplementary Fig. 20c,d (originally Supplementary Fig. 11c,d), we displayed the AFM image and the corresponding height profile the GNS used for the breakdown experiment. As shown in Supplementary Fig. 20d, the GNS has a height of 27 nm, which gives a higher current density (2.3

$\times 10^8$ A/cm^2) than the previously reported value for the GNS (5×10^7 A/cm^2 (ref. 14)) with the diameter of 50 nm.

Page 16 of the revised manuscript:

"The device stands a high drain voltage up to ~14 V, and combined with the AFM measurement (Supplementary Fig. 20), the maximum current density was estimated to be 2.3×10^8 A/cm^2 for the GNS with a diameter of 27 nm. This value is higher than the previously reported values for a GNS (5×10^7 A/cm^2)[14] and an order of magnitude higher than that of Cu metal (10^6 A/cm^2). In the case of MWCNTs, the layer-by-layer breakdown from outer walls induces the step-like oxidation against the drain voltage.[19] However, the abrupt decrease of the current in our GNS device suggests that the whole graphene layer contributes to the current flow."

Supplementary Fig. 20:

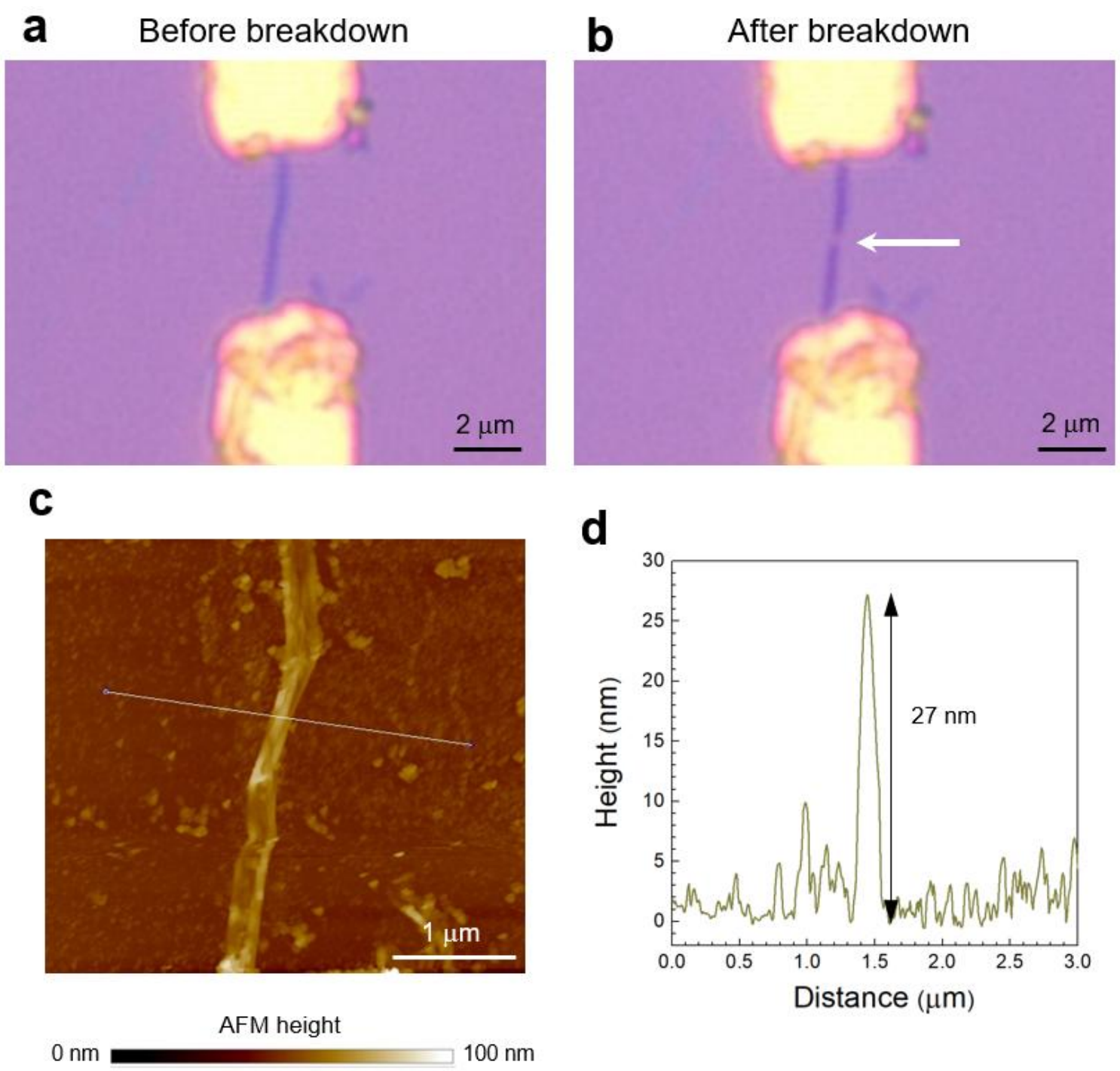

Supplementary Fig. 20. Breakdown measurement of a GNS. Optical image of a GNS measured before (**a**) and after (**b**) breakdown for the device shown in Fig. 5b. The breakdown occurs at the point marked by the white arrow. **c**, AFM image of the GNS measured before the breakdown. **d**, Cross-section of the GNS measured along the line shown in **c**. Note that the actual maximum current density can be higher than the calculated value, because the GNS has a hollow structure.

Question 14: line 298 ff, EDX and EELS spectra should be shown.

We appreciate the reviewer for noticing this. We have corrected these errors.

Page 14 of the revised manuscript:

"The SEM image and the corresponding elemental mappings measured by energy dispersive x-ray spectroscopy (EDX) of a co-scroll are shown in Fig. 5c. These images confirm that both graphene and MoS_2 are present along the longitudinal-axis of the nanoscroll. The cross-sectional high-angle annular dark field (HAADF) image and the corresponding electron energy loss spectroscopy (EELS) elemental mapping (Fig. 5d and Supplementary Fig. 18) along with the line profiles for the HAADF, C and Mo in Fig. 5e, further prove that both graphene and MoS_2 rolled up together, realizing a heteronanoscroll with alternating graphene and MoS_2 layers."

Question 15: The terminology in some sections of the manuscript lacks precision. For example, "Raman and cross-sectional electron microscopy" (Line 36) should likely be specified as "Raman spectroscopy and cross-sectional transmission electron microscopy". Additionally, the abbreviation "MD" is used without being defined. Do the authors use MM and MD for the same simulation technique used in this work? Please thoroughly review and revise the relevant descriptions throughout the manuscript to ensure terminological accuracy and professionalism.

Thank you for pointing out the typos in the original manuscript. As for "Raman and cross-sectional electron microscopy", we removed this sentence from the abstract, which was revised to account for the additional experiments and results of the revised manuscript.

About the MM and MD calculations, we performed both MM and MD calculations for different purposes. The MM calculation was used to analyze energetics of the GNSs with different inner diameters, while MD calculation was used to visualize the dynamics of rolling process of the strained graphene sheet. The MM calculation result is shown in Fig. 4c-e, and the MD result is presented in Fig. 4f-k and Supplementary videos 4-6.

Page 13 of the revised manuscript:

"To further understand the mechanism behind the phenomenon of rolling, we performed molecular dynamics (MD) calculations to simulate the nanoscroll formation, with different initial conditions (see Methods section)."

Question 16: In literature, we found only deformation of graphene adhered to tape or polymer is in the range of 1-3 %, not the 5% reported here, see for example Guo et al, Adv Mat Interfaces, 10, 2201531, 2023. The 5% reported here seem extraordinarily high. The authors should explain, how they reach this very high value and prove that the graphene stays really adhered to the polymer up to a strain of 5% of the graphene sheet, i.e. not 5% strain of the tape. (Line 93-97)

We acknowledge the reviewer for this comment, and apologize for the confusion caused by the original version. As the reviewer commented, applying a large strain of 5% is not possible in 2D materials, including graphene. The strain used throughout the manuscript is the nominal value calculated from the change of length of the UV tape. As can be seen in the new Supplementary Fig. 9 (please refer reply to question 7), a number of parallel cracks are formed in the graphene during when straining the tape/graphene stack. These cracks reduced the applied strain to graphene. The *in-situ* Raman measurement (Supplementary Fig. 12) presents the evolution of actual strain induced to graphene. We also observed that crack formation reduced the actual strain when the stack was stretched over 2%. We have added this discussion in Supplementary Fig. 12.

New supplementary figure (Supplementary Fig. 12):

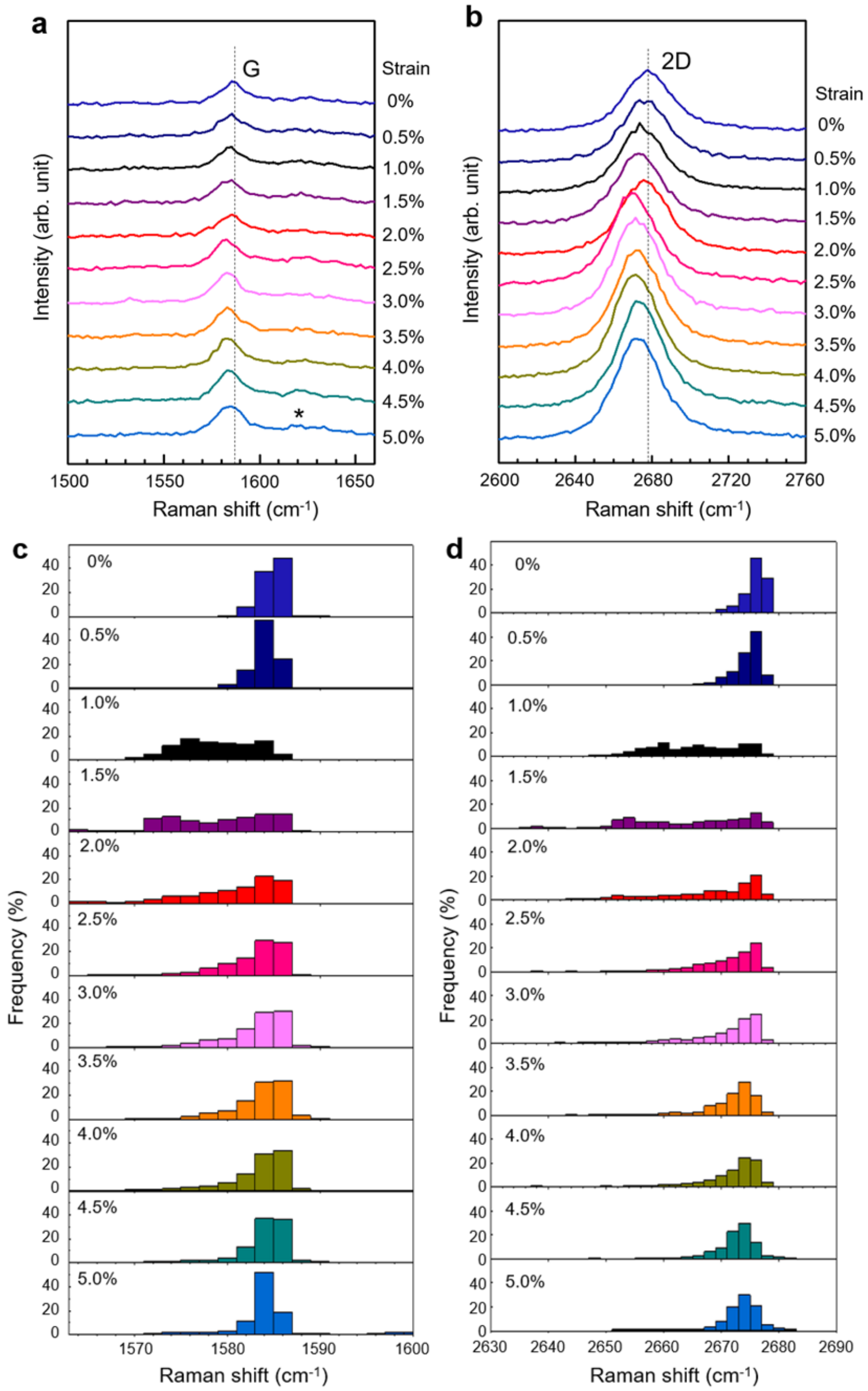

Supplementary Fig. 12. Raman spectra of graphene on the UV tape under low strain loads. a,b, Raman spectra of monolayer graphene measured while applying tensile strain (up to 5%) to the graphene/tape stack: G band (**a**) and 2D band (**b**). The strain values are the nominal values applied to the UV tape. Asterisk in (**a**) represents a peak from the tape. **c,d,** Distributions of the positions of G band (**c**) and 2D band (**d**).

Question 17: line 158: Did you only measure graphene thickness by RS, or did you, in addition measure height on graphene flakes transferred to for example Si substrates by AFM?

We used AFM images to measure diameters of GNS after transferring on SiO_2 substrates. Thickness was only measured by TEM/STEM (Fig. 3m,n, and Supplementary Fig. 16). Basically, Raman spectroscopy does not provide accurate information on the diameter and thickness of GNSs.

Reviewer #4 (Remarks to the Author):

I co-reviewed this manuscript with one of the reviewers who provided the listed reports. This is part of the Nature Communications initiative to facilitate training in peer review and to provide appropriate recognition for Early Career Researchers who co-review manuscripts.

We acknowledge the reviewer for spending time to review our manuscript and giving us valuable comments.

Reviewer #5 (Remarks to the Author):

I co-reviewed this manuscript with one of the reviewers who provided the listed reports. This is part of the Nature Communications initiative to facilitate training in peer review and to provide appropriate recognition for Early Career Researchers who co-review manuscripts.

We thank the reviewer for reviewing our manuscript and giving us valuable comments, which helped us to improve the quality of our manuscript.
